# Opioid antagonists are associated with a reduction in the symptoms of schizophrenia: a meta-analysis of controlled trials

**DOI:** 10.1038/s41386-020-0730-z

**Published:** 2020-06-09

**Authors:** Samuel D. Clark, Jared X. Van Snellenberg, Jacqueline M. Lawson, Anissa Abi-Dargham

**Affiliations:** 1 Columbia University Medical Center, New York, NY USA; 2 Terran Biosciences, New York, NY USA; 3 Department of Psychiatry and Behavioral Health, Stony Brook University Medical Center, New York, NY USA; 4 Department of Psychology, Stony Brook University, New York, NY USA; 5 Department of Biomedical Engineering, Stony Brook University, New York, NY USA; 6 Department of Radiology, Stony Brook University Medical Center, New York, NY USA

**Keywords:** Schizophrenia, Psychosis

## Abstract

Current treatments for the symptoms of schizophrenia are only effective for positive symptoms in some individuals, and have considerable side effects that impact compliance. Thus, there is a need to investigate the efficacy of other compounds in treating both positive and negative symptoms. We conducted a meta-analysis of English language placebo-controlled clinical trials of naloxone, naltrexone, nalmefene, and buprenorphine in patients with schizophrenia to determine whether opioid antagonists have therapeutic efficacy on positive, negative, total, or general symptoms. We searched online databases Ovid Medline and PsychINFO, PubMed, EMBASE, Scopus, Cochrane library/CENTRAL, Web of Science, and Google Scholar from 1970 through February 2019. Following PRISMA guidelines, Hedges *g* was calculated for each study. Primary study outcomes were the within-subject change on any symptom assessment scale for positive, negative, total, or general symptoms of schizophrenia between active drug and placebo conditions. Thirty studies were included with 434 total patients. We found a significant effect of all drugs on all scales combined with both a standard random effects model: (*g* = 0.26; *P* = 0.02; *k* = 22; CI = 0.03–0.49) and a more inclusive bootstrap model: (*g* = 0.26; *P* = 0.0002; k = 30; CI = 0.11–0.51) and a significant effect on total scales with the bootstrap model (*g* = 0.25288; *P* = 0.015; *k* = 19; CI = 0.04–0.35). We also observed a significant effect of all drugs on all positive scales combined with both the random effects (*g* = 0.33; *P* = 0.015; *k* = 17; CI = 0.07–0.60) and bootstrap models (*g* = 0.32; *P* < 0.0001; *k* = 21; CI = 0.13–1.38). This evidence provides support for further testing in randomized clinical trials of a new class of non-D2-receptor drugs, based on opioid mechanisms, for the treatment of positive and negative symptoms of schizophrenia.

## Introduction

Schizophrenia is a chronic psychiatric syndrome with an estimated worldwide prevalence of 0.749% [1] and total annual cost upwards of $63 billion in the United States alone [2, 3]. There is a need to find new treatments for the positive and negative symptoms of schizophrenia, as up to 30% of patients do not fully respond to antipsychotics [4], and while non-pharmacological treatments have been shown to have some benefit [5–7], there are currently no approved therapies for negative symptoms.

Despite a pressing need for novel therapeutics, there has been a paucity of investigations into a potentially relevant target, the endogenous opioid system, which includes three receptors subtypes: mu, kappa, and delta. This contrasts with a substantial literature of clinical trials spanning back to the 1970s, documenting possible efficacy of opioid antagonists on the symptoms of schizophrenia. This literature began in 1977, with a single blind placebo-controlled study showing an antipsychotic effect of naloxone in patients with schizophrenia [8]. Later work attempted to replicate these findings using three different pan-opioid (kappa, mu, and delta) antagonists (naloxone, naltrexone, and nalmefene), and the mixed kappa antagonist and mu partial agonist buprenorphine, with various clinical scales as endpoints. Results have been mixed, with outcomes ranging from significant improvements to nonsignificant trends or no effect. However, these trials had large differences in study design and quality, with many studies substantially underpowered (average *N* = 14.4), making it difficult to derive from narrative reviews [9–11] definitive insights concerning the potential therapeutic effects of these compounds. This prompted us to undertake the first meta-analysis of opioid antagonist trials in schizophrenia to more definitively assess a potential therapeutic signal. We sought to answer the question of whether these opioid antagonists have therapeutic efficacy in patients with schizophrenia.

## Methods and materials

### Protocol

This meta-analysis was conducted according to the published Preferred Reporting Items for Systematic Reviews and Meta-Analyses (PRISMA) guidelines [12]. Although a formal protocol document was not registered prior to beginning the meta-analysis, all inclusion/exclusion criteria and outcome measures were decided prior to initiating the review. Outcome measures were selected as being any symptom outcome (positive, negative, disorganized, general or total symptom assessments) reported by at least six published studies. Certain analyses were selected after commencement of data-gathering procedures in order to accommodate the data in identified studies (i.e., the bootstrapped maximum-likelihood model to accommodate the considerable number of studies not reporting sufficient data for effect size estimation, the compound- and timepoint-specific sub-analyses, and moderator analyses). As with our prior meta-analyses [13–15], all data analysis was conducted in Matlab, using methods described below.

### Eligibility criteria

We included placebo-controlled clinical trials of naloxone, naltrexone, nalmefene, or buprenorphine in patients with a diagnosis of schizophrenia, schizophreniform, of schizoaffective disorder, of any length of follow-up, conducted from 1970 through February 2019, and published as a journal article in English. Studies were required to be single or double blind but were not required to be randomized (although many specified that they were). Study characteristics (PICO) are detailed in Supplementary Tables S6, S7. Finally, studies must have reported the effect of active drug versus placebo on symptoms of schizophrenia, our outcome of interest (positive, negative, total, or general).

### Information sources and study selection

**Fig. 1:**
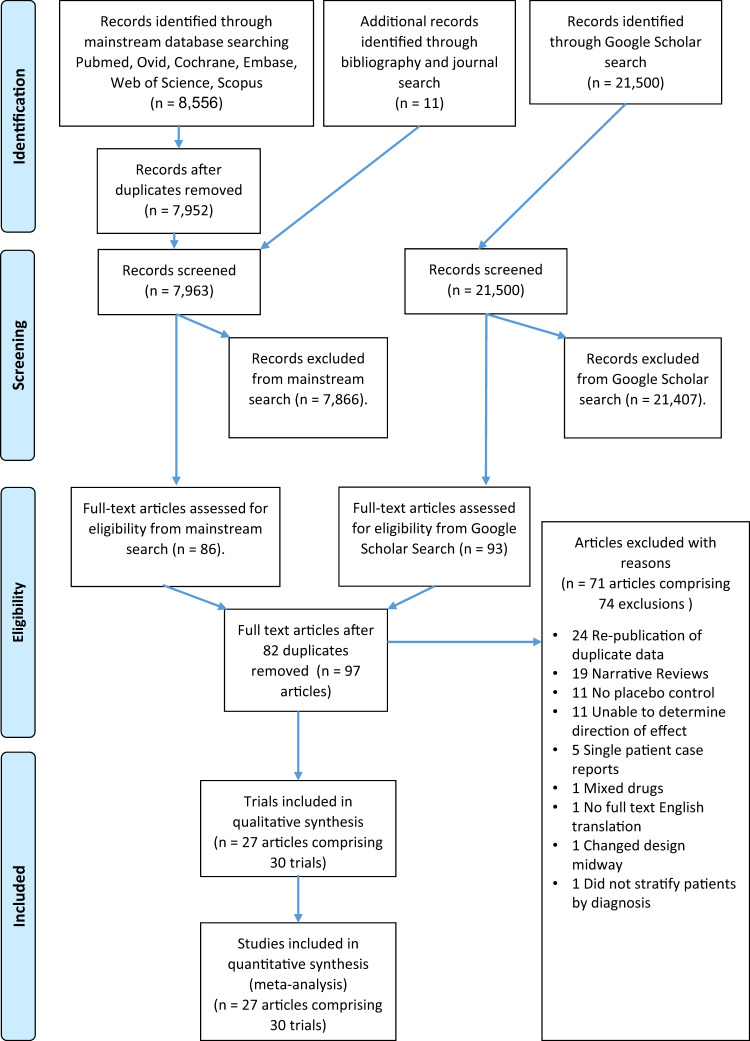
PRISMA flow diagram. Unlike the main databases, Google Scholar does not permit mass export of all citations and thus it is not feasible to identify duplicates from the main database search. Thus Google Scholar search results were evaluated separately and duplicates were removed at the next step. Because several old publications contained multiple separate independent studies of various quality, each study was evaluated for inclusion separately. In the case a paper reported two independent studies such as a high- and a low-quality study, the high-quality study was included and the low-quality study excluded with reasons. This resulted in the paper appearing on both the exclusion list and the inclusion list.

We performed a search of online databases Ovid Medline PsychINFO, PubMed, EMBASE, Cochrane library/CENTRAL, Scopus, Web of Science, and Google Scholar from 1970 through February 2019. Using the keywords schizophrenia and naloxone, or naltrexone, or nalmefene, or buprenorphine. We also performed a search of all of the bibliographies in the trials we identified. Finally, we performed a search of the following journals: American Journal of Psychiatry, Biological Psychiatry, Schizophrenia Bulletin, Schizophrenia Research, and Science. The PRISMA flow diagram of this search is shown in Fig. 1. The PRISMA checklist is reported in Supplementary Table S1 and the full search strings are reported in Supplementary Table S2. Specific steps taken during the processing of the information sources and study selection are detailed in the Supplement.

### Data collection process and data items

The population of interest was patients with schizophrenia, schizophreniform, or schizoaffective disorder. The intervention was any dose of naloxone, naltrexone, buprenorphine, or nalmefene. The comparison was saline or placebo tablets. Data from studies meeting eligibility criteria (above) were coded as detailed below. The outcome measure of interest was the numerical change on scales of positive, negative, general, or total symptoms of schizophrenia, following active treatment as compared with placebo. Scales utilized in these trials included the Brief Psychiatric Rating Scale (BPRS) [16]; Scale for the Assessment of Positive Symptoms (SAPS) [17]; Scale for the Assessment of Negative Symptoms (SANS) [18, 19]; Positive and Negative Syndrome Scale (PANSS) [20, 21]; Verhaltens-Beobachtungs-Skala (VBS) [22]; Inpatient Multidimensional Psychiatric Rating Scale (IMPS) [23]; National Institute of Mental Health rating scale (NIMH), also known as the Inpatient Behavioral Rating Scale [24]; and the Comprehensive Psychiatric Rating Scale (CPRS) [25]. We also included studies that used patient self-reported outcomes or “custom scales.” We grouped our analysis of these scales based on the symptoms that each scale was designed to measure (thereby allowing separate meta-analyses of, e.g., positive and negative symptoms), resulting in the categories and the symptom scales that they include detailed in Table 1. We performed an independent meta-analysis for each of the scale categories that had at least six studies reporting results. When a single study reported results on multiple scales that could be included in a single analysis (e.g., for the positive total scales analysis, a study reporting BPRS hallucinations and BPRS unusual thought content, but not BPRS thinking disturbance), effect sizes were averaged to produce a single outcome measure. In addition, we averaged together all outcome timepoints within studies, as there was considerable variability in the time delay after drug administration until symptom scales were assessed. Full details on extraction of information from manuscripts, and further relevant details, are presented in Supplement and Supplementary Table S3.

**Table 1:** Analysis scale groupings.

Name of analysis			Scales included in analysis
All Scales Combined	All Positive Scales Combined		**Positive symptoms subgroups**
		Hallucination Scales	Hallucinations Group
			BPRS Hallucinations
			BPRS Hallucinatory Behavior
			Custom Scale Hallucination Reduction
			NIMH Hallucinations
			SAPS Hallucinations
			Self-Rated Hallucinations
			Self-Rated Distress Caused by hallucinations
		Delusion Scales	Delusions Group
			BPRS Paranoid Suspicion
			BPRS Unusual Thought Content
			SAPS Delusions
		Other Positive Scales	Other Positive Symptom Subscales
			SAPS Bizarre Behavior
			BPRS Thought Disturbance Factor
			BPRS Thought Disorder
			SAPS Thought Disorder
			BPRS Conceptual Disorganization
		Total Positive Scales	Total Positive Scales Group
			SAPS Total
			PANSS Positive
			BPRS Thinking Disturbance
	Total Scales		Total Scales Group
			PANSS General
			BPRS Total
			BPRS Total Excluding Hallucinatory Behavior
			BPRS Schizophrenia Restricted
			CPRS
			IMPS
			VBS
	All Negative Scales Combined	Total Negative Scales	Total Negative Scales Group
			SANS Total
			PANSS Negative
			BPRS Negative Schizophrenic Symptoms
		Other Negative Scales	**Negative symptoms subgroups**
			BPRS Emotional Withdrawal
			BPRS Anergia
			BPRS Withdrawal-Retardation
			SANS Affective flattening
			SANS Apathy
			SANS Inattentiveness
			SANS Poverty of Speech
			SANS Anhedonia

### Effect sizes (summary measures)

Effect size estimates (Hedge’s *g*) [26] for each study were calculated from available data in the following order of preference: (1) mean and SD (or SEM) for the difference between symptom change on drug minus symptom change on placebo; (2) mean and SD (or SEM) for symptom change on drug and on placebo, calculated separately; (3) mean and SD for each of the four conditions when reported separately (i.e., baseline drug, baseline placebo, posttreatment drug, and posttreatment baseline), (4) *t*, *F*, or *P* values reflecting the difference in symptom change on drug minus symptom change on placebo; or (5) any of the above that could be estimated from manuscript figures. When studies reported multiple outcome measures on the same symptom scale (e.g., symptom change at multiple timepoints following drug administration), outcome measures were averaged together to create a single effect size. If none of these data were reported in a study, we utilized an in-text description of whether the findings were statistically significant, and finally, if none of the above were present, we utilized an in-text description of the direction of effect. Although these latter two sources of data do not permit estimation of a study effect size, they can be used in a formal “vote-counting” procedure (described below).

### Analysis by drug compound

We performed our analysis by combining the effects from all drug trials (naloxone, naltrexone, nalmefene, and buprenorphine) together. Because the bulk of the studies were naloxone trials (*k* = 21), we also performed an analysis of naloxone only trials. Finally, although there were far fewer naltrexone trials (*k* = 6), we also performed a preliminary naltrexone only analysis.

### Subgroup analyses

In order to investigate whether drug effects were strongest at specific post-administration times, we conducted subgroup analyses of all studies reporting data 1 h post administration (*k* = 9) and between 3 and 7 h post administration (*k* = 11). These time periods were chosen because of the relatively large number of studies reporting results at these times, and because we thought them likely to reflect meaningful differences in the acute pharmacokinetics of these compounds.

### Statistical methods (synthesis of results)

Average effect sizes (Hedge’s *g*; a type of standardized mean difference) across all studies were estimated in a standard random effects model [26]. A random effects approach was selected because of substantial heterogeneity across studies in diagnostic instruments, symptom scales, and study compounds, which lead us to presume a priori that studies would exhibit substantial heterogeneity; this was also formally tested with *Q*, and we report *I*^2^ as an estimate of the proportion of between-study variance due to heterogeneity in true effect sizes.

In order to include studies that did not report sufficient information to estimate an effect size, but did report either the presence or absence of a statistically significant (i.e., *P* < 0.05) effect or the direction of an effect (i.e., *P* < 0.5), we also employed maximum-likelihood estimation vote-counting methods for estimating an average effect across all studies [27]. However, we did not use the significance testing framework that accompanies these methods because of concerns that the fixed effects model they depend on is not appropriate for this dataset, which included a range of compounds, diagnostic criteria, symptom scales, and other differences in study design (see ref. [28] for a discussion of the serious issues with fixed effects models). Consequently, as we have done in multiple prior meta-analyses [13, 14], we instead employed a bias-corrected and accelerated (BCa) bootstrap to calculate *P* values and confidence intervals, using 10,000 resamplings of the data with replacement [29] for each analysis. That is, the average effect size estimate obtained from vote-counting methods for each of the 10,000 resampled datasets was calculated, and standard methods were employed to use this distribution of bootstrapped results to obtain valid confidence intervals and *P* values on the observed dataset [29]. It should be noted that although this is a somewhat nonstandard meta-analytic technique, boostrapping is nonetheless a widely employed and generally accepted statistical method, and the random effects meta-analysis described in the preceding paragraph is an entirely traditional approach to meta-analysis. We undertook this additional technique in order to ensure that all available data were included, even if said data could not be incorporated into a traditional meta-analytic framework due to poor reporting practices in older studies.

Both standard and bootstrap models were evaluated with and without study quality weighting (see below). Finally, in order to assess the risk of publication or other bias in the meta-analysis we employed a funnel plot (Supplemental Fig. S1) and the Begg’s rank correlation test for the largest analysis we report on, the analysis of all scales across all compounds. We opted to only perform bias detection on this analysis because it has maximal power to detect any bias, and we have no a priori basis to expect bias in only some analyses.

### Assessment of risk of bias in individual studies

In order to determine whether the methodological quality of studies played a role in biasing the results of individual studies, we assigned a penalty score to each study based on a number of methodological criteria as detailed in the Supplement and Supplementary Tables S4, S5. Authors SC and JML also performed a Cochrane risk of bias assessment of the included studies using the Cochrane Risk of Bias 2 tool (RoB2; [30]). This was done only on studies that met our inclusion criteria and is intended as a supplementary assessment of the risk of bias and not as an exclusion criteria. Details of the analysis are reported in the Supplement and are reported in Supplementary Figs. S17, S18.

### Moderator analyses

For naloxone, we also examined moderator effects of time (minutes post administration), days of administration, opioid antagonist dosage, antipsychotic dosage (chlorpromazine equivalents), and study quality weights (in the unweighted analysis only) using a BCa bootstrap on a weighted regression model (with standard meta-analytic study weights) [26]. Because we investigated five moderators, we employed a Bonferroni-corrected alpha of 0.01 to establish significance.

## Results

### Study selection

The details of our study selection and exclusions with reasons are shown in our PRISMA flow chart (Fig. 1). The results from the database search resulted in 8556 records from mainstream sources, 11 records identified though bibliography and journal search, and 21,500 records from Google Scholar. After duplicates were removed there were 7963 records from conventional sources and 21,500 from Google Scholar. After screening by abstract there were 86 sources remaining from conventional sources and 93 from Google Scholar. These were combined and after duplicates were removed, there were 97 unique records. These were further refined with a predetermined set of exclusion criteria to remove studies that did not contain sufficient data for analysis or did not meet our required criteria for quality.

### Exclusion with reasons

We excluded studies if they contained any of the following criteria: single-patient case reports [31–35], lack of placebo control [36–45], failure to specify direction of effect [38, 46–57], administration of mixed novel therapeutic drugs other than opioid antagonists (not including baseline antipsychotic treatments) [58], change in design midway through the study [59], inclusion of patients with mental illnesses other than schizophrenia, schizoaffective disorder, and schizophreniform disorder, and lack of stratification by diagnosis [60, 61] (however, if a study did include patients with other mental illnesses but published the individual patient level data, we included the data only from patients who only carried a diagnosis of schizophrenia, schizoaffective disorder, schizophreniform disorder, and excluded data from the patients with other diagnoses), and no English translation available for the full text [62]. Finally, if identical results from a single trial were published twice in two separate journals, we included only the results from the publication with the larger dataset in our analysis. If multiple publications contained the exact same data, then we included only the earlier publication, i.e., citations [43, 47, 56, 63–86]. The characteristics of excluded studies are reported in Supplementary Tables S8, S9.

### Study characteristics

This resulted in 27 publications detailing 30 blinded placebo-controlled trials for our final analysis comprising 434 total patients. Of these 30 trials, one utilized nalmefene [87] and one utilized buprenorphine [88], resulting in 28 trials that utilized naloxone or naltrexone [8, 22, 64–67, 70–72, 84, 86, 89–102]. The characteristics of included participants are reported in Supplementary Table S6. The full study characteristics (PICOS) of included studies are reported in the Supplement and in Supplementary Table S7.

### Publication and other bias assessment

Visual examination of a funnel plot of the primary analysis (all scales, all compounds) revealed no evidence of bias in this literature (Supplementary Fig. S1), nor did the formal Begg’s rank correlation test (*r* = 0.03; *P* = 0.87). However, the Cochrane RoB2 analysis found that 10 of the 27 publications were at high risk of bias, 16 of the publications were at some concern of bias, and 1 of the publications was at low risk of bias (Supplementary Figs. S17, S18).

### Primary analyses of all compounds

**Fig. 2:**
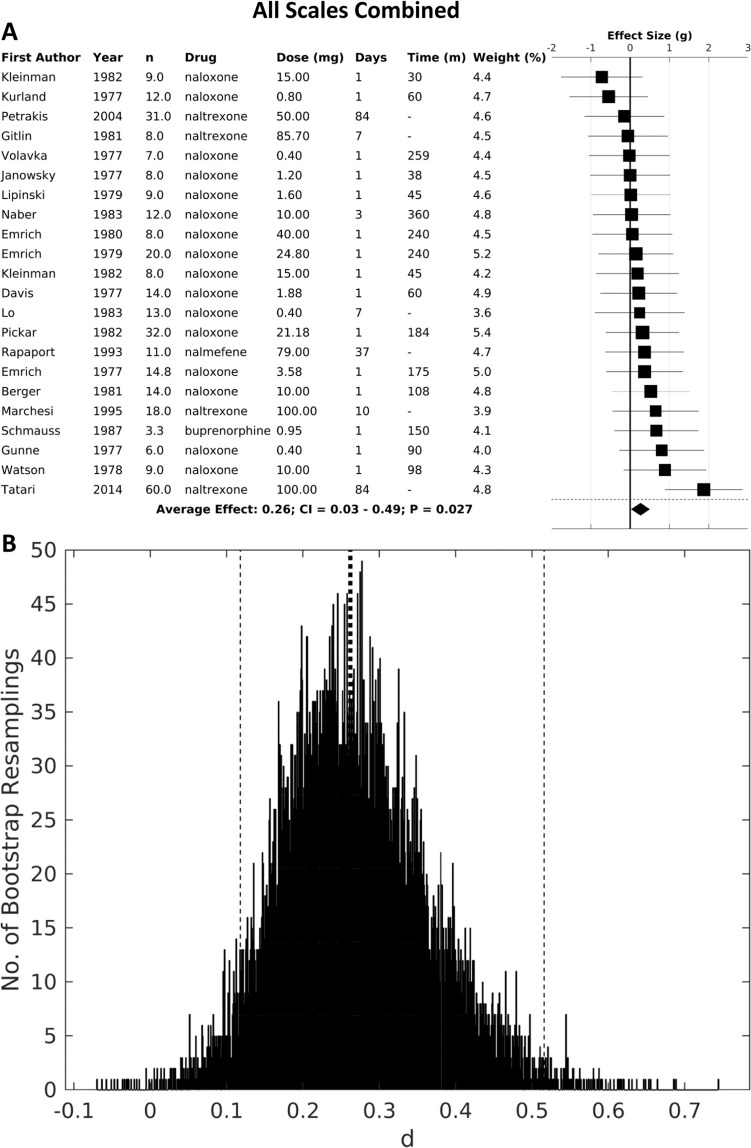
All drugs combined on all scales combined unweighted. **a** Forest plot of all drugs combined on all scales combined. When studies reported multiple effects that met inclusion criteria (see Supplement), study *n* is reported here as the harmonic mean of all included effects (potentially resulting in fractional values of *n*). Dose and time were reported as the mean of all included effects. Weight (%) is the normalized weight of each study, which is also proportional to the area of the box shown for each study in the forest. The whiskers for each study plot show the 95% confidence interval for that study. The diamond displayed at the bottom of the plot is centered on the average effect size, with the width of the diamond demonstrating the 95% confidence interval on the average effect. **b** Histogram of bootstrap distribution of the average effect size for the analysis of all drugs combined on all scales combined. The thick dotted line shows the observed average effect size, while the thin dotted lines show the 95% confidence interval on the average effect size.

Results from all analyses on the effect of all drugs (naloxone, naltrexone, nalmefene, and buprenorphine) on each subset of symptom scales are presented in Table 2 (missing entries in the table reflect analyses that were not conducted due to insufficient studies reporting relevant data). Significant decreases in symptoms following treatment with opioid antagonists were observed for all analyses (weighted and unweighted, and both standard and bootstrap models) that included all symptom scales Fig. 2 (see Table 1 for details of included scales), as well as all positive symptom scales, thus demonstrating an effect of these compounds on positive symptoms. Analyses of total scales (which include negative as well as general symptom measures; see Table 1) showed significant effects only in the bootstrap models, with the random effects model having both a smaller effect size and smaller number of included studies, and thus failing to achieve significance. Analyses of negative scales were severely underpowered (*k* = 6 for bootstrap only, random model not assessed) and did not achieve significance, but did have a fairly large average effect size (*g* > 0.66). Analyses of scales measuring only a single symptom, hallucinations or delusions, showed significant results in bootstrap models but only a trend level effect for random effects models of hallucinations (separate delusion scales were not reported in a sufficient number of studies for analysis in a standard model). Finally, an analysis of only total positive symptom scales (e.g., SAPS Total or PANSS Positive scores) also showed a significant result in bootstrap models with only marginal trend effects in standard random effects models. The full results of both weighted and unweighted analyses are included in Supplementary Figs. S2–S8 and Supplementary Tables S10, S11.

**Table 2:** Meta-analysis results for all drugs combined.

Weighting	Model	*G*	*P*	*k*	CI	*I*^2^	*P* (*I*^2^)
All Scales							
Unweighted	Standard	0.263	0.0266	22	0.034–0.493	0.799	<0.0001
	Bootstrap	0.262	0.0002	30	0.118–0.516	–	–
Weighted	Standard	0.288	0.0266	22	0.034–0.493	0.799	<0.0001
	Bootstrap	0.283	<0.0001	30	0.140–0.602	–	–
Total Scales							
Unweighted	Standard	0.135	0.1198	14	−0.040–0.311	0.421	0.0490
	Bootstrap	0.193	0.0155	19	0.0468–0.351	–	–
Weighted	Standard	0.135	0.1198	14	−0.040–0.311	0.421	0.0490
	Bootstrap	0.192	0.0197	19	0.0361–0.356	–	–
All Positive Scales Combined							
Unweighted	Standard	0.338	0.0158	17	0.073–0.604	0.813	<0.0001
	Bootstrap	0.328	<0.0001	21	0.159–0.656	–	–
Weighted	Standard	0.373	0.0158	17	0.073–0.604	0.814	<0.0001
	Bootstrap	0.362	<0.0001	21	0.192–0.782	–	–
All Negative Scales Combined							
Unweighted	Standard	–	–	–	–	–	–
	Bootstrap	0.667	0.1388	6	−0.185–2.217	–	–
Weighted	Standard	–	–	–	–	–	–
	Bootstrap	0.711	0.1185	6	−0.124–2.217	–	–
Hallucinations							
Unweighted	Standard	0.468	0.0934	9	−0.099–1.035	0.918	<0.0001
	Bootstrap	0.391	0.0091	14	0.098–0.985	–	–
Weighted	Standard	0.574	0.0934	9	−0.099–1.035	0.920	<0.0001
	Bootstrap	0.454	0.0031	14	0.134–1.163	–	–
Delusions							
Unweighted	Standard	–	–	–	–	–	–
	Bootstrap	0.728	<0.0001	7	0.169–2.031	–	–
Weighted	Standard	–	–	–	–	–	–
	Bootstrap	0.844	<0.0001	7	0.214–2.031	–	–
Total Positive Scales							
Unweighted	Standard	0.442	0.0840	8	−0.077–0.962	0.879	<0.0001
	Bootstrap	0.442	<0.0001	8	0.142–1.195	–	–
Weighted	Standard	0.469	0.0840	8	−0.077–0.962	0.949	<0.0001
	Bootstrap	0.469	<0.0001	8	0.138–1.384	–	–

As anticipated, formal tests for heterogeneity were significant, and observed *I*^2^ values were quite large, with all values from 80% to 95% other than those for total scales (42%; see Table 2). This was anticipated due to the considerable variability across studies in patient age, chronicity, medication status, diagnostic criteria used, and symptom outcome measures employed. This high level of heterogeneity indicates that some of the patient samples, symptom measures, or even compounds included in our analysis could potentially have very small or even nonexistent true effects. Nonetheless, the random effects methods we employed here are appropriate methods in the presence of heterogeneity, and demonstrate that the overall population of studies included in our analyses show significant effects. Thus, while the heterogeneity we observe demands some degree of caution in interpreting results, the available evidence points to a therapeutic benefit of opioid antagonists for patients with schizophrenia, which should be followed up with modern, carefully controlled, randomized clinical trials.

### Subgroup analyses

We found numerically smaller effects (*G* = 0.19) with naloxone alone in the bootstrap models (*k* = 22), which did not reach significance in the standard random effects analysis, which had less power (*k* = 16). We did not find any significant effects with naltrexone alone, which suffered from very low power (*k* = 6). The full results of these analyses are reported in the Supplement, Supplementary Figs. S9–S16, and Supplementary Tables S10, S11. Analyses of posttreatment timepoints at 1 h and between 3 and 7 h for all drugs combined did not demonstrate any clear differences in effects at these timepoints, which also suffered from low power due to the small number of studies reporting data that could be included in these analyses (*k* from 6 to 11), and are detailed in the Supplement and Supplementary Tables S12, S13.

### Moderator analyses

**Fig. 3:**
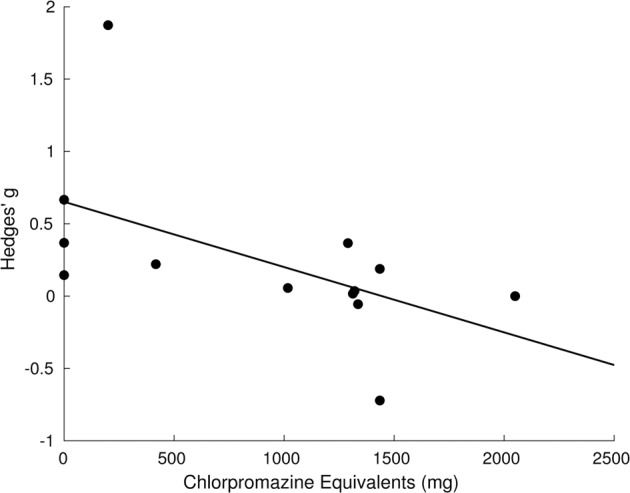
All drugs all symptoms chlorpromazine regression. Scatterplot of the relationship between chlorpromazine-equivalent dosage of D2-receptor antagonist compounds and the magnitude of the effect of opioid antagonists. Solid line shows the estimated relationship from the meta-regression.

Of the variables included in moderator analyses (see Methods), only antipsychotic dosage (chlorpromazine equivalents) was significant in the analysis of all symptoms (naloxone only analysis; *β* = −0.00045; *P* = 0.0001;*k* = 13; CI = −0.00133 to −0.00014), indicating a weaker effect of opioid antagonists on patient samples treated with higher doses of traditional antipsychotic medications. This relationship is shown in Fig. 3 and the full results are reported in Supplementary Table S14.

## Discussion

This meta-analysis of 434 patients with schizophrenia provides the best evidence to date, and the only meta-analytic evidence, of an effect of opioid antagonists in treating the symptoms of schizophrenia. In addition to an overall effect on symptoms using the broadest symptom measure available in each study and an overall effect on positive symptom scales, we found significant effects in our bootstrap models of opioid antagonists on both hallucinations and delusions, suggesting antipsychotic activity for a class of compounds that are not dopamine 2 receptor (D2R) antagonists. Moreover, in 22 of the 30 trials, patients were stabilized on D2R antagonists (on average, 773 mg chlorpromazine equivalents), suggesting a potential clinical application of opioid antagonists may be as an adjunctive treatment to standard antipsychotic therapy, and that opioid antagonists may be useful in treatment-resistant patients. However, our moderator analysis of 13 trials with chlorpromazine-equivalent dose information suggested that the effect of opioid antagonists is diminished in an adjunctive setting, at least at higher doses of D2R antagonists. In addition, it was not possible for us to separately assess the effects of opioids on thought disorder symptoms, as too few studies reported them as an independent endpoint. Although analyses of positive symptoms overall revealed significant effects, we cannot rule out that these effects were driven entirely by hallucinations and delusions. Careful, high-quality randomized controlled trials with larger samples and a full range of symptom endpoints will be needed in order to determine the clinical utility of opioid antagonists as adjunctive or monotherapies for the symptoms of schizophrenia.

Of note, observed effect sizes for positive symptoms were of similar magnitude to those in clinical trials of traditional antipsychotic monotherapies, even though a majority of studies used opioid antagonists as an adjunctive treatment. This suggests that opioid antagonists could potentially be as effective a treatment as D2R antagonists, and that this benefit may be additive with standard treatments. Although our analysis of negative symptoms was underpowered and did not reach significance, the effect sizes were quite large at *g* = 0.66 or larger, raising the possibility of potential for efficacy on negative symptoms. However, a major limitation of our negative symptom data is that trials in our analysis were not designed to differentiate primary from secondary negative symptoms using modern approaches, and thus the observed improvement in negative symptoms may have been secondary to improvement in positive symptoms. This strongly reinforces the need for further trials specifically designed to test efficacy on primary negative symptoms. Finally, we provide a brief discussion of potential therapeutic mechanisms of opioid antagonists in the Supplement.

### Limitations

There are several limitations to this study. First, while hypotheses were generated before the study began, we did not pre-register a review protocol. In addition, the included patient populations were heterogeneous with ages ranging from 18 to 78, and there is substantial missing/unknown patient information (Table 2), thereby limiting determination of the specific patient populations that opioid antagonists may benefit. Moreover, while all included studies specified that patients receiving opioid antagonists were either medication-free or were on a stable medication dose before each study began, it is not possible to know the exact number of patients receiving antipsychotics, as some studies included patients both on and off antipsychotics and combined the data. However, our moderator analysis of D2R antagonist dose indicated that effect sizes should be larger than those reported here in patients who are otherwise medication-free.

Another potential source of bias is the selection of patients within each trial. To maximize power, as our sample of 434 patients is relatively low, it was necessary to include older studies that used DSM-II diagnostic criteria and studies that did not clearly specify randomization, thus potentially including patients that would not be diagnosed with schizophrenia under newer DSM criteria. This concern is mitigated, however, by the absence of a relationship between effect size and study quality, suggesting that these considerations did not have an undue impact on effects reported here. Finally, while we included all studies meeting our predetermined inclusion and exclusion criteria and utilized the Cochrane RoB2 only as a supplement for completeness, results of the RoB2 analysis show that 10 included studies are at high risk of bias and 16 have some concern. However, the RoB2 is designed for assessing modern studies, and for older trials the standard of reporting was not as rigorous as would be expected today. In most of these cases, missing information in older trials would automatically flag the trial as some concern or high risk. For example, studies published in the 1970s cannot be expected to report the method used for randomization and allocation sequence, or to publish a prespecified statistical analysis plan.

## Conclusions

Although these findings remain preliminary due to the limited number of available studies, these results provide a strong rationale for a systematic effort through larger double blind randomized controlled trials to resolve the potential efficacy of opioid antagonists either as monotherapy compared with placebo or as adjunctive treatment with standard of care antipsychotics vs. antipsychotics alone for both the positive and negative symptoms of schizophrenia and, if effective, the optimal dosage and regimen of these compounds.

If these findings are confirmed with larger randomized trials, the use of opioid antagonists in schizophrenia could represent a paradigm shift in the management of this patient population. Since opioid antagonists are already available generically, their implementation in the clinic could be relatively simple and present the potential for a major public health impact.

## Funding and disclosures

SDC reports he receives compensation from employment in Terran Biosciences and has stock ownership in Terran Biosciences. JXVS was supported by K01 MH 107763 and reports no conflict of interest. JML reports no conflict of interest. AA-D reports having received stock options in Terran Biosciences.

## Supplementary information

Supplemental material
