# Supplementary material for: Opioid antagonists are associated with a reduction in the symptoms of schizophrenia: a meta-analysis of controlled trials
Source: Neuropsychopharmacology. 2020 Jun 9;45(11):1860–9. doi: 10.1038/s41386-020-0730-z (PMC7608351; doi:10.1038/s41386-020-0730-z)
Supplement: Supplementary file 1 — Supplemental material [file 41386_2020_730_MOESM1_ESM.pdf]

Supplemental Information

Supplemental Methods

Information sources and study selection: full details

The bibliographies from the searches were input into Endnote and duplicates were removed. The Google Scholar search was performed separately. Study selection was performed by author SC by initially reading the abstract. This resulted in 86 full text articles from mainstream databases and 93 articles from Google Scholar. These were then combined into Endnote and Duplicate results from these searches were again removed with Endnote. This identified 97 articles which were then read by SC in full to determine if they met the inclusion criteria. In cases of ambiguity JXVS was consulted. In cases of disagreement it was determined a third author AAD would be consulted, however there were no disagreements. A predetermined set of exclusion criteria (determined by SC and JXVS before the start of the study) was then applied by SC. In cases of ambiguity SC and JXVS reviewed the full text together and compared to the exclusion criteria. It was predetermined that in cases of disagreement a third author AAD would be consulted, however there were no disagreements. The details of the exclusion criteria are below in the **Supplemental Methods**. This procedure identified 27 English language publications of clinical trials where naloxone, or naltrexone, or nalmefene, or buprenorphine was administered to patients with schizophrenia and an effect on the negative or positive symptoms was recorded. Unfortunately, the long amount of time that had passed from the time many of these trials were conducted (~40 years) made contacting the study authors impractical, and we did not attempt to do so.

Data collection process: full details

The full text of each manuscript was examined by author SC for reported results contained in the figures, tables, and text. Preference was given for numerical scores reported in tables and text, however if only a figure with a scale was present, than numerical scores were extracted from the figure by making a digital scan of the figure and then counting individual pixels in

Adobe Photoshop to calculate the numerical scores. For each study SC extracted the data relating to the change in outcome scale on drug and on placebo and input it into an Excel file with double entry. In cases of ambiguity over whether specific results of statistical results in the manuscripts were able to be used (e.g., whether appropriate degrees of freedom were utilized), JXVS was consulted and the manuscript was considered by SC and JXVS together in full. Finally, if no numerical data was present but the authors unambiguously indicated the direction of effect then the direction only was input into the Excel file. Author JL checked the data for consistency against the original manuscripts and it was determined JXVS would be consulted in cases of disagreement. The timepoint where each change was measured and other information such as dose, method of administration, age, years of illness, and chlorpromazine equivalents is reported in **Supplemental Tables S6-S7**. We used only the data contained within each manuscript and did not contact study authors (see above).

Data items and calculation of effect sizes additional details

Because most included studies employed a crossover design, in order to calculate effect sizes we had to estimate the within-condition SD from the SD of the difference scores, using established methods [1]. This estimate requires the correlation between scores in each condition, which we estimated from a subset of 9 studies that reported the necessary individual-subject level data (**Supplemental Table S3**). The mean correlation coefficient across these 9 studies was assumed to be the true correlation between measures for all studies for the purpose of this calculation. While seven of the studies specified that they utilized an AB vs BA design, most of the studies did not specify this information explicitly; in order to have sufficient power to complete our analysis, we had to include both arms into our analysis.

Assessment of Risk of Bias (Study Quality) Penalty Scores

First, studies were penalized based on the reliability of the schizophrenia diagnostic criteria used, with a greater penalty for less reliable criteria shown in **Supplemental Table S4**. Studies were also assigned a penalty based on the reliability of the clinical scale used,

determined by a review of literature on the clinical scales as detailed in the **Supplemental Methods** and **Supplemental Table S5.** In order to avoid potential bias of subjective penalty scores on the outcome of our study, we performed all analyses both with and without penalty weights. To the extent that meta-analytic results differ between weighted and unweighted analyses, there may be some concern that results were impacted by biases inherent to individual studies of poor quality.

The diagnostic criteria utilized in the included trials included Diagnostic and Statistical Manual of Mental Disorders 2nd edition DSM-II [2], International Classification of Disease ninth edition (ICD-9), Feighner's criteria [3], Research Diagnostic Criteria (RDC) [4], Diagnostic and Statistical Manual of Mental Disorders 3nd Edition (DSM-III) [5] , Diagnostic and Statistical Manual of Mental Disorders 3nd Edition Revised (DSM-III-R) [6], Diagnostic and Statistical Manual of Mental Disorders 4th edition (DSM-IV) [7], and Diagnostic and Statistical Manual of Mental Disorders 4th edition Text Revision (DSM IV-TR) [8]. Older criteria were assigned greater penalty scores than more recent diagnostic criteria.

More reliable scales were determined based on published literature evaluating their ability to reliably rate the symptoms of schizophrenia across multiple studies. We grouped these studies into five groups, see **Table 1**. BPRS, PANSS, SAPS, and SANS are among the most extensively utilized in clinical trials and are considered to be highly reliable [9-12] and received no penalty (score 0). In the second category we assigned a penalty score of 0.1 to IMPS [13] and the NIMH scale [14]. In the third category we assigned a penalty score of 0.2 to CPRS [15]. In the fourth category, we assigned a penalty score of 0.3 to VBS [16-19]. Finally, we assigned a penalty of 0.4 to studies that reported "self-rated" or "custom" scales that were unique to that research group.

Penalty scores were summed across sources (diagnostic criteria and clinical scale used), and a weight, w_k for each study was calculated as $w_k = \frac{1}{2+P_k}$, where P_k is the penalty

score of the kth study. In order to avoid potential bias of subjective penalty scores on the outcome of our study, we performed all analyses both with and without penalty weights. To the extent that meta-analytic results differ between weighted and unweighted analyses, there may be some concern that results were impacted by biases inherent to individual studies of poor quality.

Assessment of Risk of Bias in individual studies using the Cochrane Risk of Bias 2 tool

Although our meta-analysis is not a Cochrane review, we used the Cochrane tool for assessing risk of bias as a supplementary method to assess the risk of bias[20]. Two authors SC and JL used the Cochrane tool to assess the risk of bias in each study in the following domains: randomisation process, deviations from intended interventions, missing outcome data, measurement of the outcome, selection of the reported result, and overall bias. Before beginning our analysis, we pre-specified that in cases of disagreement a third author JVS would be consulted. Following the Cochrane Handbook for Systematic Reviews of Interventions guidelines, we scored each of the domains as “high”, “low”, or “some concerns.” We utilized the algorithms provided by the RoB2 tool and did not override any of the pre-specified algorithms. Since the Cochrane assessment of risk of bias was not a pre-specified exclusion criteria, we performed this assessment only on the studies that already met our inclusion criteria and did not use the results to make any inclusions or exclusions of the studies utilized in our analysis. Additionally, due to the high number of studies that met the criteria for “high” or “some concerns” we were not able to do a post study subgroup analysis with only the studies of low bias as Tatari et al. 2014 was the only study that was rated as low bias overall. Two limitations of our use of the tool are that many of the studies included in our analysis are >40 years old and lack the same detail of reporting as modern studies. In this case lack of information increases the risk of bias. For all trials except Tatari et al., 2014, we were only able to complete the assessment with the primary journal article and did not have access to trial protocols or a statistical analysis plan. Additionally, the tool is better suited for two arm trials than crossover trials and the majority of

the studies in our analysis (25/30) are crossover trials. In the case of crossover trials, we also took into account the Cochrane recommendations for assessing bias in cross-over trials [21] including: appropriate use of cross-over design, random order of treatments received, and potential bias from carry-over effects, reporting of dropouts. The domain level results are presented in **Supplemental Figure S17-S18** in the supplement to help readers interpret the potential for bias in the included studies and in the results of our meta-analysis as a whole.

Supplemental Results

Characteristics of included studies

Of the included trials, 27 were double blinded and three were single blinded, 25 were placebo controlled crossover trials, and 5 were double blinded placebo controlled two arm trials. Of note, 22 of the trials included patients on antipsychotics, or mixed patients on and off antipsychotics. For the 10 trials that specified chlorpromazine equivalents (n = 151 patients), the average equivalent per patient was 773 mg. full details in supplemental figure s6

Additional Subgroup analyses

There was no clear differences in the effect size at one hour and at three to seven hours. At one hour, we found an effect of all drugs combined on all scales combined only in the weighted bootstrap model (g = .21, P = .02, k = 9, CI = 0.03 - 0.065) only. At three-seven hours, we found an effect of all drugs combined on all scales combined in the weighted and unweighted bootstrap models (unweighted shown here: g = .23, P = .01, K = 11, CI = 0.04 - 0.43). We also found an effect on total scales in the weighted bootstrap model only (g = .23, P = .01, k = 10, CI = 0.05 - 0.52).

Additional Naloxone only analysis

As with the overall analysis reported in the main manuscript, when we stratified our analysis to only include studies with naloxone, we found an effect on all symptom scales combined in the weighted model bootstrap model only (g = 0.2219; P = 0.005019; k = 2122, CI = 0.03 - 0.35) (**Supplemental Figure S7A-S9A-B**). In the unweighted model, we did not find a significant

effect with the bootstrap effects model (g = 0.19; P = 0.0603; k = 2122, CI = 0.016 - 0.34) or the random effects model (g = 0.1715; P = 0.1311; k = 1516). We found an effect of naloxone on total scales weighted bootstrap model only (g = 0.20; P = 0.03; k = 11, CI = -0.04 - 0.34) (**Supplemental Figure S3C-D10A-B**).

When we considered all positive symptoms, we found an effect on all positive scales combined inn the combined random effects model (g = 0.2623; P = 0.04 03 k = 12, CI = 0.01 - 0.44) and bootstrap effects model (g = 0.2524; P = 0.04 02 k = 16, CI = 0.03 - 0.40) (Supplemental Figure 3E-F11A-B). For hallucination subscales, we found an effect only in the weighted bootstrap effects model (g = 0.2926; P = 0.0203; k = 13, CI = 0.03 - 0.49) (**Supplemental Figure S8A-B-S12A-B**), but the effect for delusion subscales was significant in both bootstrap models (unweighted shown here: g = 0.38; P = 0.0001; k = 6, CI = 0 - 1.00), however there were not enough studies for the random-effects model. There were not enough studies to analyze naloxone on any other scales.

Additional Naltrexone only analysis

Due to the small number of naltrexone studies we were only able to perform preliminary analysis on all symptoms with the bootstrap model. We did not find a significant effect of naltrexone in the weighted or unweighted analysis (g = 0.58; P = 0.2021; k = 6, CI = -0.11 - 1.87), although the observed effect size was relatively large. There were not enough studies to analyze any other parameters.

Supplemental Discussion

Potential mechanism

In regard to a potential therapeutic mechanism, we propose that the potential efficacy of these opioid antagonists is most likely dependent on their ability to antagonize the kappa opioid receptor, shared by all drugs tested here, rather than the mu or delta opioid receptor. The kappa opioid receptor has been shown to modulate psychotic states in healthy volunteers and clinical studies in humans with a variety of selective kappa agonists have shown psychotomimetic

effects that have similarities to the primary symptoms of patients with schizophrenia [22-32]. Similarly the psychotomimetic effects of selective kappa agonists in healthy volunteers can be blocked by naloxone [22, 32, 33] and naltrexone [29].

If, indeed, the therapeutic effect of pan opioid antagonists is mediated through antagonizing the kappa opioid receptor, a selective kappa opioid receptor antagonist may have additional therapeutic benefit beyond that of non-selective pan-opioid antagonists. However, there are not currently any FDA approved selective kappa opioid antagonists. Our analysis suggests the need for further research into both a potential role for the opioid system, specifically the kappa opioid receptor, in the pathogenesis of the positive and negative symptoms of schizophrenia. However, it is impossible to rule out involvement of the mu receptor as a recent positron emission tomography study showed potential involvement of the mu receptor in schizophrenia [34]. While the mu receptor has been implicated in analgesia, reward, and drug abuse [35, 36] it, with the delta receptor plays an important role in dopamine modulation in the nucleus accumbens, an area that has been implicated in the positive symptoms of schizophrenia [37].

Choice of Opioid Antagonist for Future trials

Finally, there is a lack of long-term studies of opioid antagonists in schizophrenia, and studies seeking to examine an adjunctive effect on negative symptoms may require a longer timeframe [38-40]. Unfortunately, naloxone is not suitable for long-term administration because it must be injected, and has a very short half-life of 60-90 minutes [41]. Of the currently available opioid antagonists, naltrexone and nalmefene are most suitable for long-term administration. Naltrexone and nalmefene are both orally active with the longer half-lives of 10.3 hours [42] and 11 hours [41] respectively, and thus have the advantage of being able to be given chronically, making them suitable for use in future clinical trials examining the long term benefit of opioid antagonists in schizophrenia. Unfortunately, buprenorphine has potential for abuse [43] and

dependence [44] due to its activity as a mu partial agonist, making it less ideal for widespread use.

Supplemental Figures:

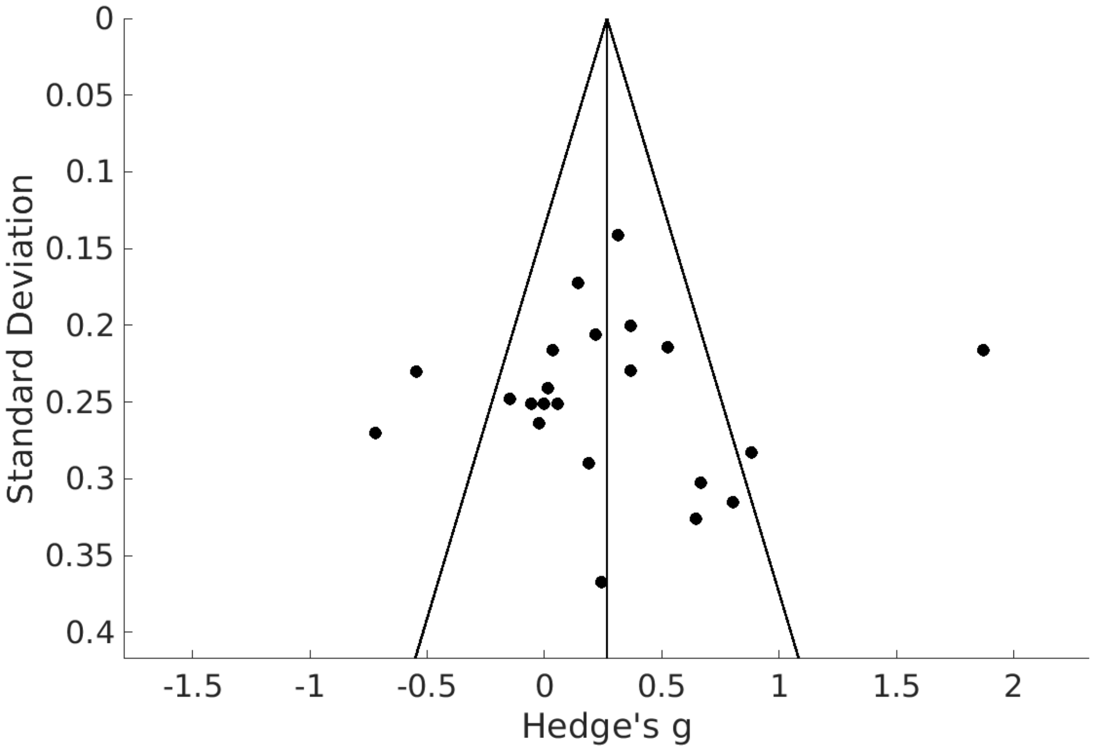

Supplemental Figure S1. Funnel plot for the primary analysis (All Symptoms; k = 22).

Total Scales

A

First Author	Year	n	Drug	Dose (mg)	Days	Time (m)	Weight (%)
Kleinman	1982	9.0	naloxone	15.00	1	30	6.1
Petrakis	2004	31.0	naltrexone	50.00	84	-	6.8
Gitlin	1981	8.0	naltrexone	85.70	7	-	6.7
Volavka	1977	7.0	naloxone	0.40	1	259	6.3
Lipinski	1979	9.0	naloxone	1.60	1	45	7.1
Naber	1983	12.0	naloxone	10.00	3	360	8.1
Emrich	1980	8.0	naloxone	40.00	1	240	6.7
Emrich	1979	20.0	naloxone	24.80	1	240	10.2
Lo	1983	13.0	naloxone	0.40	7	-	3.9
Pickar	1982	32.0	naloxone	21.18	1	184	12.0
Rapaport	1993	11.0	nalmefene	79.00	37	-	7.5
Emrich	1977	14.8	naloxone	3.58	1	175	8.8
Marchesi	1995	18.0	naltrexone	100.00	10	-	4.7
Schmauss	1987	3.3	buprenorphine	0.95	1	150	5.2

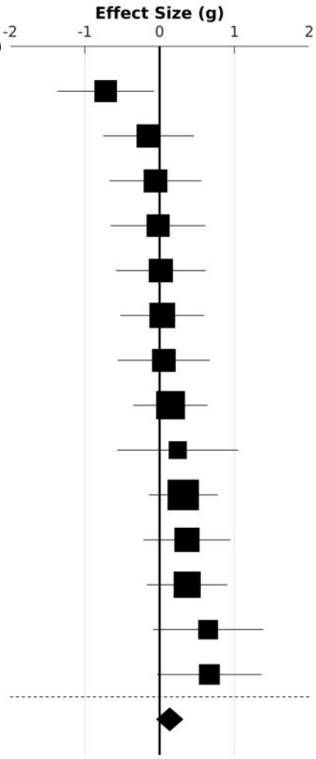

Average Effect: 0.14; CI = -0.04 - 0.31; P = 0.120

B

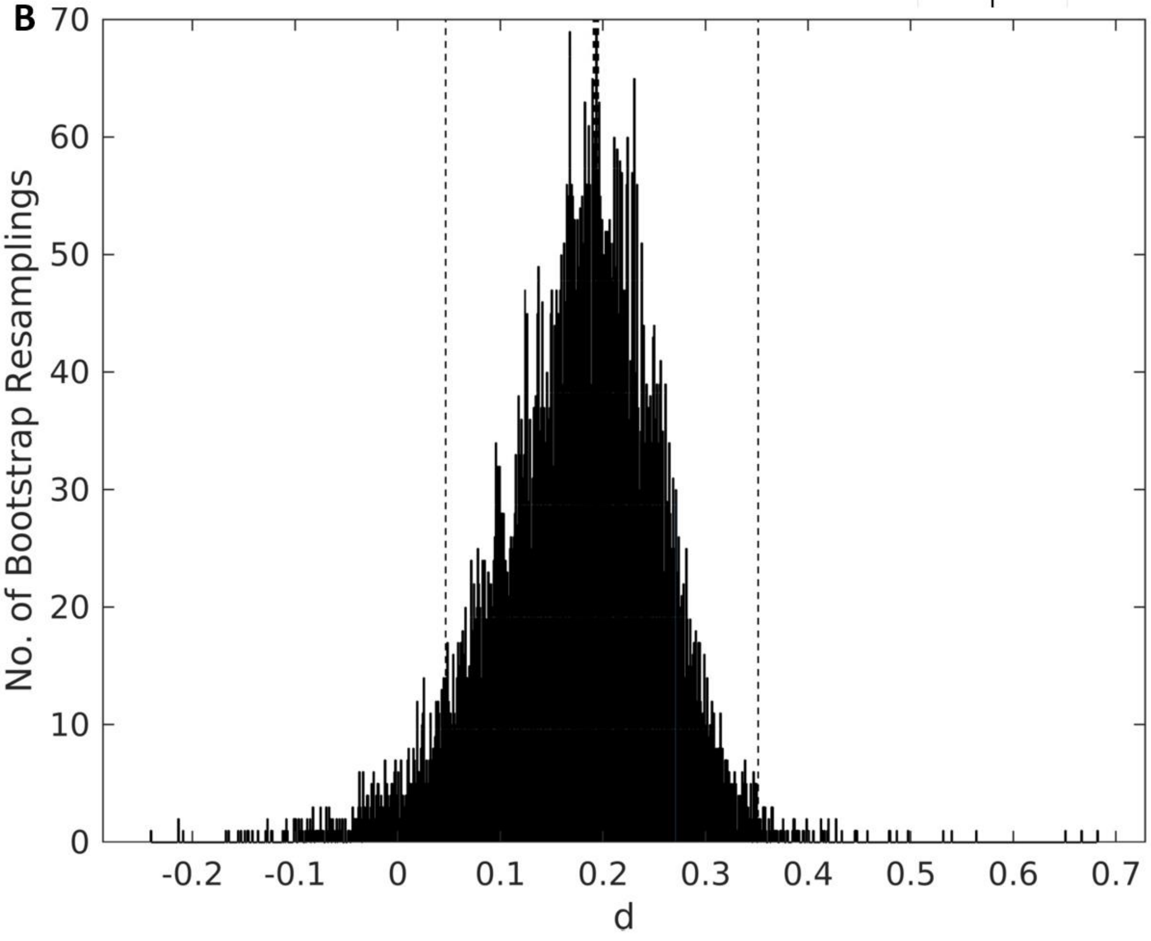

Supplemental Figure S2. All drugs combined on Total scales unweighted. A) Forest plot analysis of all drugs combined on total scales. When studies reported multiple effects that met inclusion criteria (see Supplemental Methods), study *n* is reported here as the harmonic mean of all included effects (potentially resulting in fractional values of *n*). Dose and Time were reported as the mean of all included effects. Weight (%) is the normalized weight of each study, which is also proportional to the area of the box shown for each study in the forest. The whiskers for each study plot show the 95% confidence interval for that study. The diamond displayed at the bottom of the plot is centered on the average effect size, with the width of the diamond demonstrating the 95% confidence interval on the average effect. B) Histogram of bootstrap distribution of the average effect size for the analysis of all drugs combined on total scales

All Positive Scales Combined

A

First Author	Year	n	Drug	Dose (mg)	Days	Time (m)	Weight (%)
Kurland	1977	12.0	naloxone	0.80	1	60	6.0
Petrakis	2004	31.0	naltrexone	50.00	84	-	5.8
Volavka	1977	7.0	naloxone	0.40	1	231	5.6
Emrich	1979	20.0	naloxone	24.80	1	240	6.6
Janowsky	1977	8.0	naloxone	1.20	1	38	5.8
Marchesi	1995	18.0	naltrexone	100.00	10	-	5.1
Kleinman	1982	8.0	naloxone	15.00	1	45	5.4
Davis	1977	14.0	naloxone	1.88	1	60	6.3
Pickar	1982	32.0	naloxone	21.18	1	120	6.9
Rapaport	1993	11.0	nalmefene	79.00	37	-	6.1
Emrich	1977	19.2	naloxone	3.58	1	178	6.5
Emrich	1980	8.0	naloxone	40.00	1	120	5.7
Berger	1981	14.0	naloxone	10.00	1	108	6.2
Schmauss	1987	3.3	buprenorphine	0.95	1	150	5.3
Gunne	1977	6.0	naloxone	0.40	1	90	5.1
Watson	1978	9.0	naloxone	10.00	1	98	5.5
Tatari	2014	60.0	naltrexone	100.00	84	-	6.2

Average Effect: 0.34; CI = 0.07 - 0.60; P = 0.016

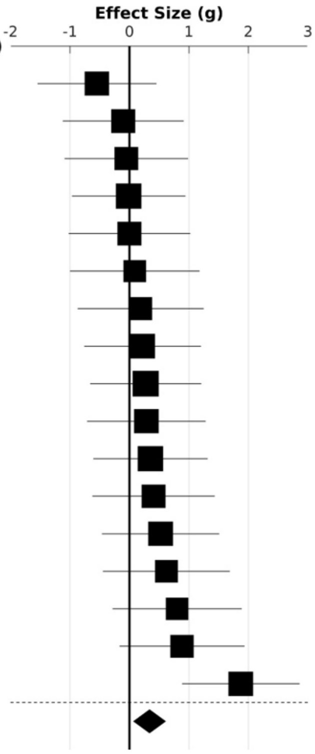

B

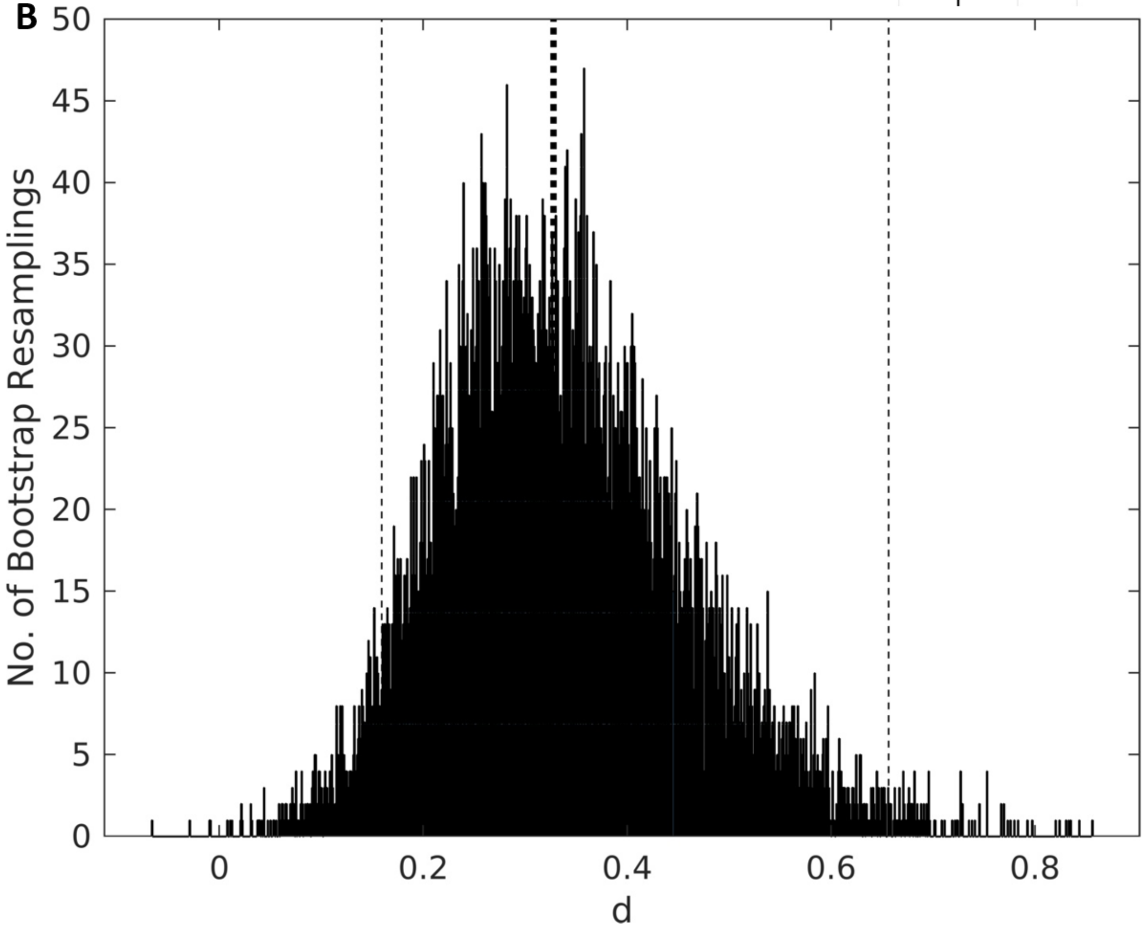

Supplemental Figure S3. All drugs combined on all positive scales unweighted. A) Forest plot analysis of all drugs combined on all positive scales combined. When studies reported multiple effects that met inclusion criteria (see Supplemental Methods), study *n* is reported here as the harmonic mean of all included effects (potentially resulting in fractional values of *n*). Dose and Time were reported as the mean of all included effects. Weight (%) is the normalized weight of each study, which is also proportional to the area of the box shown for each study in the forest. The whiskers for each study plot show the 95% confidence interval for that study. The diamond displayed at the bottom of the plot is centered on the average effect size, with the width of the diamond demonstrating the 95% confidence interval on the average effect. B) Histogram of bootstrap distribution of the average effect size for the analysis of all drugs combined on all positive scales combined.

Hallucination Scales

A

First Author	Year	n	Drug	Dose (mg)	Days	Time (m)	Weight (%)
Kurland	1977	12.0	naloxone	0.80	1	60	11.2
Volavka	1977	7.0	naloxone	0.40	1	259	10.8
Janowsky	1977	8.0	naloxone	1.20	1	38	11.0
Davis	1977	14.0	naloxone	1.88	1	60	11.5
Pickar	1982	32.0	naloxone	21.18	1	120	11.9
Berger	1981	14.0	naloxone	10.00	1	108	11.4
Gunne	1977	6.0	naloxone	0.40	1	90	10.3
Watson	1978	9.0	naloxone	10.00	1	98	10.7
Tatari	2014	60.0	naltrexone	100.00	84	-	11.2

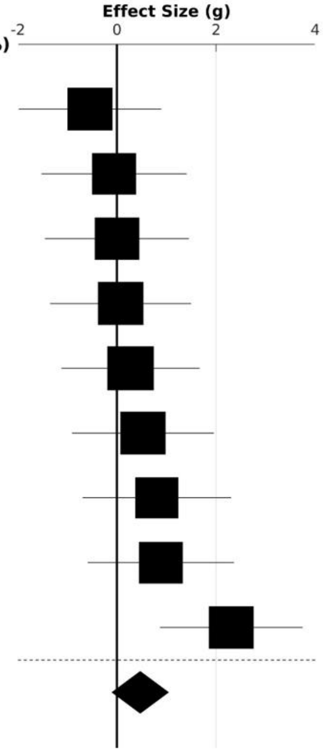

Average Effect: 0.47; CI = -0.10 - 1.04; P = 0.093

B

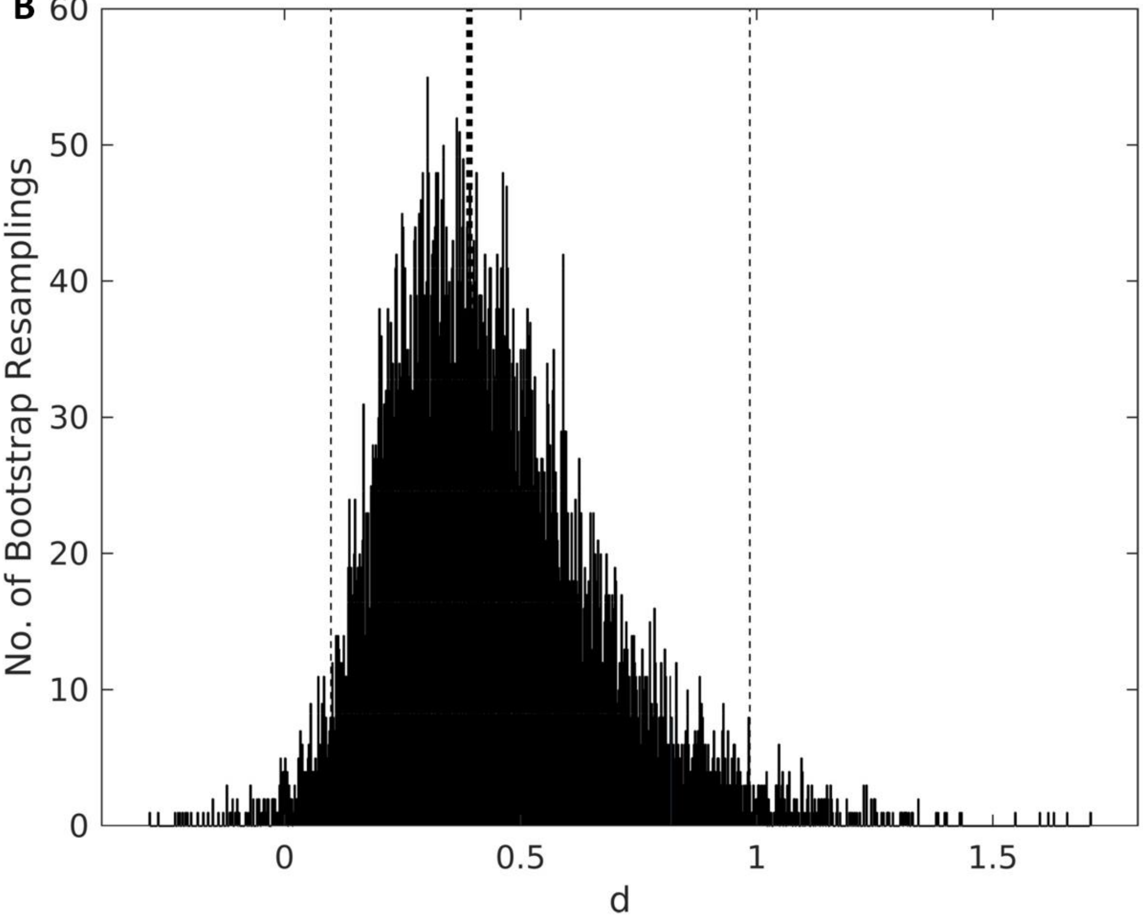

Supplemental Figure S4. All drugs combined on all hallucination scales unweighted. A) Forest plot analysis of all drugs combined on hallucination subscales. When studies reported multiple effects that met inclusion criteria (see Supplemental Methods), study *n* is reported here as the harmonic mean of all included effects (potentially resulting in fractional values of *n*). Dose and Time were reported as the mean of all included effects. Weight (%) is the normalized weight of each study, which is also proportional to the area of the box shown for each study in the forest. The whiskers for each study plot show the 95% confidence interval for that study. The diamond displayed at the bottom of the plot is centered on the average effect size, with the width of the diamond demonstrating the 95% confidence interval on the average effect. B) Histogram of bootstrap distribution of the average effect size for the analysis of all drugs combined on hallucination sub scales.

All Scales Combined

A

First Author	Year	n	Drug	Dose (mg)	Days	Time (m)	Weight
Kleinman	1982	9.0	naloxone	15.00	1	30	4.4
Kurland	1977	12.0	naloxone	0.80	1	60	4.7
Petrakis	2004	31.0	naltrexone	50.00	84	-	4.6
Gitlin	1981	8.0	naltrexone	85.70	7	-	4.5
Volavka	1977	7.0	naloxone	0.40	1	259	4.4
Janowsky	1977	8.0	naloxone	1.20	1	38	4.5
Lipinski	1979	9.0	naloxone	1.60	1	45	4.6
Naber	1983	12.0	naloxone	10.00	3	360	4.8
Emrich	1980	8.0	naloxone	40.00	1	240	4.5
Emrich	1979	20.0	naloxone	24.80	1	240	5.2
Kleinman	1982	8.0	naloxone	15.00	1	45	4.2
Davis	1977	14.0	naloxone	1.88	1	60	4.9
Lo	1983	13.0	naloxone	0.40	7	-	3.6
Pickar	1982	32.0	naloxone	21.18	1	184	5.4
Rapaport	1993	11.0	nalmefene	79.00	37	-	4.7
Emrich	1977	14.8	naloxone	3.58	1	175	5.0
Berger	1981	14.0	naloxone	10.00	1	108	4.8
Marchesi	1995	18.0	naltrexone	100.00	10	-	3.9
Schmauss	1987	3.3	buprenorphine	0.95	1	150	4.1
Gunne	1977	6.0	naloxone	0.40	1	90	4.0
Watson	1978	9.0	naloxone	10.00	1	98	4.3
Tatari	2014	60.0	naltrexone	100.00	84	-	4.8

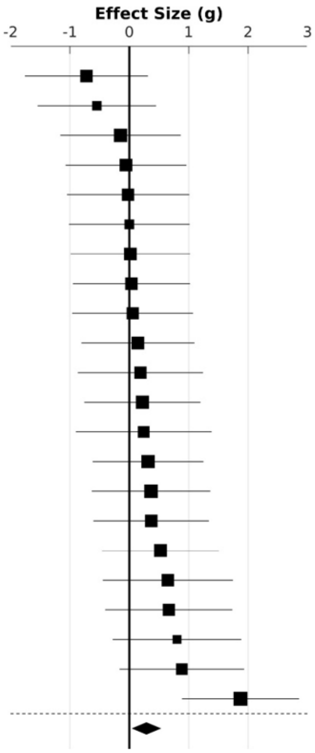

Average Effect: 0.29; CI = 0.05 - 0.52; P = 0.018

B

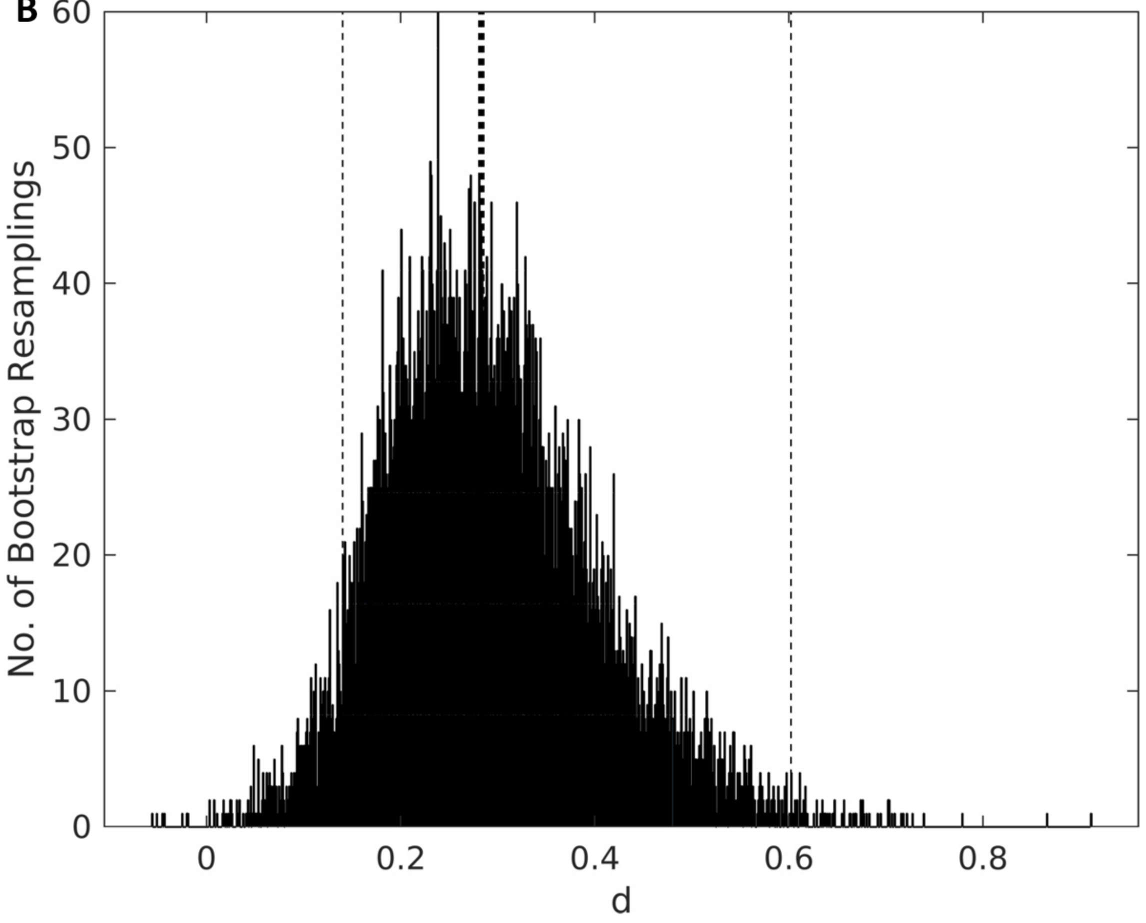

Supplemental Figure S5. All drugs combined on all scales combined weighted. A) Forest plot analysis of all drugs combined on all scales combined weighted. When studies reported multiple effects that met inclusion criteria (see Supplemental Methods), study *n* is reported here as the harmonic mean of all included effects (potentially resulting in fractional values of *n*). Dose and Time were reported as the mean of all included effects. Weight (%) is the normalized weight of each study, which is also proportional to the area of the box shown for each study in the forest. The whiskers for each study plot show the 95% confidence interval for that study. The diamond displayed at the bottom of the plot is centered on the average effect size, with the width of the diamond demonstrating the 95% confidence interval on the average effect. B) Histogram of bootstrap distribution of the average effect size for the analysis of all drugs combined on all scales combined weighted.

Total Scales

A

First Author	Year	n	Drug	Dose (mg)	Days	Time (m)	Weight
Kleinman	1982	9.0	naloxone	15.00	1	30	6.1
Petrakis	2004	31.0	naltrexone	50.00	84	-	6.8
Gitlin	1981	8.0	naltrexone	85.70	7	-	6.7
Volavka	1977	7.0	naloxone	0.40	1	259	6.3
Lipinski	1979	9.0	naloxone	1.60	1	45	7.1
Naber	1983	12.0	naloxone	10.00	3	360	8.1
Emrich	1980	8.0	naloxone	40.00	1	240	6.7
Emrich	1979	20.0	naloxone	24.80	1	240	10.2
Lo	1983	13.0	naloxone	0.40	7	-	3.9
Pickar	1982	32.0	naloxone	21.18	1	184	12.0
Rapaport	1993	11.0	nalmefene	79.00	37	-	7.5
Emrich	1977	14.8	naloxone	3.58	1	175	8.8
Marchesi	1995	18.0	naltrexone	100.00	10	-	4.7
Schmauss	1987	3.3	buprenorphine	0.95	1	150	5.2

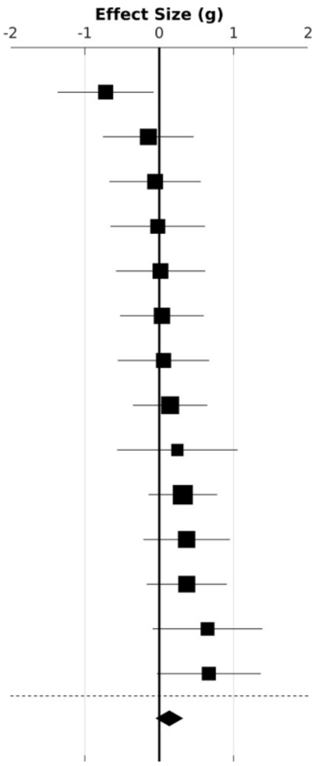

Average Effect: 0.13; CI = -0.04 - 0.31; P = 0.122

B

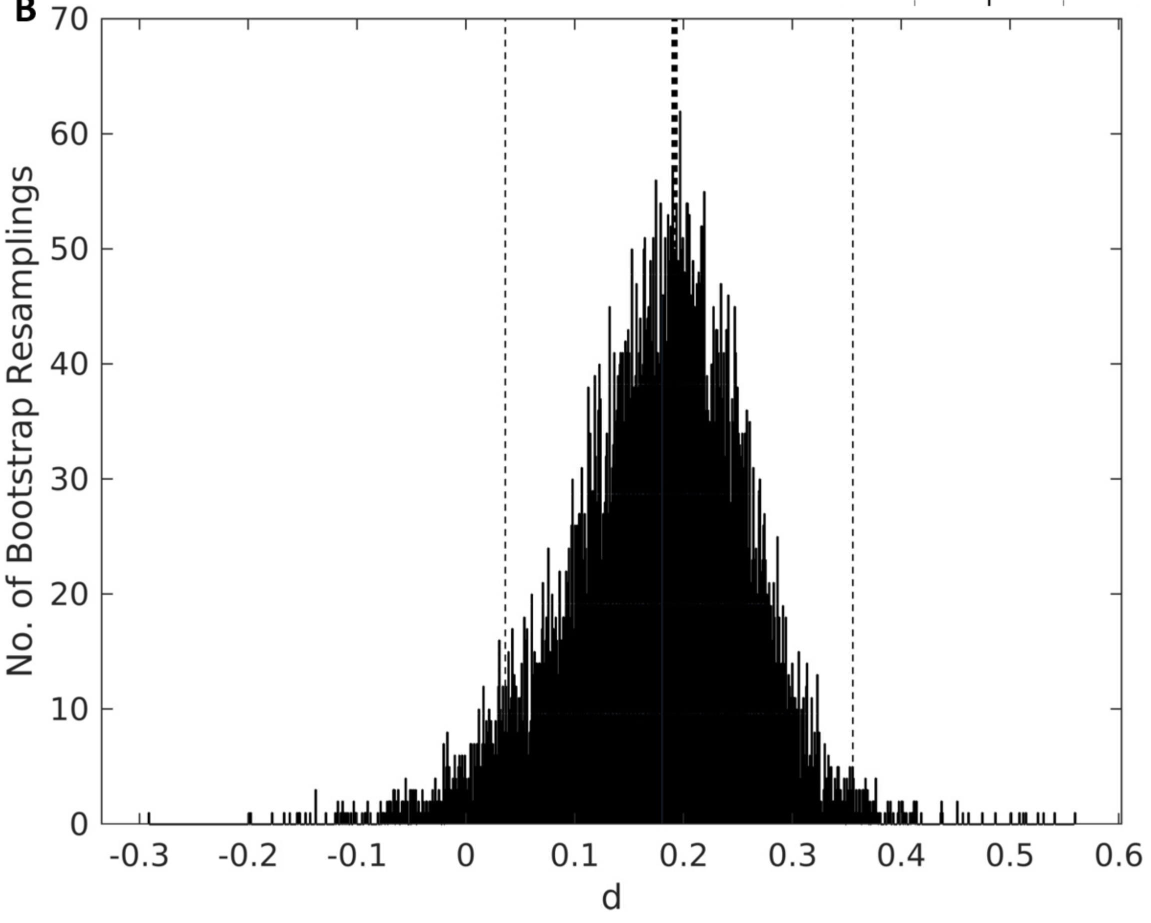

Supplemental Figure S6*.* All drugs combined on total scales weighted. A) Forest plot analysis of all drugs combined on total scales weighted. When studies reported multiple effects that met inclusion criteria (see Supplemental Methods), study *n* is reported here as the harmonic mean of all included effects (potentially resulting in fractional values of *n*). Dose and Time were reported as the mean of all included effects. Weight (%) is the normalized weight of each study, which is also proportional to the area of the box shown for each study in the forest. The whiskers for each study plot show the 95% confidence interval for that study. The diamond displayed at the bottom of the plot is centered on the average effect size, with the width of the diamond demonstrating the 95% confidence interval on the average effect. B) Histogram of bootstrap distribution of the average effect size for the analysis of all drugs combined on total scales weighted.

All Positive Scales Combined

A

First Author	Year	n	Drug	Dose (mg)	Days	Time (m)	Weight
Kurland	1977	12.0	naloxone	0.80	1	60	6.0
Petrakis	2004	31.0	naltrexone	50.00	84	-	5.8
Volavka	1977	7.0	naloxone	0.40	1	231	5.6
Emrich	1979	20.0	naloxone	24.80	1	240	6.6
Janowsky	1977	8.0	naloxone	1.20	1	38	5.8
Marchesi	1995	18.0	naltrexone	100.00	10	-	5.1
Kleinman	1982	8.0	naloxone	15.00	1	45	5.4
Davis	1977	14.0	naloxone	1.88	1	60	6.3
Pickar	1982	32.0	naloxone	21.18	1	120	6.9
Rapaport	1993	11.0	nalmefene	79.00	37	-	6.1
Emrich	1977	19.2	naloxone	3.58	1	178	6.5
Emrich	1980	8.0	naloxone	40.00	1	120	5.7
Berger	1981	14.0	naloxone	10.00	1	108	6.2
Schmauss	1987	3.3	buprenorphine	0.95	1	150	5.3
Gunne	1977	6.0	naloxone	0.40	1	90	5.1
Watson	1978	9.0	naloxone	10.00	1	98	5.5
Tatari	2014	60.0	naltrexone	100.00	84	-	6.2

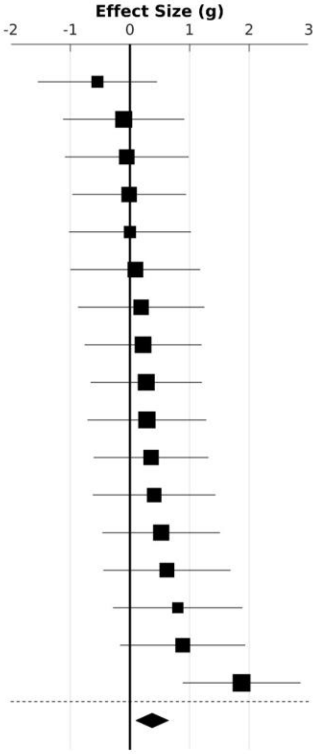

Average Effect: 0.37; CI = 0.10 - 0.64; P = 0.010

B

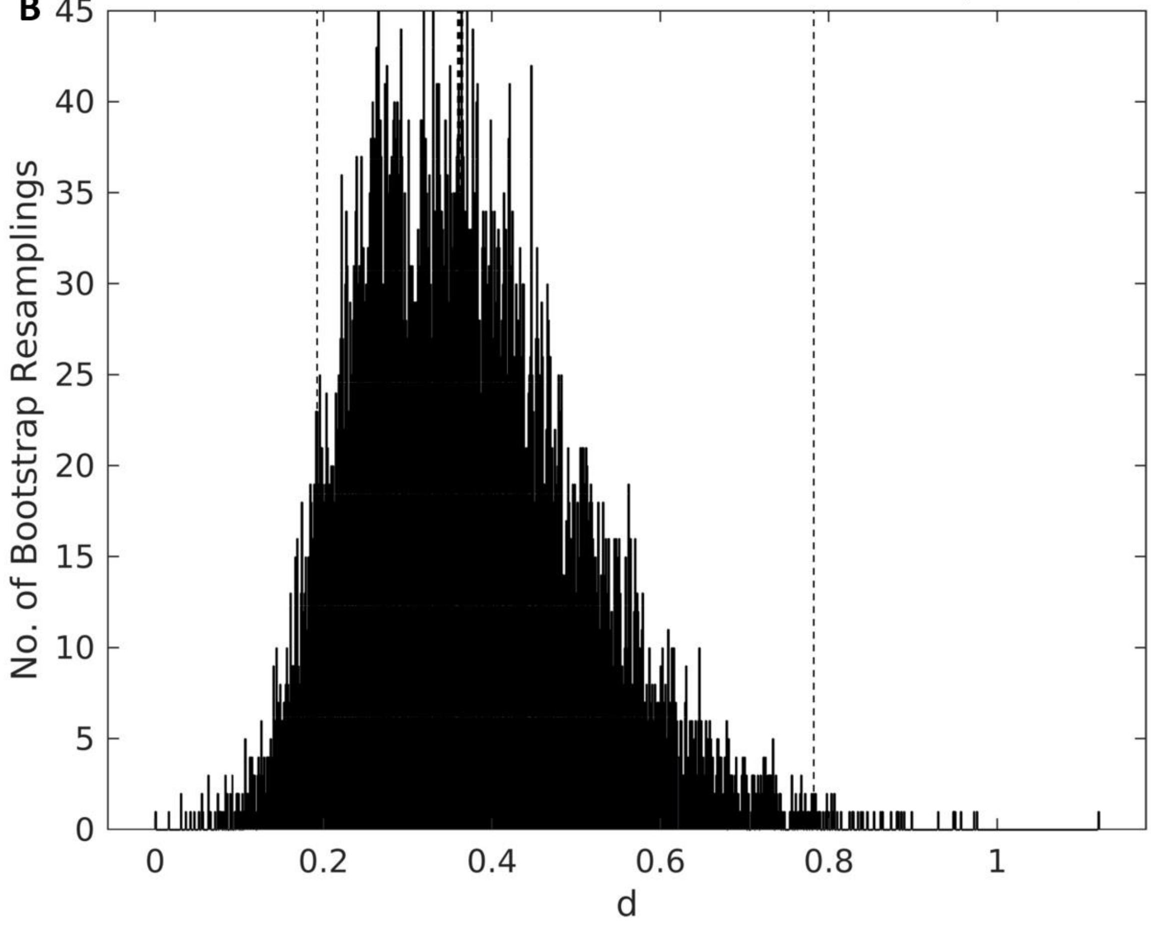

Supplemental Figure S7. All drugs combined on positive scales weighted. A) Forest plot analysis of all drugs combined on all positive scales weighted. When studies reported multiple effects that met inclusion criteria (see Supplemental Methods), study *n* is reported here as the harmonic mean of all included effects (potentially resulting in fractional values of *n*). Dose and Time were reported as the mean of all included effects. Weight (%) is the normalized weight of each study, which is also proportional to the area of the box shown for each study in the forest. The whiskers for each study plot show the 95% confidence interval for that study. The diamond displayed at the bottom of the plot is centered on the average effect size, with the width of the diamond demonstrating the 95% confidence interval on the average effect. B) Histogram of bootstrap distribution of the average effect size for the analysis of all drugs combined on all positive scales weighted.

Hallucination Scales

A

First Author	Year	n	Drug	Dose (mg)	Days	Time (m)	Weight
Kurland	1977	12.0	naloxone	0.80	1	60	11.2
Volavka	1977	7.0	naloxone	0.40	1	259	10.8
Janowsky	1977	8.0	naloxone	1.20	1	38	11.0
Davis	1977	14.0	naloxone	1.88	1	60	11.5
Pickar	1982	32.0	naloxone	21.18	1	120	11.9
Berger	1981	14.0	naloxone	10.00	1	108	11.4
Gunne	1977	6.0	naloxone	0.40	1	90	10.3
Watson	1978	9.0	naloxone	10.00	1	98	10.7
Tatari	2014	60.0	naltrexone	100.00	84	-	11.2

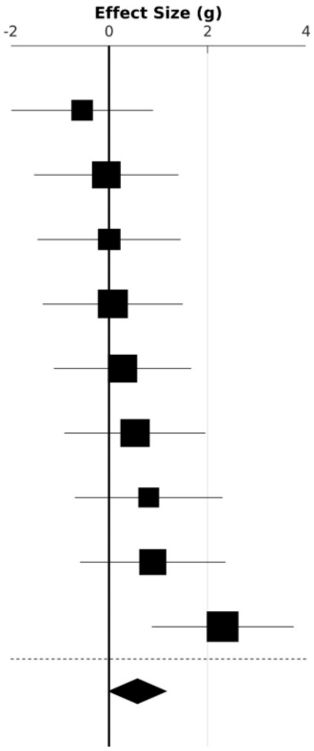

Average Effect: 0.57; CI = -0.01 - 1.16; P = 0.054

B

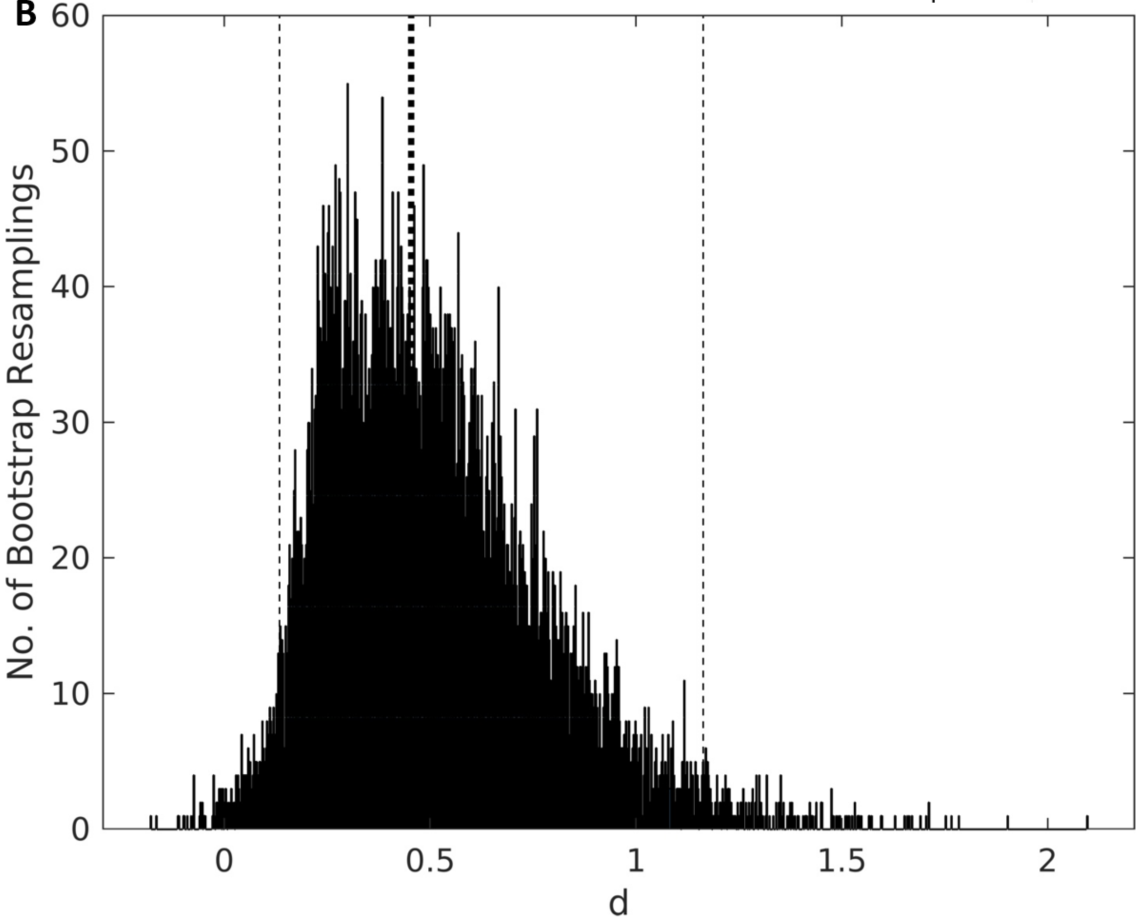

Supplemental Figure S8. All drugs combined on hallucination scales weighted. A) Forest plot analysis of all drugs combined on all hallucination subscales weighted. When studies reported multiple effects that met inclusion criteria (see Supplemental Methods), study *n* is reported here as the harmonic mean of all included effects (potentially resulting in fractional values of *n*). Dose and Time were reported as the mean of all included effects. Weight (%) is the normalized weight of each study, which is also proportional to the area of the box shown for each study in the forest. The whiskers for each study plot show the 95% confidence interval for that study. The diamond displayed at the bottom of the plot is centered on the average effect size, with the width of the diamond demonstrating the 95% confidence interval on the average effect. B) Histogram of bootstrap distribution of the average effect size for the analysis of all drugs combined on hallucination subscales weighted.

All Scales Combined

A

First Author	Year	n	Drug	Dose (mg)	Days	Time (m)	Weight
Kleinman	1982	9.0	naloxone	15.00	1	30	5.6
Kurland	1977	12.0	naloxone	0.80	1	60	6.5
Volavka	1977	7.0	naloxone	0.40	1	259	5.7
Janowsky	1977	8.0	naloxone	1.20	1	38	6.0
Lipinski	1979	9.0	naloxone	1.60	1	45	6.2
Naber	1983	12.0	naloxone	10.00	3	360	6.8
Emrich	1980	8.0	naloxone	40.00	1	240	6.0
Emrich	1979	20.0	naloxone	24.80	1	240	7.9
Kleinman	1982	8.0	naloxone	15.00	1	45	5.2
Davis	1977	14.0	naloxone	1.88	1	60	7.1
Lo	1983	13.0	naloxone	0.40	7	-	3.9
Pickar	1982	32.0	naloxone	21.18	1	184	8.8
Emrich	1977	14.8	naloxone	3.58	1	175	7.2
Berger	1981	14.0	naloxone	10.00	1	108	6.9
Gunne	1977	6.0	naloxone	0.40	1	90	4.7
Watson	1978	9.0	naloxone	10.00	1	98	5.3

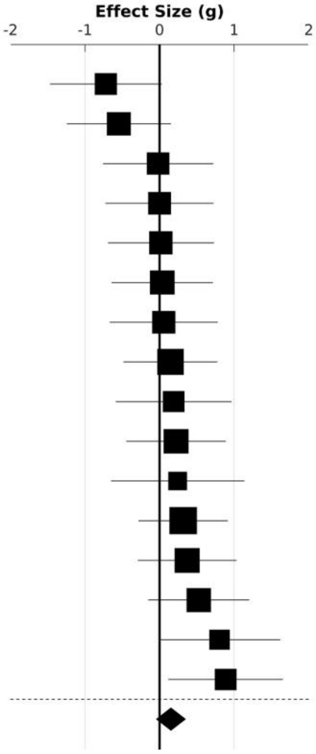

Average Effect: 0.15; CI = -0.04 - 0.34; P = 0.114

B

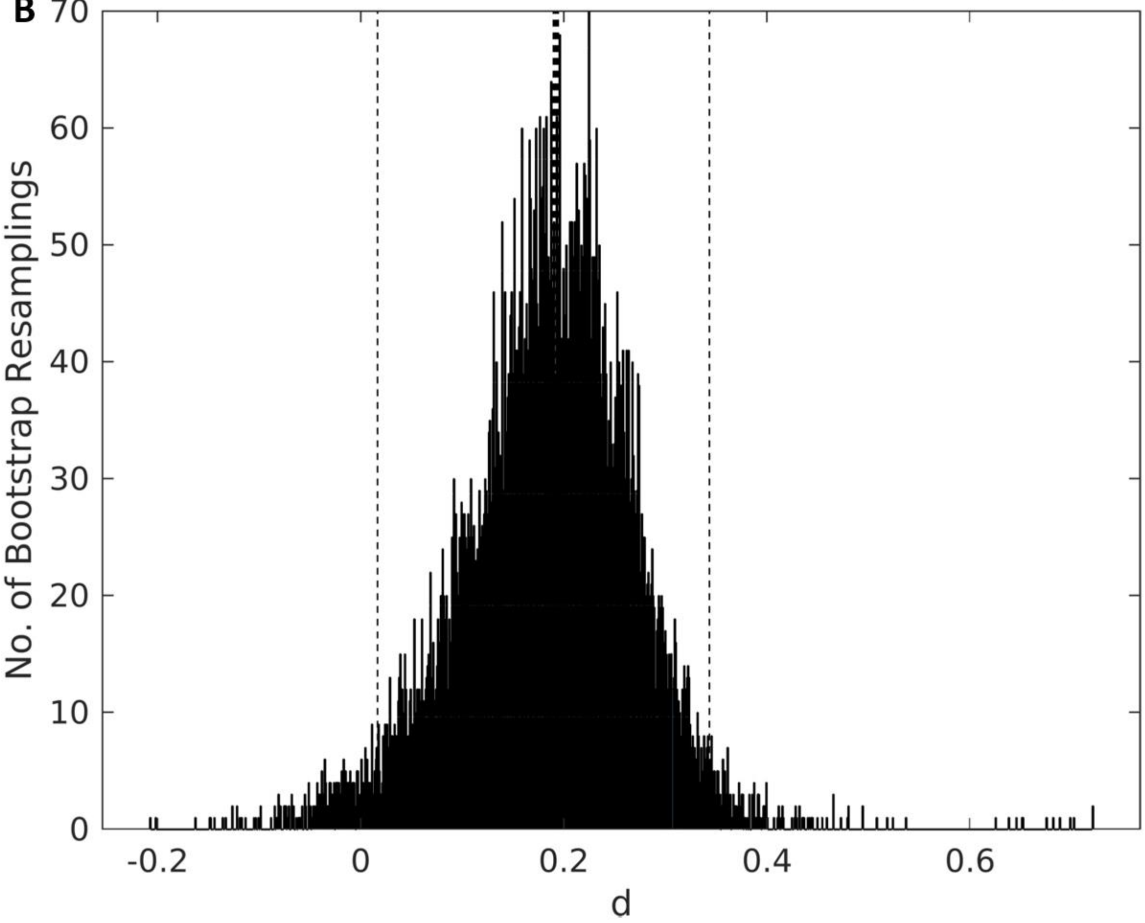

Supplemental Figure S9. Naloxone on all scales combined unweighted. A) Forest plot analysis of naloxone on all scales combined. When studies reported multiple effects that met inclusion criteria (see Supplemental Methods), study *n* is reported here as the harmonic mean of all included effects (potentially resulting in fractional values of *n*). Dose and Time were reported as the mean of all included effects. Weight (%) is the normalized weight of each study, which is also proportional to the area of the box shown for each study in the forest. The whiskers for each study plot show the 95% confidence interval for that study. The diamond displayed at the bottom of the plot is centered on the average effect size, with the width of the diamond demonstrating the 95% confidence interval on the average effect. B) Histogram of bootstrap distribution of the average effect size for the analysis of naloxone on all scales combined.

Total Scales

A

First Author	Year	n	Drug	Dose (mg)	Days	Time (m)	Weight
Kleinman	1982	9.0	naloxone	15.00	1	30	8.7
Volavka	1977	7.0	naloxone	0.40	1	259	9.0
Lipinski	1979	9.0	naloxone	1.60	1	45	10.1
Naber	1983	12.0	naloxone	10.00	3	360	11.7
Emrich	1980	8.0	naloxone	40.00	1	240	9.6
Emrich	1979	20.0	naloxone	24.80	1	240	14.9
Lo	1983	13.0	naloxone	0.40	7	-	5.4
Pickar	1982	32.0	naloxone	21.18	1	184	17.8
Emrich	1977	14.8	naloxone	3.58	1	175	12.7

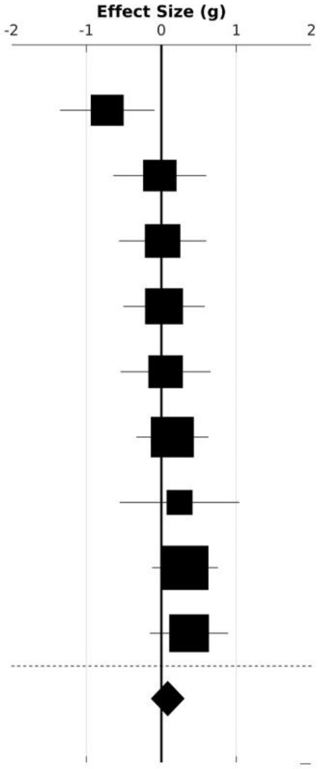

Average Effect: 0.08; CI = -0.13 - 0.30; P = 0.398

B

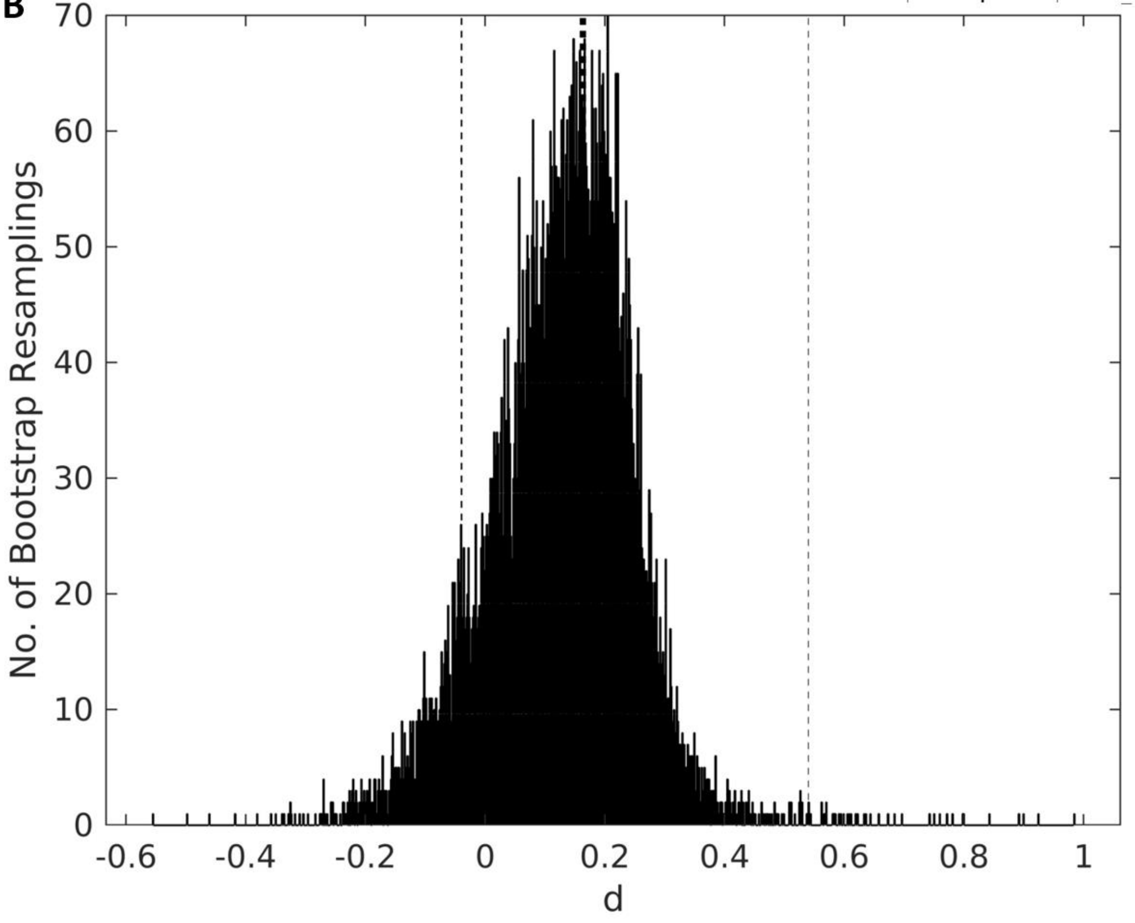

Supplemental Figure S10. Naloxone on total scales unweighted. A) Forest plot analysis of naloxone on total scales. When studies reported multiple effects that met inclusion criteria (see Supplemental Methods), study *n* is reported here as the harmonic mean of all included effects (potentially resulting in fractional values of *n*). Dose and Time were reported as the mean of all included effects. Weight (%) is the normalized weight of each study, which is also proportional to the area of the box shown for each study in the forest. The whiskers for each study plot show the 95% confidence interval for that study. The diamond displayed at the bottom of the plot is centered on the average effect size, with the width of the diamond demonstrating the 95% confidence interval on the average effect. B) Histogram of bootstrap distribution of the average effect size for the analysis of naloxone on total scales.

All Positive Scales Combined

A

First Author	Year	n	Drug	Dose (mg)	Days	Time (m)	Weight
Kurland	1977	12.0	naloxone	0.80	1	60	8.3
Volavka	1977	7.0	naloxone	0.40	1	231	7.1
Emrich	1979	20.0	naloxone	24.80	1	240	10.5
Janowsky	1977	8.0	naloxone	1.20	1	38	7.7
Kleinman	1982	8.0	naloxone	15.00	1	45	6.6
Davis	1977	14.0	naloxone	1.88	1	60	9.2
Pickar	1982	32.0	naloxone	21.18	1	120	11.7
Emrich	1977	19.2	naloxone	3.58	1	178	10.1
Emrich	1980	8.0	naloxone	40.00	1	120	7.4
Berger	1981	14.0	naloxone	10.00	1	108	8.9
Gunne	1977	6.0	naloxone	0.40	1	90	5.9
Watson	1978	9.0	naloxone	10.00	1	98	6.7

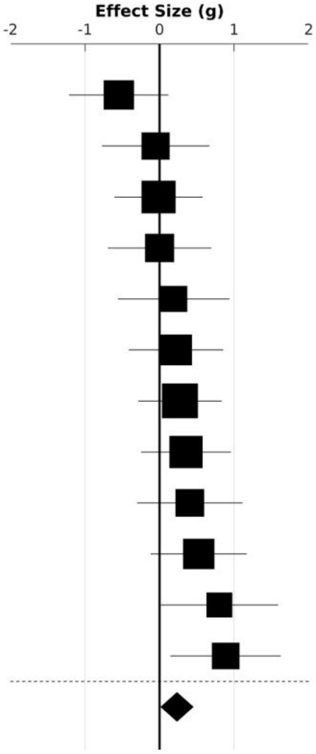

Average Effect: 0.23; CI = 0.02 - 0.45; P = 0.036

B

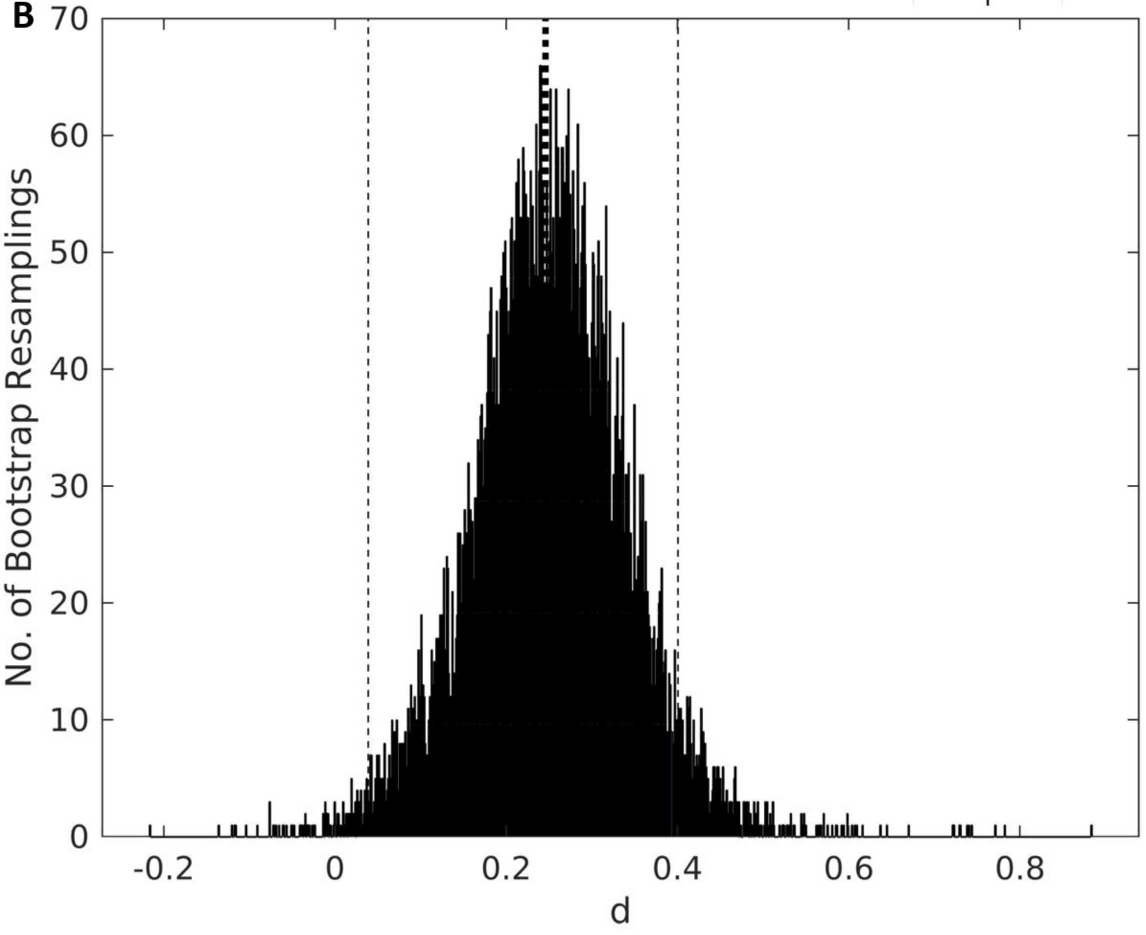

Supplemental Figure S11. Naloxone on positive scales unweighted. A) Forest plot analysis of naloxone on positive scales. When studies reported multiple effects that met inclusion criteria (see Supplemental Methods), study *n* is reported here as the harmonic mean of all included effects (potentially resulting in fractional values of *n*). Dose and Time were reported as the mean of all included effects. Weight (%) is the normalized weight of each study, which is also proportional to the area of the box shown for each study in the forest. The whiskers for each study plot show the 95% confidence interval for that study. The diamond displayed at the bottom of the plot is centered on the average effect size, with the width of the diamond demonstrating the 95% confidence interval on the average effect. B) Histogram of bootstrap distribution of the average effect size for the analysis of naloxone on positive scales.

Hallucination Scales

A

First Author	Year	n	Drug	Dose (mg)	Days	Time (m)	Weight
Kurland	1977	12.0	naloxone	0.80	1	60	12.7
Volavka	1977	7.0	naloxone	0.40	1	259	11.3
Janowsky	1977	8.0	naloxone	1.20	1	38	12.0
Davis	1977	14.0	naloxone	1.88	1	60	13.8
Pickar	1982	32.0	naloxone	21.18	1	120	16.1
Berger	1981	14.0	naloxone	10.00	1	108	13.3
Gunne	1977	6.0	naloxone	0.40	1	90	9.8
Watson	1978	9.0	naloxone	10.00	1	98	10.9

Average Effect: 0.22; CI = -0.11 - 0.56; P = 0.161

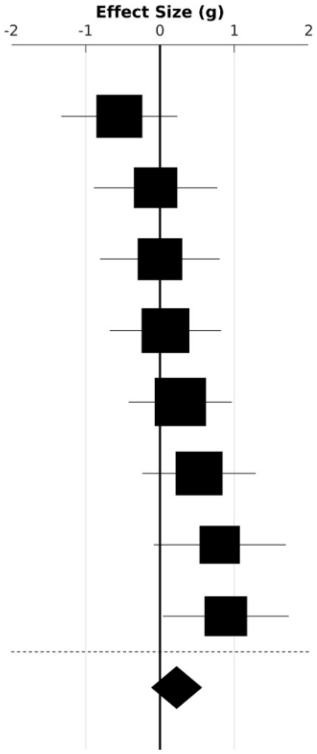

B

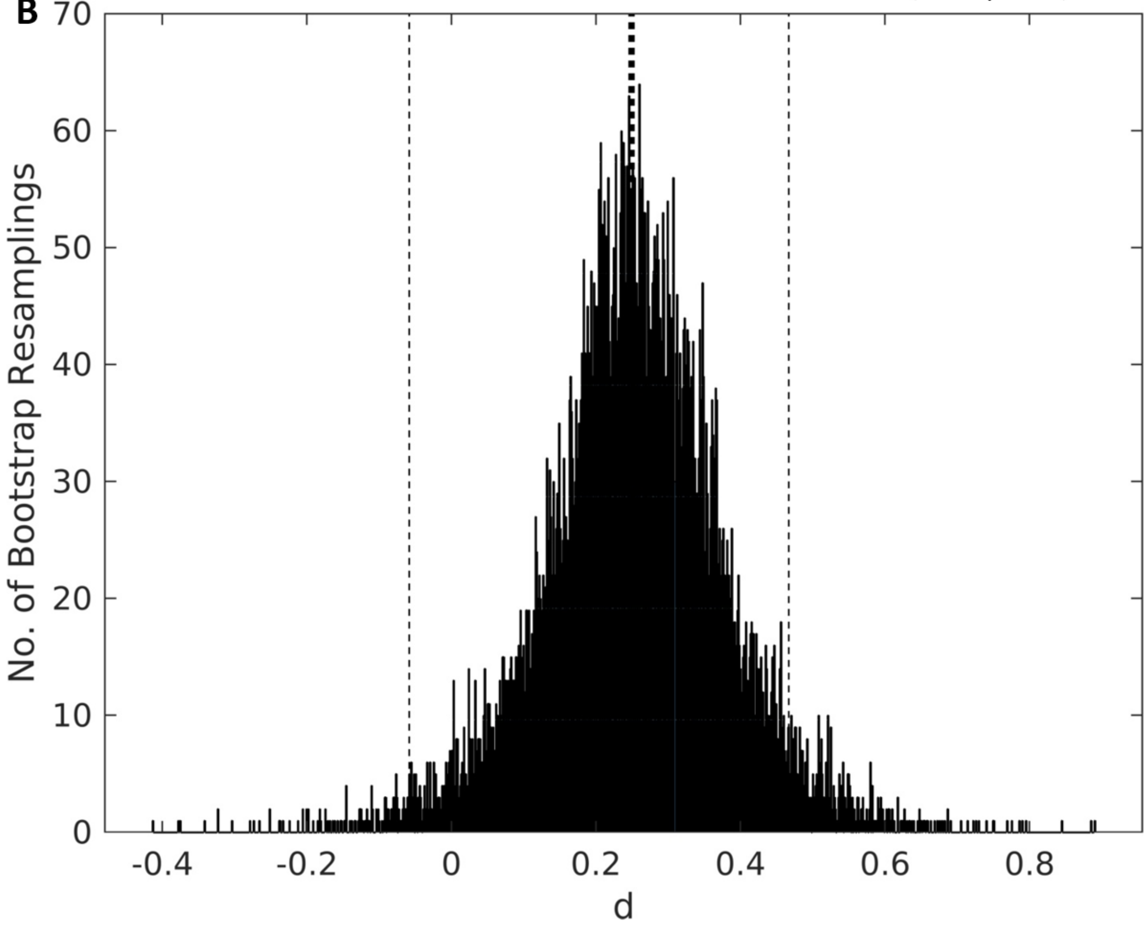

Supplemental Figure S12*.* Naloxone on hallucination scales unweighted. A) Forest plot analysis of naloxone on hallucination subscales. When studies reported multiple effects that met inclusion criteria (see Supplemental Methods), study *n* is reported here as the harmonic mean of all included effects (potentially resulting in fractional values of *n*). Dose and Time were reported as the mean of all included effects. Weight (%) is the normalized weight of each study, which is also proportional to the area of the box shown for each study in the forest. The whiskers for each study plot show the 95% confidence interval for that study. The diamond displayed at the bottom of the plot is centered on the average effect size, with the width of the diamond demonstrating the 95% confidence interval on the average effect. B) Histogram of bootstrap distribution of the average effect size for the analysis of naloxone on hallucination subscales.

All Scales Combined

A

First Author	Year	n	Drug	Dose (mg)	Days	Time (m)	Weight
Kleinman	1982	9.0	naloxone	15.00	1	30	5.6
Kurland	1977	12.0	naloxone	0.80	1	60	6.5
Volavka	1977	7.0	naloxone	0.40	1	259	5.7
Janowsky	1977	8.0	naloxone	1.20	1	38	6.0
Lipinski	1979	9.0	naloxone	1.60	1	45	6.2
Naber	1983	12.0	naloxone	10.00	3	360	6.8
Emrich	1980	8.0	naloxone	40.00	1	240	6.0
Emrich	1979	20.0	naloxone	24.80	1	240	7.9
Kleinman	1982	8.0	naloxone	15.00	1	45	5.2
Davis	1977	14.0	naloxone	1.88	1	60	7.1
Lo	1983	13.0	naloxone	0.40	7	-	3.9
Pickar	1982	32.0	naloxone	21.18	1	184	8.8
Emrich	1977	14.8	naloxone	3.58	1	175	7.2
Berger	1981	14.0	naloxone	10.00	1	108	6.9
Gunne	1977	6.0	naloxone	0.40	1	90	4.7
Watson	1978	9.0	naloxone	10.00	1	98	5.3

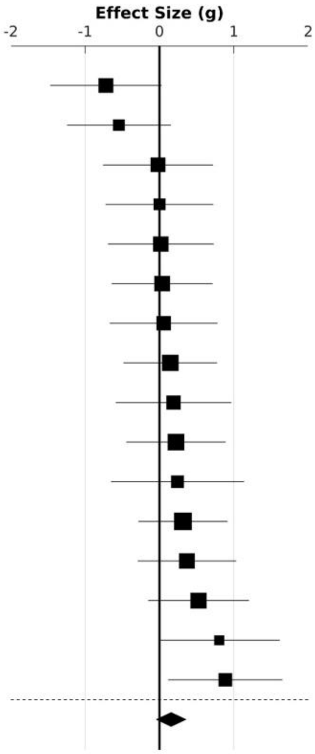

Average Effect: 0.16; CI = -0.04 - 0.35; P = 0.110

B

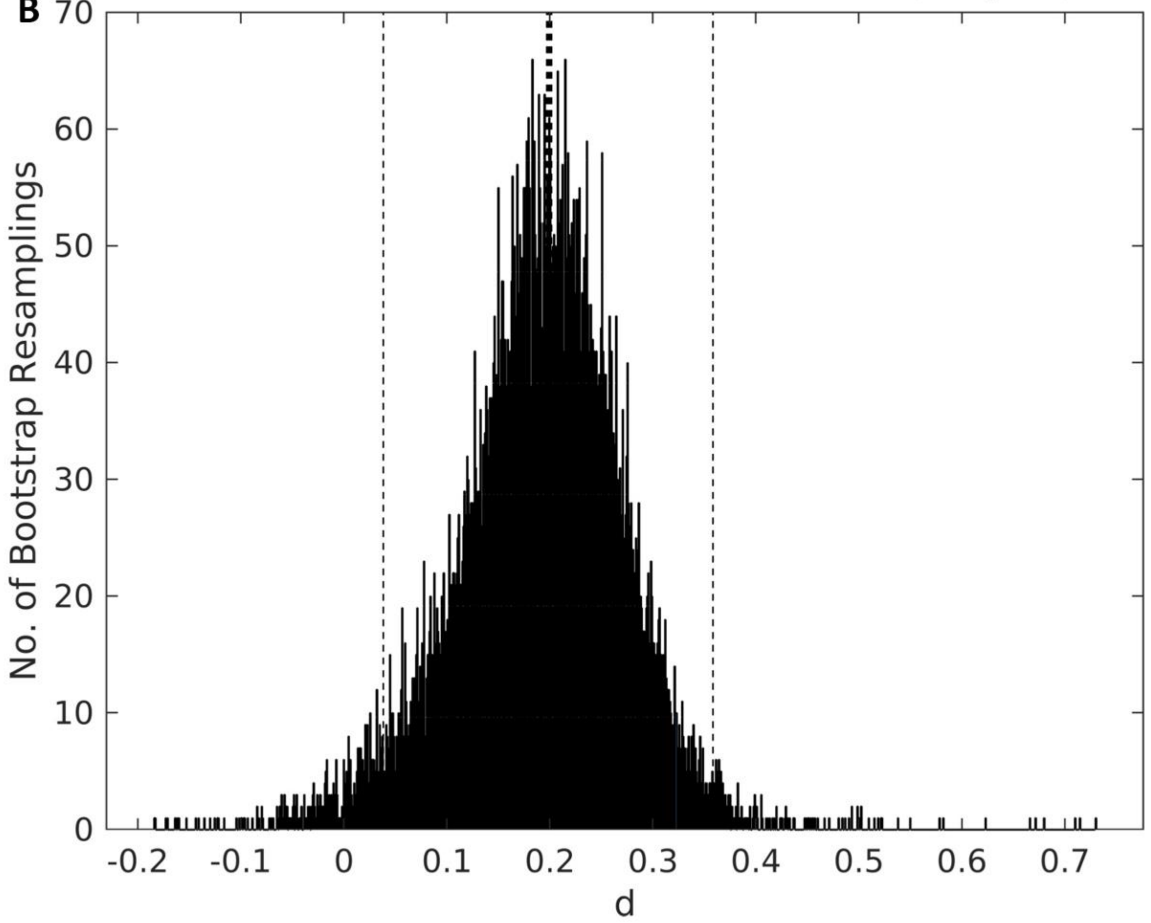

Supplemental Figure S13*. Naloxone on all scales combined weighted.* A) Forest plot analysis of naloxone on all scales combined weighted. When studies reported multiple effects that met inclusion criteria (see Supplemental Methods), study *n* is reported here as the harmonic mean of all included effects (potentially resulting in fractional values of *n*). Dose and Time were reported as the mean of all included effects. Weight (%) is the normalized weight of each study, which is also proportional to the area of the box shown for each study in the forest. The whiskers for each study plot show the 95% confidence interval for that study. The diamond displayed at the bottom of the plot is centered on the average effect size, with the width of the diamond demonstrating the 95% confidence interval on the average effect. B) Histogram of bootstrap distribution of the average effect size for the analysis of naloxone on all scales combined weighted.

Total Scales

A

First Author	Year	n	Drug	Dose (mg)	Days	Time (m)	Weight
Kleinman	1982	9.0	naloxone	15.00	1	30	8.7
Volavka	1977	7.0	naloxone	0.40	1	259	9.0
Lipinski	1979	9.0	naloxone	1.60	1	45	10.1
Naber	1983	12.0	naloxone	10.00	3	360	11.7
Emrich	1980	8.0	naloxone	40.00	1	240	9.6
Emrich	1979	20.0	naloxone	24.80	1	240	14.9
Lo	1983	13.0	naloxone	0.40	7	-	5.4
Pickar	1982	32.0	naloxone	21.18	1	184	17.8
Emrich	1977	14.8	naloxone	3.58	1	175	12.7

Average Effect: 0.08; CI = -0.14 - 0.30; P = 0.438

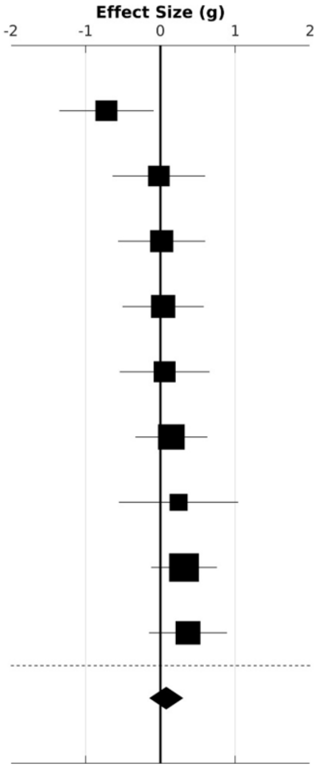

B

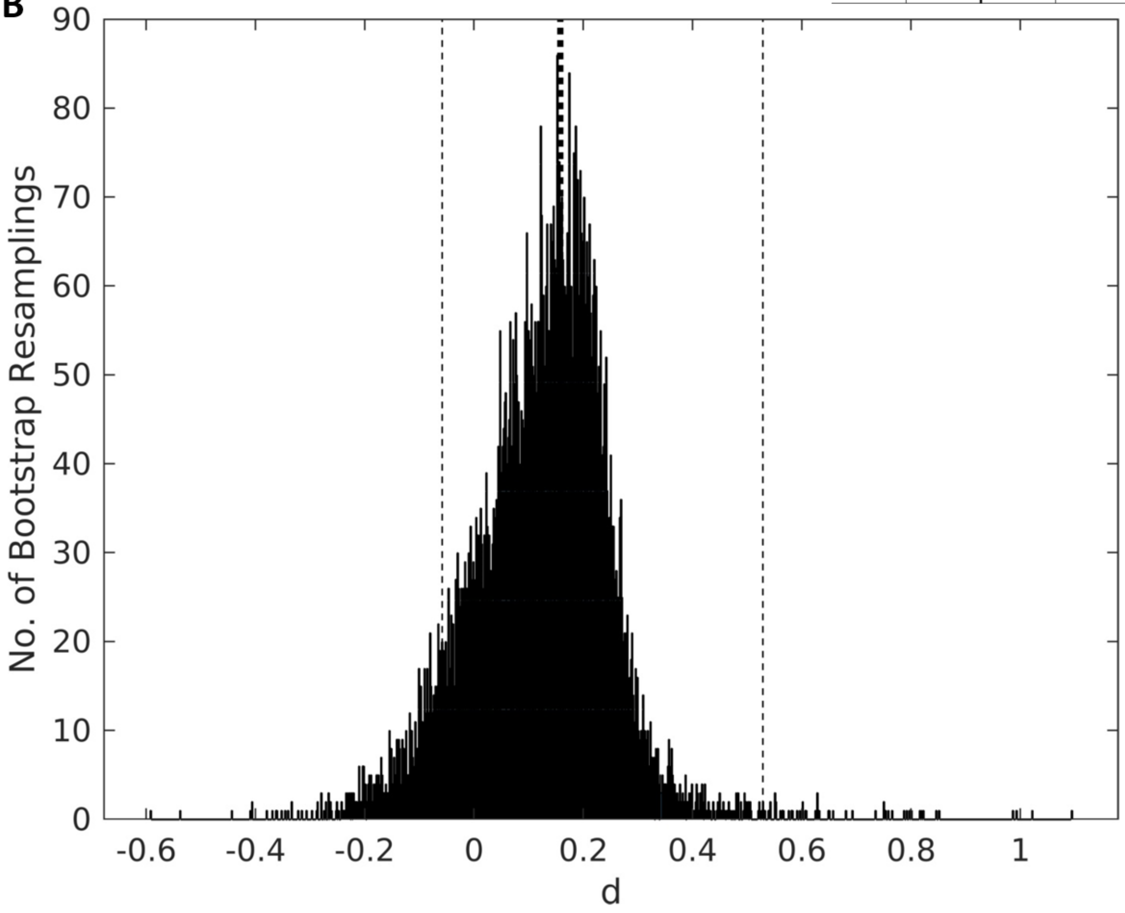

Supplemental Figure S14*. *Naloxone on total scales combined weighted. A) Forest plot analysis of naloxone on total scales weighted. When studies reported multiple effects that met inclusion criteria (see Supplemental Methods), study *n* is reported here as the harmonic mean of all included effects (potentially resulting in fractional values of *n*). Dose and Time were reported as the mean of all included effects. Weight (%) is the normalized weight of each study, which is also proportional to the area of the box shown for each study in the forest. The whiskers for each study plot show the 95% confidence interval for that study. The diamond displayed at the bottom of the plot is centered on the average effect size, with the width of the diamond demonstrating the 95% confidence interval on the average effect. B) Histogram of bootstrap distribution of the average effect size for the analysis of naloxone on total scales weighted.

All Positive Scales Combined

A

First Author	Year	n	Drug	Dose (mg)	Days	Time (m)	Weight
Kurland	1977	12.0	naloxone	0.80	1	60	8.3
Volavka	1977	7.0	naloxone	0.40	1	231	7.1
Emrich	1979	20.0	naloxone	24.80	1	240	10.5
Janowsky	1977	8.0	naloxone	1.20	1	38	7.7
Kleinman	1982	8.0	naloxone	15.00	1	45	6.6
Davis	1977	14.0	naloxone	1.88	1	60	9.2
Pickar	1982	32.0	naloxone	21.18	1	120	11.7
Emrich	1977	19.2	naloxone	3.58	1	178	10.1
Emrich	1980	8.0	naloxone	40.00	1	120	7.4
Berger	1981	14.0	naloxone	10.00	1	108	8.9
Gunne	1977	6.0	naloxone	0.40	1	90	5.9
Watson	1978	9.0	naloxone	10.00	1	98	6.7

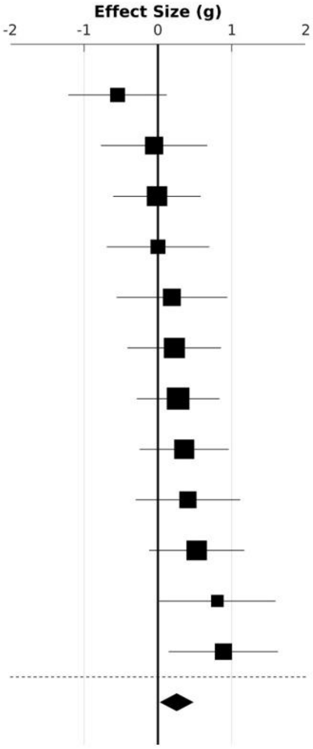

Average Effect: 0.25; CI = 0.03 - 0.47; P = 0.028

B

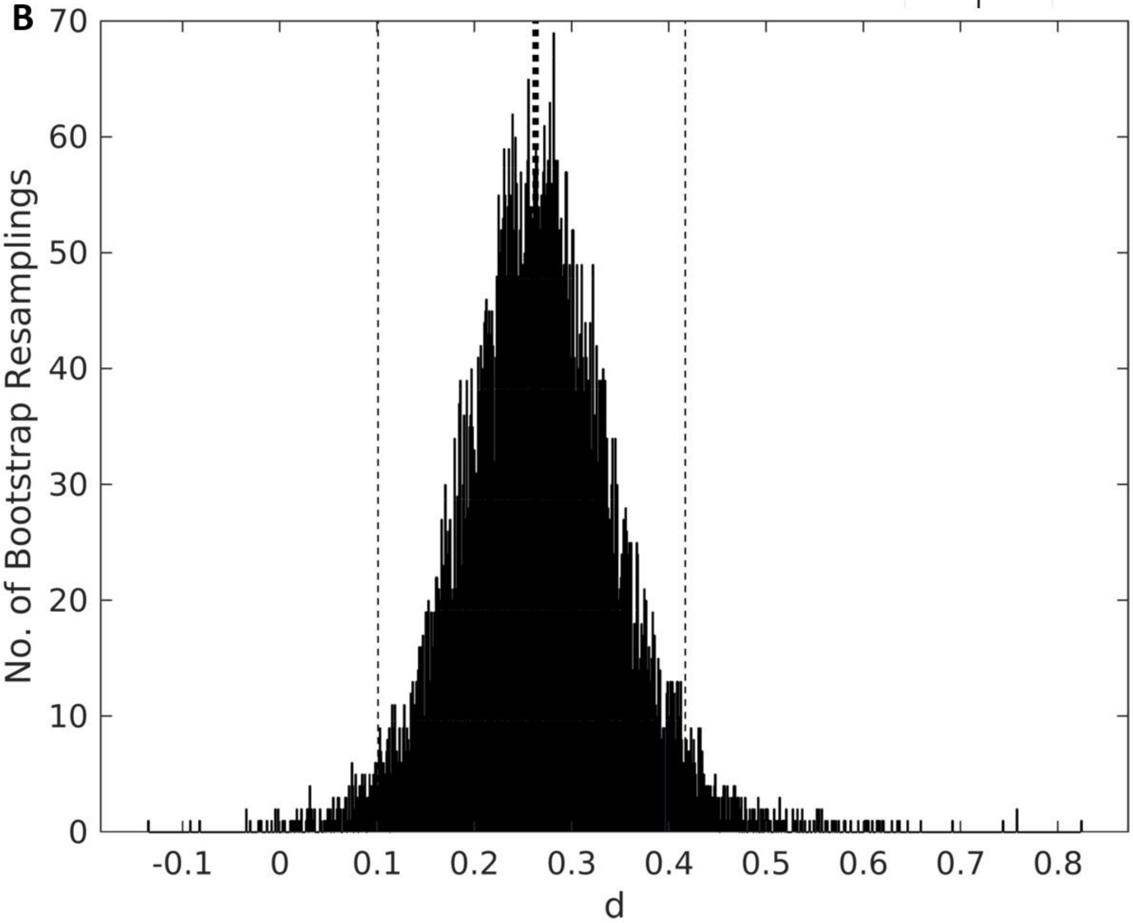

Supplemental Figure S15*.* Naloxone on all positive scales combined weighted. A) Forest plot analysis of naloxone on all positive scales combined weighted. When studies reported multiple effects that met inclusion criteria (see Supplemental Methods), study *n* is reported here as the harmonic mean of all included effects (potentially resulting in fractional values of *n*). Dose and Time were reported as the mean of all included effects. Weight (%) is the normalized weight of each study, which is also proportional to the area of the box shown for each study in the forest. The whiskers for each study plot show the 95% confidence interval for that study. The diamond displayed at the bottom of the plot is centered on the average effect size, with the width of the diamond demonstrating the 95% confidence interval on the average effect. B) Histogram of bootstrap distribution of the average effect size for the analysis of naloxone on all positive scales combined weighted.

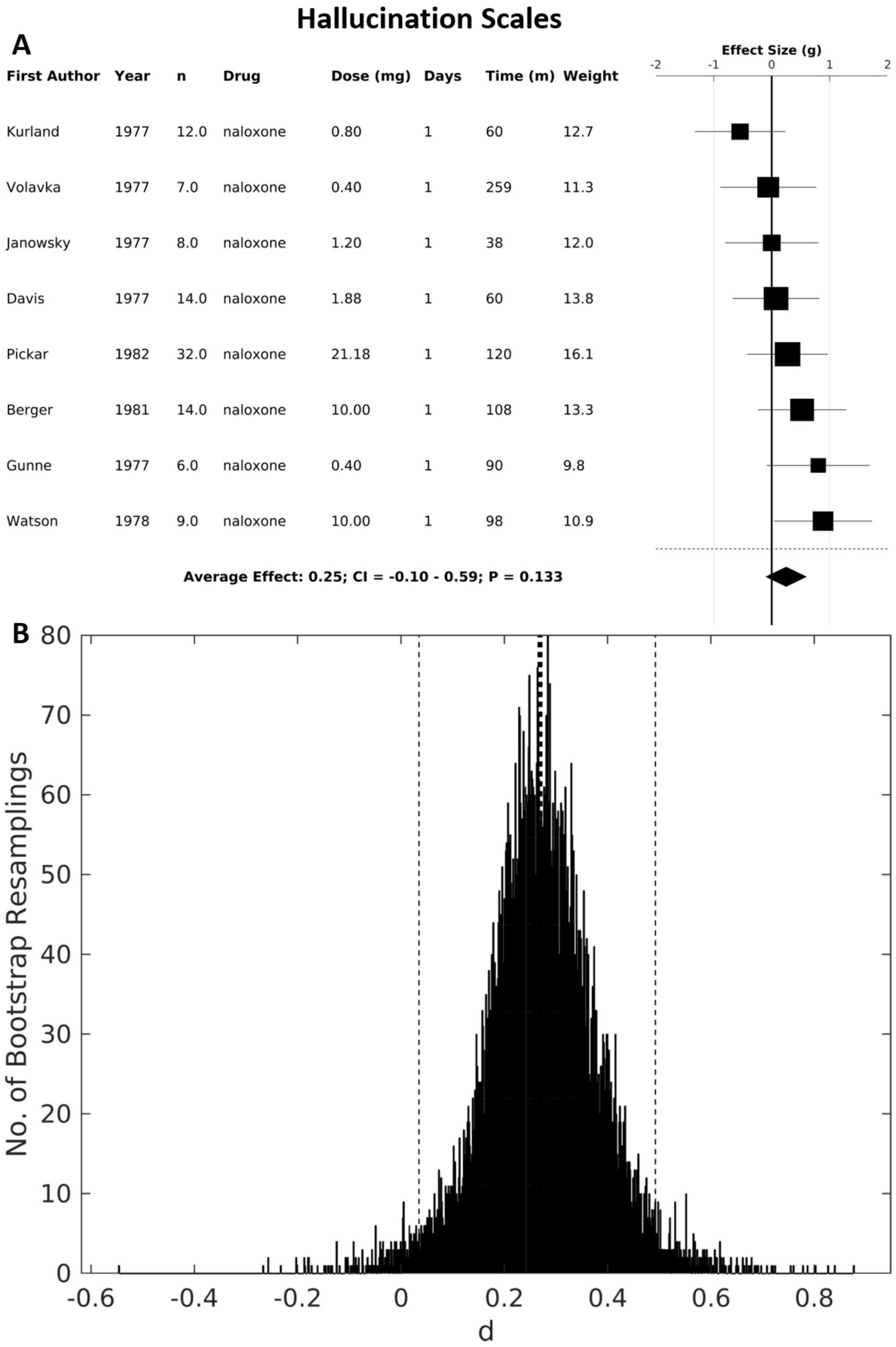
Hallucination Scales
A
First Author Year n Drug Dose (mg) Days Time (m) Weight
Kurland 1977 12.0 naloxone 0.80 1 60 12.7
Volavka 1977 7.0 naloxone 0.40 1 259 11.3
Janowsky 1977 8.0 naloxone 1.20 1 38 12.0
Davis 1977 14.0 naloxone 1.88 1 60 13.8
Pickar 1982 32.0 naloxone 21.18 1 120 16.1
Berger 1981 14.0 naloxone 10.00 1 108 13.3
Gunne 1977 6.0 naloxone 0.40 1 90 9.8
Watson 1978 9.0 naloxone 10.00 1 98 10.9
Average Effect: 0.25; CI = -0.10 - 0.59; P = 0.133
Effect Size (g)
-2
-1
0
1
2
B
No. of Bootstrap Resamplings
0
10
20
30
40
50
60
70
80
d
-0.6
-0.4
-0.2
0
0.2
0.4
0.6
0.8

Supplemental Figure S16*. *Naloxone on hallucination scales combined weighted. A) A) Forest plot analysis of naloxone on hallucination subscales weighted. When studies reported multiple effects that met inclusion criteria (see Supplemental Methods), study *n* is reported here as the harmonic mean of all included effects (potentially resulting in fractional values of *n*). Dose and Time were reported as the mean of all included effects. Weight (%) is the normalized weight of each study, which is also proportional to the area of the box shown for each study in the forest. The whiskers for each study plot show the 95% confidence interval for that study. The diamond displayed at the bottom of the plot is centered on the average effect size, with the width of the diamond demonstrating the 95% confidence interval on the average effect. B) Histogram of bootstrap distribution of the average effect size for the analysis of naloxone on hallucination subscales weighted.

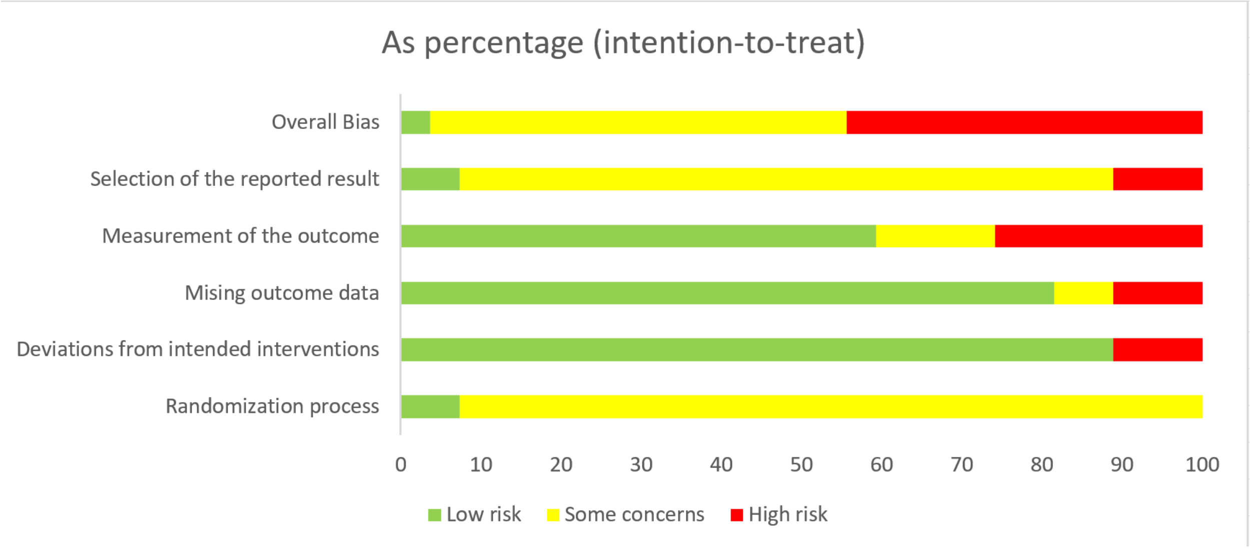

Supplemental Figure S17. Cochrane assessment of risk of bias overview of percentage of trials with bias. This details the risk of overall bias and for each of the domains as a percentage of records that fall into each category.

Study ID	Randomization process	Deviations from intended interventions	Missing outcome data	Measurement of the outcome	Selection of the reported result	Overall
Gunne et al. 1977	?	+	+	–	?	–
Volovka et al., 1977	?	+	+	–	?	–
Kurland et al., 1977	?	+	+	–	?	–
Emrich et al., 1977	?	+	+	–	?	–
Davis et al., 1977	?	+	+	+	?	!
Janowsky et al., 1977	?	+	+	+	?	!
Watson et al., 1978	?	+	+	+	?	!
Gunne et al., 1979	?	+	+	?	?	!
Lipinski et al., 1979	?	+	–	?	?	–
Emrich et al., 1979	?	+	+	+	?	!
Lehman et al., 1979	?	–	+	+	?	–
Emrich et al., 1980	?	–	+	–	?	–
Gitlin et al., 1981	?	+	+	+	?	!
Berger et al., 1981	?	+	+	+	?	!
Freeman et al., 1981	?	+	?	+	?	!
Kleinman et al., 1982	?	–	–	?	?	–
Pickar et al., 1982	?	+	+	?	–	–
Naber et al. 1983	?	+	+	+	?	!
Lo et al., 1983	?	+	+	+	?	!
Cohen et al., 1985	?	+	+	+	?	!
Schmauss et al., 1987	?	+	+	+	?	!
Pickar et al., 1989	?	+	+	–	?	–
Marchesi et al., 1992	?	+	–	+	–	–
Rapaport et al., 1993	?	+	+	–	–	–
Marchesi et al., 1995	?	+	+	+	+	!
Petrakis et al., 2004	+	+	?	+	?	!
Tatari et al., 2014	+	+	+	+	+	+

+ Low risk
? Some concerns
– High risk

Supplemental Figure S18*. Cochrane assessment of risk of bias per trial breakdown by domain.* This details the risk of bias for each of the included records for each domain of the Cochrane risk of bias tool 2.

Supplemental Table S1 PRISMA checklist

Section/topic	#	Checklist item	Reported on page #
**TITLE**			
Title	1	Identify the report as a systematic review, meta-analysis, or both.	Page 1
**ABSTRACT**			
Structured summary	2	Provide a structured summary including, as applicable: background; objectives; data sources; study eligibility criteria, participants, and interventions; study appraisal and synthesis methods; results; limitations; conclusions and implications of key findings; systematic review registration number.	Pages 2, we did not register our review.
**INTRODUCTION**			
Rationale	3	Describe the rationale for the review in the context of what is already known.	Page 3
Objectives	4	Provide an explicit statement of questions being addressed with reference to participants, interventions, comparisons, outcomes, and study design (PICOS).	Page 3
**METHODS**			
Protocol and registration	5	Indicate if a review protocol exists, if and where it can be accessed (e.g., Web address), and, if available, provide registration information including registration number.	Page 4 we did not have a pre-registered review protocol.
Eligibility criteria	6	Specify study characteristics (e.g., PICOS, length of follow-up) and report characteristics (e.g., years considered, language, publication status) used as criteria for eligibility, giving rationale.	page 4
Information sources	7	Describe all information sources (e.g., databases with dates of coverage, contact with study authors to identify additional studies) in the search and date last searched.	Page 4-5 of manuscript, Figure 1,

			Supplement page 1
Search	8	Present full electronic search strategy for at least one database, including any limits used, such that it could be repeated.	Page 5 of manuscript. Full search strings are in **Supplemental Table S2**
Study selection	9	State the process for selecting studies (i.e., screening, eligibility, included in systematic review, and, if applicable, included in the meta-analysis).	Page 4-5 of manuscript and page 1 of supplement.
Data collection process	10	Describe method of data extraction from reports (e.g., piloted forms, independently, in duplicate) and any processes for obtaining and confirming data from investigators.	Pages 5-6 of manuscript and pages 1-2 of supplement
Data items	11	List and define all variables for which data were sought (e.g., PICOS, funding sources) and any assumptions and simplifications made.	Pages 5-6 of the manuscript and pages 1-2 of the supplement
Risk of bias in individual studies	12	Describe methods used for assessing risk of bias of individual studies (including specification of whether this was done at the study or outcome level), and how this information is to be used in any data synthesis.	Page 8 of manuscript and pages 2-5 in the supplement under **Supplemental Methods** and **Supplemental**

			**Figure S17-S18**
Summary measures	13	State the principal summary measures (e.g., risk ratio, difference in means).	Page 6 of manuscript.
Synthesis of results	14	Describe the methods of handling data and combining results of studies, if done, including measures of consistency (e.g., I^2) for each meta-analysis.	Pages 7-8 of manuscript.
Section/topic	#	Checklist item	Reported on page #
Risk of bias across studies	15	Specify any assessment of risk of bias that may affect the cumulative evidence (e.g., publication bias, selective reporting within studies).	Pages 8-9 of manuscript.
Additional analyses	16	Describe methods of additional analyses (e.g., sensitivity or subgroup analyses, meta-regression), if done, indicating which were pre-specified.	Page 7 and page 9 of manuscript.
**RESULTS**			
Study selection	17	Give numbers of studies screened, assessed for eligibility, and included in the review, with reasons for exclusions at each stage, ideally with a flow diagram.	Page 9-10 of manuscript, **Figure 1.** And **Supplemental Tables S7-S9**
Study characteristics	18	For each study, present characteristics for which data were extracted (e.g., study size, PICOS, follow-up period) and provide the citations.	Page 10 of manuscript, In supplement page 5 under **Supplemental Results**, full details for all studies with citations in

			**Supplemental Tables S6-S9**
Risk of bias within studies	19	Present data on risk of bias of each study and, if available, any outcome level assessment (see item 12).	Data reported in supplement **Supplemental Figures S17-S18**
Results of individual studies	20	For all outcomes considered (benefits or harms), present, for each study: (a) simple summary data for each intervention group (b) effect estimates and confidence intervals, ideally with a forest plot.	Pages 11-12 **Figure 2 and Table 2** of manuscript**..** In supplement pages 5-6 under **Supplemental Results**, **Supplemental Figures S2-S15.**
Synthesis of results	21	Present results of each meta-analysis done, including confidence intervals and measures of consistency.	Page 11-12 of **Table 2** of manuscript. In supplement pages 5-6 under **Supplemental Results**, **Supplemental Figures S2-S15** and **Supplemental**

			**Tables S10-S14**.
Risk of bias across studies	22	Present results of any assessment of risk of bias across studies (see Item 15).	Page 10 of manuscript and in **Supplemental Figure S1**
Additional analysis	23	Give results of additional analyses, if done (e.g., sensitivity or subgroup analyses, meta-regression [see Item 16]).	Page 12 of manuscript and in supplement pages 5-6 under **Supplemental Results** and **Supplemental Tables S12-S14**
**DISCUSSION**			
Summary of evidence	24	Summarize the main findings including the strength of evidence for each main outcome; consider their relevance to key groups (e.g., healthcare providers, users, and policy makers).	Manuscript page 12-15
Limitations	25	Discuss limitations at study and outcome level (e.g., risk of bias), and at review-level (e.g., incomplete retrieval of identified research, reporting bias).	Manuscript page 14-15
Conclusions	26	Provide a general interpretation of the results in the context of other evidence, and implications for future research.	Manuscript page 15
**FUNDING**			
Funding	27	Describe sources of funding for the systematic review and other support (e.g., supply of data); role of funders for the systematic review.	Manuscript page 15

Supplemental Table S2. Search strings.

Database	Search String	Limits
Embase	schizophrenia AND (naloxone OR naltrexone OR buprenorphine OR nalmefene) AND english:la AND [1970-2019]/py	english:la AND [1970-2019]/py
Ovid Medline and PsychINFO	('schizophrenia'/exp or schizophrenia.mp.) and ('naloxone'/exp or naloxone.mp. or 'naltrexone'/exp or naltrexone.mp. or 'buprenorphine'/exp or buprenorphine.mp. or 'nalmefene'/exp or nalmefene.mp.) [mp=ti, ab, ot, nm, hw, fx, kf, ox, px, rx, ui, an, sy, tc, id, tm]	english language to yr="1970 - 2019"
Scopus	ALL ("schizophrenia") AND ALL ("naloxone" OR "naltrexone" OR "buprenorphine" OR "nalmefene") AND PUBYEAR > 1970 AND (LIMIT-TO (LANGUAGE , "English"))	PUBYEAR > 1970 AND (LIMIT-TO (LANGUAGE , "English"))
Pubmed	(schizophrenia) AND (naloxone OR naltrexone OR buprenorphine OR nalmefene)	("1970"[Date - Publication] : "2019"[Date - Publication]) AND English[Language]
Web of Science	(ALL=((schizophrenia) AND (naloxone OR naltrexone OR buprenorphine OR nalmefene))) AND LANGUAGE:(English) Timespan: 1970-2019. Indexes: SCI-EXPANDED, SSCI, A&HCI, CPCI-S, CPCI-SSH, BKCI-S, BKCI-SSH, ESCI, CCR-EXPANDED, IC.	LANGUAGE:(English) Timespan: 1970-2019
Cochrane database CENTRAL	(schizophrenia):ti,ab,kw AND (naloxone OR naltrexone OR buprenorphine OR nalmefene):ti,ab,kw	1970 to 2019 and English
Google Scholar	(schizophrenia) AND (naloxone OR naltrexone OR buprenorphine OR nalmefene)	1970 to 2019 and exclude patents

Supplemental Table S3. Pearson R correlation coefficients.

**Study**	Pearson R	Pearson R for drug	Pearson R for placebo
Kurland et al., 1977 [45]	0.204980015	-0.31622777	-
Emrich et al., 1977 [16]	0.318145372	-	-
Davis et al., 1977 [46]	0.618346942	-	-
Davis et al., 1977	0.275680975	-	-
Davis et al., 1977	0.821041999	-	-
Davis et al., 1977	0.105457686	-	-
Davis et al., 1977	-0.015857043	-	-
Davis et al., 1977	-0.146813945	-	-
Davis et al., 1977	0.4	-	-
Davis et al., 1977	0.323654239	-	-
Janowsky et al., 1977 [47]	-0.094693991	0.86463109	0.830864
Janowsky et al., 1977	0.187862928	0.90150376	0.343867
Watson et al., 1978 [48]	0.075181197	0.27857625	0.731389
Watson et al., 1978	-0.255932724	0.43852363	0.467707

Emrich et al., 1980 [18]	0.5936434	-	-
Gitlin et al., 1981 [49]	0.656166044	0.93392421	0.672104
Berger et al., 1981 [50]	0.188759388	0.22775307	0.706184
Rapaport et al., 1993 [51]	0.661095363	0.94537816	0.907959
Rapaport et al., 1993	0.523264658	0.96440877	0.915396
Rapaport et al., 1993	0.898132069	0.84669457	0.929819
Rapaport et al., 1993	0.635965793	0.64789844	0.654303

Supplemental Table S4. Diagnostic criteria and penalty weights

**Diagnostic Criteria**	**Penalty Weight**
DSM-II	2.0
ICD-9	0.5
Feighner's criteria	0.4
RDC	0.3
DSM-III	0.2
DSM-III-R	0.1
DSM-IV	0.05
DSM-IV-TR	0

Supplemental Table S5. Scales and penalty weights.

**Scales included**	**Penalty weight**
BPRS, PANSS, SAPS, SANS	0
IMPS, NIMH	0.1
CPRS, NOISE	0.2
Bunney Hamburg, VBS, CPT, CGI	0.3

“Self-rated,” “Custom”	0.4

Supplemental Table S6. Characteristics of included participants.

	**Total**	**Known**	**Unknown**
N by gender	434 patients	291m, 123f	20
Avg years of illness	10.38 years	163 patients	271
Avg Age	37.71 years	299 patients	135 patients
Number of patients with active hallucinations at baseline	180	180	254

Supplemental Table S7. Characteristics of included studies.

Study, Country	Drug	AVG daily dose mg (route)	Diagnostic criteria	n by gender	Age range years (avg)	Range of years of illness (avg)	Antipsychotic status	AVG Chlorpromazine Equivalents	Severity at baseline	Design (order) Randomized	Outcome scales reported with sufficient data to analyze	Time points m and (test days)	Summary of findings and (direction of effect)
Gunne et al., 1977 [52] Sweden	naloxone	0.4 (i.v.)	DSM-2	6 (4m 2f)	25-45 (33)	1-23 (NS)	medicated	NS	4 constant hallucinations 2 no hallucinations	SBPCC (NS)	Custom scale Hallucination reduction	90 (1)	Reduced or eliminated auditory hallucinations in 4 patients with prior hallucinations. No effect in 2 patients without prior hallucinations (drug)
Volavka et al., 1977 [53] USA	naloxone	0.4 (i.v.)	Feighner's criteria	7 (6f 1m)	24-50 (NS)	4-30 (NS)	medicated	NS	active hallucinations	DBPCC (AB/BA) "Semirandomized"	BPRS TOTAL excluding Hallucinatory behavior, BPRS Hallucinations, BPRS Unusual thought content	0, 5, 30,60 ,120, 180,240,1440 (1)	"No average difference between naloxone and placebo was seen. Two patients reported a decrease of hallucinations after having been given placebo. However, two other patients showed a slight reduction of hallucinations after naloxone without any

													response to placebo.” (placebo)
Kurland et al., 1977 [45] USA	naloxone	0.8 (i.v.)	DSM-2	12 (7m 5f)	25-50 (41.5)	>3 (NS)	medicated	NS	active hallucinations	DBPCC (AB/BA) Randomized	Custom scale Hallucination reduction	60 (1)	“Of 8 patients who had hallucinatory symptoms before naloxone treatment, only 2 showed a complete disappearance of symptoms following subsequent naloxone injections. Correspondingly, of 12 patients who had hallucinatory symptoms before placebo administration, 5 showed no symptoms following treatment." (placebo)
Emrich et al., 1977 [16] Germany	naloxone	3.58 (i.v.)	ICD-9	20 (9m 11f)	23-68 (NS)	0.5-23 (NS)	unmedicated	0	active hallucinations	DBPCC NS NS	IMPS, VBS	45,60, 120,160, 240,320 (1)	Statistically improved psychotic symptoms especially hallucinations within 2-7 hours (drug)

Davis et al., 1977 [46] USA	naloxone	1.88 (i.v.)	RDC	14 (9m 5f)	18-53 (25)	<0.5 – 23 (7)	mixed	417	NS	DBPCC NS Randomized	BPRS Hallucinations, BPRS Mannerisms and posturing, BPRS Conceptual disorganization, BPRS Unusual thought content, BPRS Psychosis,	60 (1)	Statistically significant improvement in unusual thought content and a trend towards improvement in the other symptoms except mannerisms and posturing "Although 10 of 14 patients had a slight decrease in scores when the eight rating measure were combined this did not achieve statistical significance." (drug)
Janowsky et al., 1977 [47] USA	naloxone	1.2 (i.v.)	DSM-2	8m	19-48 (30)	NS (NS)	medicated	2050	active hallucinations	DBPCC NS NS	BPRS Hallucinations, BPRS Unusual thought content	37.5 (1)	"Analysis of variance of all individual BPRS items and total BPRS scores revealed no significant or near significant differences between placebo and active naloxone trials."

													(Analysis of Table 2 patient level data shows an effect size of 0)
Watson et al., 1978 (single blind) [48] USA	nalox one	10 (i.v.)	Feighne r's criteria	2m	32-33 (32.5)	NS (NS)	unmedicat ed	NS	active hallucin ations	SBPCC NS NS	NIMH Hallucinati ons	97.5 (1)	Improvement in hallucinations in 2 patients compared to placebo. No stats performed. (drug)
Watson et al., 1978 (double blind) [48] USA	nalox one	10 (i.v.)	Feighne r's criteria	9m	22-47 (32.3)	NS (NS)	mixed	NS	active hallucin ations	DBPCC NS Randomi zed	NIMH Hallucinati ons, Self-rated Hallucinati ons,	97.5 (1)	Improvement in hallucinations in six patients lasting 6 to 48 hours “Of the nine patients, six reported a clear-cut improvement in hallucination, once showed borderline improvement and two did not improve beyond the placebo effect.” (drug)
Gunne et al., 1979 [54] Sweden	naltre xone	100 (oral)	DSM-2	10 (7m 3f)	21-57 (25)	1-34 (5.5)	medicated	NS	active hallucin ations	DBPCC AB/BA NS	CPRS TOTAL	NA (14)	“The mean response to naltrexone was negative” examination of figure 50.4 shows that

													the direction of effect favors placebo (placebo)
Lipinski et al., 1979 [55] USA	naloxone	1.6 (i.v.)	Feighner's criteria	9 (8m + 1f)	19-52 (28)	NS (NS)	mixed	1312.5	minimal ratings of hallucinations	DBPCC (AB/BA) Randomized	BPRS TOTAL,	15,75 (1)	"We conclude that in this study no clear effect of naloxone at the dose used could be found on general or specific psychopathology in schizophrenia" (One timepoint favored drug and one favored placebo)
Emrich et al., 1979 [17] Germany	naloxone	24.8 (i.v.)	ICD-9	20 (NS)	NS	NS (NS)	unmedicated	0	active hallucinations	DBPCC NS NS	IMPS, VBS	60, 180, 300, 420 (1)	Significantly reduced psychotic symptoms on IMPS at 3 and 7 hours but not hallucinations on VBS (5 time points favored drug and 3 favored placebo)
Lehman et al., 1979 (single blind) [56]	naloxone	10 (i.v.)	DSM-2	7m	28-60 (41.4)	5-30 (16.8)	unmedicated	0	NS	SBPCC NS NS	BPRS Tension	360 (1)	Statistically significant improvement in tension item on BPRS only.

Canada													(drug)
Lehman et al., 1979 (double blind) [56] Canada	naloxone	10 (i.v.)	DSM-2	5m	NS	NS (NS)	unmedicated	0	NS	DBPPC NS NS	BPRS TOTAL, BPRS Thought disturbance factor, BPRS Hallucinatory Behavior,	360 (1)	Statistically significant improvement in total BPRS, thought disturbance, and hallucinatory behavior. Note: these 5 patients also participated in Lehman's single blind study. (drug)
Emrich et al., 1980 [18] Germany	Naloxone	40 (i.m.)	ICD-9	8(7m 1f)	17-78 (38.25)	NS (NS)	medicated	1017	active hallucinations	DBPCC AB/BA Randomized	IMPS, VBS	120, 360 (1)	Naloxone reduced psychotic symptoms in 4 patients, no effect in 2 patients and worsened symptoms in 1 patient. (drug)
Gitlin et al., 1981 [49] USA	naltrexone	85.7 (Oral)	RDC	8 (4m 4f)	19-32 (24.6)	NS (NS)	mixed	1336	"moderately or severely symptomatic"	DBPCC NS Randomized	BPRS TOTAL, NOSIE	NS (7)	"No significant changes were found between baseline and any naltrexone rating day." (placebo)
Berger et al., 1981 [50] USA	naloxone	10 (i.v.)	RDC	14m	22-59 (35)	NS (NS)	mixed	NS	active hallucinations	DBPCC (AB/BA) Randomized	NIMH Hallucinations	108 (1)	"We found significant reductions in hallucinations." (drug)

Freeman et al., 1981 [57] Scotland	naloxone	1.6 (i.v.)	Feighner's criteria	13 (8m 5f)	25-67 (48)	6-34 (21.3)	medicated	800	active hallucinations	DBPCC NS Randomized	BPRS Hallucinations, Self-rated Hallucinations, Self-rated distress caused by hallucinations,	20, 60 (1)	Overall self-rating of hallucinations showed a non-significant trend toward improvement on naloxone. BPRS did not improve. (drug and placebo depending on scale)
Kleinman et al., 1982 (medicated) [58] USA	naloxone	15 (i.v.)	RDC	8 (7m 1f)	21-45 (NS)	3-27 (10)	medicated	1435	NS	DBPCC NS NS	BPRS Unusual thought content, BPRS Hallucinations, BPRS Conceptual disorganization,	30, 60, 70, 120 (1)	Naloxone significantly improved unusual thought content only and “with regard to auditory hallucinations, several patiennts improved on the BPRS scale, but most showed no effect. Significantly worsened conceptual disorganization. (Unusual though content and hallucinations: drug. Conceptual disorganization: placebo)

Kleinman et al., 1982 (unmedicated) [58] USA	naloxone	15 (i.v.)	RDC	9 (6m 3f)	22-45 (NS)	4-27 (13)	unmedicated	1435	NS	DBPCC NS NS	BPRS TOTAL, BPRS Emotional withdrawal	30 (1)	Naloxone worsened symptoms on BPRS total, hostility, and emotional withdrawal. (placebo)
Pickar et al., 1982 [59] USA, Switzerland, India, Russia, Germany, The Netherlands	naloxone	21.18 (s.c.)	ICD-9	32 (18m 14f)	20-70 (37.96)	NS (NS)	mixed	NS	"prominent psychotic symptoms" in all patients	DBPCC AB/BA Randomized	BPRS TOTAL, BPRS Thought Disorder, BPRS Withdrawal Retardation, BPRS Paranoid suspicion, BPRS Hallucinatory Behavior	30, 60, 180, 184,240, 360 (1)	Group trends toward improvement favors drug (drug)
Naber et al., 1982 [60] Germany	naloxone	10 (s.c.)	ICD-9	12m	NS (36)	NS (13)	medicated	1320	NS	DBPCC NS Randomized	BPRS TOTAL	360 (1,2,3,5) (no day 4)	“No significant drug effect was found in any of these analyses. Changes of time were statistically significant, but the reflected improvement in patients’ psychotic behavior was clinically not relevant.” (Two time points favored drug and two

													favored placebo)
Lo et al., 1983 [61] Hong Kong	Naloxone	0.4 (i.m.)	DSM-3	13 (10m 3f)	34-69 (49)	NS (NS)	medicated	NS	active hallucinations	DBPC (Not crossover) Randomized	BPRS TOTAL	NS (7)	"Of the 7 patients receiving naloxone, three showed signs of improvement. In the saline group, only one case of obvious improvement was observed." Analysis of figure 1 shows overall trend in favor of drug (drug)
Cohen et al., 1985 [62] USA	naloxone	147.2 (i.v.)	DSM-3	3 (2m 1f)	NS (NS)	NS (NS)	unmedicated	0	NS	DBPCC AB/BA Randomized	BPRS TOTAL	198 (1)	Improved BPRS in 3 patients with chronic schizophrenia. No statistics performed (drug)
Schmauss et al., 1987 [19] Germany	Buprenorphine	0.2 (s.i)	DSM-3	10 (3m 7f)	19-43 (NS)	NS (NS)	unmedicated	0		DBPCC AB/BA Randomized	IMPS, VBS	60, 120, 180, 240 (1)	"Buprenorphine had a pronounced antipsychotic effect, which lasted about 4 hours, in seven of 10 patients suffering from schizophrenia. Only in those three patients who

													had residual symptoms was buprenorphine ineffective.” (drug)
Pickar et al., 1989 [63] USA, Russia, India, Holland, Germany	naloxone	22.2	ICD-9	43 (30m 13f)	NS (NS)	NS (NS)	medicated	NS	“Stable clinical condition”	DBPCC (AB/BA) Randomized	BPRS TOTAL	184 (1,2, 3,4)	Both naloxone and placebo showed reduction of hallucinations from baseline. Naloxone was not superior to placebo (5 timepoints favored placebo 3 timepoints favored drug)
Marchesi et al., 1992 [64] Italy	naltrexone	100 (oral)	DSM-3-R	12(9 m 3f)	NS (33.8)	NS (10)	medicated	NS	“negative schizophrenics"	DBPC (not crossover) NS	BPRS TOTAL, BPRS Withdrawal Retardation, BPRS Hostile Suspiciousness, BPRS Schizophrenia Restricted, BPRS Negative schizophrenic symptoms	NS (7,14)	The drug group showed a non-significant trend towards improvement compared to placebo. (drug)
Rapaport et al., 1993 [51] USA	nalmefene	79 (oral)	DSM-3-R	11(8 m 3f)	18-36 (26)	NS (NS)	medicated	1290	NS	DBPCC ABA	BPRS TOTAL, BPRS Thinking	NS (36.7)	Trend towards improvement in BPRS

										all patients ABA	Disturbanc e,		TOTAL and thinking disturbance. (drug)
Marchesi et al., 1995 [65] Italy	naltre xone	100 (oral)	DSM-3-R	18(13 m 5f)	24-56 (35)	3-36 (11.5	medicated	NS	NS	DBPC (not crossove r) NS	BPRS TOTAL, BPRS Thinking Disturbanc e, BPRS Withdrawa l Retardatio n, BPRS Hostile Suspiciou sness,	NS (7, 14)	Non-significant trend towards improvement vs placebo (drug)
Petrakis et al., 2004 [66] USA	naltre xone	50 (oral)	DSM-4	31m	NS (46)	NS (NS)	medicated	NS	NS	DBPC (Not crossove r) Randomi zed	PANSS General psychopat hology, PANSS positive, PANSS negative	84	Psychopathol ogy was not a main outcome of this study. (PANSS General and PANSS positive favored placebo PANSS negative favored drug)
Tatari et al., 2014 [67] Iran	naltre xone	100 (oral)	DSM-4-TR	60 (33m 27f)	18-55 (40.6)	NS (7.7)	medicated	200	NS	DBPC (Not crossove r) Randomi zed	SANS TOTAL, SANS Affective flattening, SANS Poverty of speech, SANS Apathy, SANS Anhedonia , SANS Inattentive	84	Both SAPS and SANS showed statistically significant improvement on naltrexone. (drug)

											ness, SAPS TOTAL, SAPS Hallucinations, SAPS Delusions, SAPS Bizarre behavior, SAPS Thought disorder		

Supplemental Table S8. Characteristics of unique excluded studies.

**Excluded studies**													
**Study, Country**	**Drug**	**AVG daily dose mg (route)**	**Diagnostic criteria**	**n by gender**	**Age range years (avg)**	**Range of years of illness (avg)**	**Antipsychotic status**	**Severity of illness at baseline**	**Design**	**Outcome scales reported**	**Time points m and (test days)**	**Summary of findings and (direction of effect)**	**Reason for exclusion**
Simpson et al., 1977 [68] USA	naltrexone	100-800 (oral)	NS	6 (3m 3f)	32-54 (44.2)	2-29 (13.3)	NS	NS	SBPCC (ABA)	BPRS, CGI, NOSIE	(1,14,28,42,56,70,84,98)	Patients got worse from baseline on placebo and better on drug compared to placebo (but still worse compared to baseline) and then worse again on placebo. The patients spent 4 weeks on placebo and 8 weeks on increasing doses of drug and then another 2 weeks on placebo. From table I it would seem that the	Unable to determine direction of effect size.

												effect size is in favor of drug. However, 2 patients (30%) of the trial dropped out near the end without participating in the final placebo period and it is not clear if part of their data is mixed into table I or not. Since we don't know which patients table I refers to, the direction of effect is ambiguous. (NA)	
Mielke et al., 1977 [69] USA	naltre xone	50-250 (oral)	ICD-9	5 (1m 4f)	34-47 (NS)	5-26 (NS)	unmedic ated	active halluci nations	SB no placebo control	BPRS, CGI, NOSIE	(1,14,2 8,42)	"The findings of this exploratory uncontrolled clinical trial…Two subjects were dropped…becaus e of severe exacerbation of the psychotic process…Three patients without evidence of symptom remission." (NA)	No placebo control.
Kline et al., 1977 [70] USA	beta endor phin and nalox one	0.4mg naloxo ne and 1.5mg beta endorp hin (i.v.)	NS	3m	34-41 (36.6)	NS (NS)	medicate d	NS	SBPCC	Self reported	60,120 ,180 (1)	Some "increased difficulty conceptualizing" after naloxone and beta endorphin. Some reduction in hallucinations after beta endorphin. No scales used to	Mixed drugs

												measure results and no statistics. (NA)	
Orr & Openheimer 1978 [71] England	naloxone	0.4 (i.v.)	NS	1f	28	10	medicated	active hallucinations	SBPCC	patient self-reported	(15-180) (1)	"Hallucinations were less intense; at 15 minutes they had disappeared and she became mildly euphoric. The mood elevation lasted for two hours, and the hallucinations had returned to their usual intensity after three hours….No effects were observed after isotonic saline..." (drug)	Case report n of 1
Gitlin & Rosenblatt 1978 [72] USA	naltrexone	50-100 (oral)	RDC	3 (2m 1f)	NS (NS)	NS (NS)	mixed	NS	SB no placebo control	BPRS and CGI	NS (7,14)	"no therapeutic effect was demonstrated" 2 patients with schizophrenia developed nausea, vomiting, abdominal pain, after a test dose of 20mg naltrexone." however no statistics, direction of effect, magnitude of change of scores, or any data at all was reported on BPRS scores for drug or placebo making it impossible to	Unable to determine direction of effect size

												determine the direction of effect on the BPRS endpoint. (NA)	
Akil et al., 1978 [73] USA	naltrexone	250 (oral)	DSM-2	NS (NS)	NS (NS)	NS (NS)	NS	NS	Open label no placebo control	NIMH	NS	Very little information available: "We have noted a mild improvement in these subjects based on overall ratings and several subcategories of the NIMH rating scale." (drug)	No placebo control
Dysken et al., 1978 [74] USA	naloxone followed by sodium amylobarbital	20mg naloxone and 250mg sodium amylobarbital (i.v.)	NS	1m	25	NS (NS)	NS	catatonia	SB case report	no scale used	NS (1)	catatonia improved with sodium amylobarbital. (NA)	Case report n of 1, and mixed drugs.
Abrams et al., 1978 [75] USA	naloxone and sodium amylobarbital		NS	1m	33	2	medicated	catatonia	SB case report	no scale used	NS (1)	No effect of saline or naloxone on catatonia. (NA)	Case report n of 1, and mixed drugs.
Schenk et al., 1978 [76] Germany	naloxone	various (3.2-380mg)	ICD-9	11(8m 3f)	19-66 (33.18)	NS (NS)	unmedicated	NS	SB no placebo control	CGI respiratory rate and EEG	30-90 (3-21)	8 patients with catatonia improved and 2 with hallucinations possibly improved. (NA)	No placebo control.

Davis et al., 1979 [77] USA	naltrexone	NS (oral)	RDC	2 (NA)	NS (NS)	NS (NS)	NS	NS	SB pilot study	BPRS	NS	Open pilot study briefly mentioned as a side note within Davis et al., 1979 with no detail given except no improvement. (NA)	No placebo control.
Gunne et al., 1979 [54] Sweden	naloxone	0.8mg	DSM-2	10	18-52 (33.5)	1-20 (9)	medicated	active hallucinations	DBPCC AB/BA4	patient self rating	240 (1,2,3, 4,5)	"The i.v. injections…eliminated the hallucinations in only one case on three separate days, in another case all injections caused a cessation of hallucinosis…in the other eight patients the reaction pattern was ambiguous." (NA)	Unable to determine direction of effect size
Watson et al., 1979 [78] USA	naltrexone	250-800	NS	4	NS (NS)	NS (NS)	NA	NA	DBPCC	NIMH	NA	Little information on the results available: "The condition of two open-label subjects seemed substantially improved by the 250 mg/day for two weeks. When a double-blind · protocol was instituted, the condition of a chronic schizophrenic subject seemed slightly improved (a decrease of two points on the	Unable to determine direction of effect size

												NIMH global illness scale) on 250 mg/day. The second double-blind subject also received 250 mg/day. His condition too, seemed improved when contrasted to placebo (down 1.5 points on the NIMH global illness scale). Yet, when that same subject received 800 mg/day in a randomized double-blind protocol, there was no detectable clinical improvement during the placebo period."<br>(NA)	
Ragheb et al., 1980 [79] USA	naltrexone	50-300 (oral)	DSM-2	5 (4m 1f)	24-56 (38)	NS (NS)	mixed	active hallucinations	Open Pilot study with 1 week placebo washout	BPRS and CGI	(4,7,10,14,21)	One patient "much worse", one patient "minimally worse", one patient "no change", one patient "much improved", one patient "very much improved."<br>(NA)	No placebo control
Sethi et al., 1981 [80] India	naloxone	0.3mg/kg (i.v.)	ICD-9	12 (9m 3f)	18-45<br><br>(NS)		unmedicated		DBPCC AB/BA	BPRS and CGI	30, 60, 120, 180, 240,	More frequency increase in conceptual disorganization at 4 hours. In terms	Data already included in Pickar 1982.

											300, 360 (1)	of overall effect: "Besides, it was observed that three of our patients showed a relatively favourable response to the first injection of naloxone but during the 5 subsequent injections of either naloxone or placebo, the response did not seem to have a significantly specific relationship with the type of substance." unable to determine effect size due to reporting only the frequency of responders but not the magnitude of the response compared to placebo. (NA)	
Jorgensen et al., 1982 [81] Norway	naloxone	0.8 (i.v.)	DSM-3 and Feighner's criteria	1f	28	10	unmedicated	active hallucinations	DB case report AB/BB/ AA/BA/ AA/BB	CPRS	60 (1,2,3)	"The treatment with naloxone caused a marked and statistically significant reduction of symptoms. This reduction was apparent in all the items which contributed to the total score...Saline	Case report n of 1

												did not reduce symptoms significantly.” (drug)	
Naber & Leibl 1983 [82] Germany	nalox one	20 (s.c.)	ICD-9	6(3m 3f)	19-47 (29.8)	0-25 (7.5)	mixed	NS	DBPCC	BPRS AB/BA	30, 60, 180, 300, 420 (1,2,3, 4)	No significant effect and unable to determine direction of effect. While figure 1 appears to suggest that the direction was in favor of naloxone there is no baseline provided so a definitive determination of direction of effect size is not possible. (NA)	Unable to determine direction of effect size
Mongan & Callaway 1990 [83] USA	Bupre norphi ne	0.2 (s.i.)	DSM-3R	2	NS (NS)	NS (NS)	NS	NS	Open label and DBPCC	“Short mood scale, a pain scale and the Profile of Mood Scale (POMS) ”	150 (1)	No scales of psychopathology were used so we were unable to determine any effects on symptoms of schizophrenia. (NA)	Unable to determine direction of effect size
Groves, S. and D.J. Nutt 1991 [84] England	Bupre norphi ne	0.2 (s.i.)	NS	7	NS (NS)	NS (NS)	medicate d	NS	Open label no placebo control	BPRS	60, 120, 180, 240 (1)	"We have discovered that a single dose can have marked beneficial effects on some symptoms, particularly auditory hallucinations, mood and	No placebo control

												hostility...The effect peaked at 1-2h, which parallels the known pharmacokinetics of the drug." (NA)	
Nishikawa et al., 1992 [85] Japan	Naloxone	0.6 (i.v.)	NA	1	54	31	NS	NS	NS	Weight gain due to polydipsia	NS	Naloxone reduced polydipsia. Unable to determine any effect on positive or negative symptoms (no schizophrenia scale used). (NA)	Single patient case report
Becker et al., 1995 [86]	naltrexone	50	DMS-3	7m	(42)	20	medicated	NS	open label pilot study without placebo control	Elgin Behavior Rating Scale	(42)	Trend toward reduction in mannerisms social withdrawal and polydipsia. (NA)	No placebo control
Nishikawa et al., 1996 [87] Japan	Naloxone	0.6 (i.v.)	NA	8(7m 1f)	43-68 (52.5)	20-36 (27.8)	NS	NS	DBPCC AB/BA	Weight gain due to polydipsia	NS	Primary endpoint was weight gain. Unable to determine any effect on positive or negative symptoms (no schizophrenia scale used). (NA)	Unable to determine direction of effect size
Sernyak et al. 1998 [88] USA	naltrexone	200 (oral)	DSM-3	21(15m 6f)	21-57 (33)		medicated		double blind placebo controlled for the first 3 weeks with single blind	BPRS	NS (21,42)	At the half way point, the blind was broken and the drug first arm was not crossed over to placebo. " Fifteen patients received placebo and six received	Change in design midway through the study

									crossover of one arm only for the next 3 weeks AB/BA			naltrexone in the first 3 weeks. No significant effects of naltrexone on total BPRS scores or BPRS subscale scores were observed... Patients who received naltrexone on a single-blind basis at the end of the placebo-controlled trial demonstrated a transient exacerbation in negative symptoms as reflected by the total BPRS score (Figure 1) and the BPRS withdrawal-Retardation subscale score." (No effect size available for the double blind portion, effect favors placebo for the single bind portion in the group that was crossed over)	
Wonodi et al., 2004 [89] USA	Naltrexone	50-200	NA	14 (7m 7f)	47	NA	medicated	“only mild psychotic symptoms at baseline.”	DBPCC	BPRS	(70)	“No significant effect of naltrexone on…BPRS scores…BPRS scores remained unchanged during the naltrexone treatment.” No numerical data on	Unable to determine direction of effect

												BPRS vs placebo reported. (NA)	
Petrakis et al. 2005 [90]	naltrexone or disulfiram or both or placebo	50 (oral)	DSM-4	254 (247 m 7f) with various diagnoses but only 18 had schizophrenia	NS (47)	NS (NS)	mixed	NS	DBPC	Brief Symptoms Inventory (BSI)		Drinking improved. Subjects (of all mixed diagnoses) treated with either disulfiram or naltrexone had an improvement in paranoid ideation on BSI. (NA)	Results were not stratified by diagnosis and included non-schizophrenic patients in analysis
Sardarpour, Arbabi, and Samimi. 2006 [91] Iran	Naltrexone	NS	NS	34	NS (NS)	NS (NS)	medicated	NS	DBPC	PANSS	NS	NS	No English translation available
Batki et al., 2007 [92]	Naltrexone	100-150 (oral)	DSM-4	19 (15m 4f)	NS (43)	NS (NS)	medicated	NS	open label	PANSS and Adiction Severity Index ASI	NS (56)	Improvement in PANSS positive and negative and general psychopathology scores (NA)	No placebo control

Leontieva et al., 2009 [93]	Naltrexone	NS	NS	79	NS (NS)	NS (NS)	medicated	NS	DBPC	PANSS and patient satisfaction survey	84	Overall 22% of providers reported a reduction in PANSS however not clear if this was in relation to placebo or baseline. (NA)	Unable to determine direction of effect size
Vasile et al., 2013 [94]	Naltrexone OR disulfiram	50mg naltrexone or 250mg disulfiram	DSM-4-TR	20 (9m 11f)	40.2	NS	medicated	NS	open label	PANSS and other drug related scales	140	PANSS improved 26% from baseline for both groups. (NA)	No placebo control
Taveira et al., 2013 [95]	Naltrexone	50	DSM-4-TR	30 (20m 10f)	21-55	NS	medicated	Clinically stable	DBPC	Body mass index	84	No scales used to measure symptoms of schizophrenia. (NA)	Unable to determine direction of effect size
Bratu et al., 2014 [96] Romania	Naltrexone or acomprosate or counseling only	50	DSM-4-TR	36 (24m 12f)	NS	NS	medicated	stable	open label	IDTS, GAF, PANSS	168	"PANSS Negative subscale scores decreased most in naltrexone-treated patients but this did not reach significance," (NA)	No placebo control
Tek et al., 2014 [97] USA	Naltrexone	25 (oral)	DSM-4	24f	45.5	NS	medicated	stable	DBPC	PANSS	56	"No differences in psychiatric symptoms" No PANSS data reported. (NA)	Unable to determine direction of effect size

Vasile et al., 2014 [98]	Nalm efene	18 (oral)	DSM-4-TR	22 (10m 12f)	44.2	NS	medicate d	stable	open label	PANSS	168	"At week 24, patients had an overall improved PANSS score of 15.4% reported to baseline." (NA)	No placebo control

Supplemental Table S9. Excluded studies due to duplicate publication of data or review articles.

**Study, Country**	**Reason for Exclusion**
Janowsky et al., 1977 [99]<br>USA	Re-publication of Janowski et al., 1977 [47]
Davis et al., 1977b [100]<br>USA	Re-publication of Davis et al., 1977a [46]
Akil et al., 1978 [73]<br>USA	Earlier publication with less data Watson et al., 1978 [48]
Emrich et al., 1978 [101]	Re-publication of Emrich et al., 1977 [16]
Janowsky et al., 1979 [102]<br>USA	Re-publication of Janowski et al., 1977 [47]
Perez-Cruet et al., 1979 [103]	Re-publication of Volvoka et al., 1977 [53]
Volovka et al., 1979 [104]<br>USA	Narrative review of previous studies
Davis et al., 1979 [77]<br>USA	Re-publication of Davis et al. 1977 [46]

Davis et al., 1979 [105]<br>USA	Narrative review of previous studies
Berger et al., 1979 [106]<br>USA	Re-publication of Watson et al., 1978 [48]
Emrich et al., 1979 [107]<br>Germany	Re-publication of Emrich et al., 1977 [16] and 1979 [17]
Lehman Cooper, Nair and Klein , 1979 (single blind) [108]<br>Canada	Re-publication of Lehman et al., 1979 [56]
Lehman Cooper, Nair and Klein 1979 (double blind) [108]<br>Canada	Re-publication of Lehman et al., 1979 [56]
Verebey et al., 1979 [109]<br>USA	Re-publication of Simpson et al., 1977 [68]<br>USA
Verebey et al., 1979 [110]<br>USA	Re-publication of Simpson et al., 1977 [68]<br>USA
Barchas et al., 1980 [111]	Narrative review of previous studies
Malek-Ahmadi & Callen [112]	Narrative review of previous studies
Lideman et al., 1980 [113]<br>Russia	Already included in Pickar et al., 1982 [59]
Verhoeven et al., 1981 [114]	Already included in Pickar et al., 1982 [59]
Ananth, Nair and Rastogi 1982 [115]	Narrative review of previous studies
Mielke 1982 [116]	Narrative review of previous studies
Verhoeven et al., 1982 [117]	Already included in Pickar et al., 1982 [59]

The Netherlands	
Sethi et al., 1981 [80]<br>India	Most of the data was already included in Pickar et al., 1982 [59] AND this study met criteria for unable to determine direction of effect size
Volovka et al., 1982 [118]<br>USA	Narrative review of previous studies
Handal, Schauben, and Salamone 1982 [119]	Narrative review of previous studies
Mueser & Dysken 1983 [120]<br>USA	Narrative review of previous studies
Lideman et al., 1984 [121]<br>Russia	Already included in Pickar et al., 1982 [59]
Verhoeven et al., 1984 [122]<br>The Netherlands	Already included in Pickar et al., 1989 [63]
Goldbloom 1984 [123]<br>USA	Narrative review of previous studies
McNicholas & Martin 1984 [124]<br>USA	Narrative review of previous studies
Nemeroff & Bissette 1986 [125]<br>USA	Narrative review of previous studies
Budd 1987 [126]<br>USA	Narrative review of previous studies

Marchesi et al., 1991 [127]<br>Italy	A poster of preliminary results later published as Marchesi et al., 1992 [64]
Marchesi et al., 1992 [128]<br>Italy	(also poster with the same data as Marchesi et al., 1992 [64])
Welch & Thompson 1994 [129]<br>USA	Narrative review of previous studies
Modesto-Lowe & Van Kirk 2002 [130]<br>USA	Narrative review of previous studies
Petrakis et al., 2006 [131]	Same data as Petrakis et al. 2005 [90]
Ralevski et al., 2006 [132]	in regards to PANSS changes This is re-publication of data from Petrakis et al., 2004[66]
Batki et al., 2009 [133]	This is a review of opioid antagonists in dual diagnosed patients with schizophrenia
Tatari et al., 2015 [134]<br>Iran	Re-publication of Tatari et al., 2014 [67]
Azorin et al., 2015 [135]	This is a narrative review of opioid antagonists in dual diagnosed patients with schizophrenia
Bennette, Bradshaw & Catalano 2016 [136]	This is a review of opioid antagonists in dual diagnosed patients with schizophrenia

Sawicka and Tracy 2017 [137]	This is a systematic review of naltrexone in dual diagnosed patients with alcohol use disorder and schizophrenia

Supplemental Table S10. Results of naloxone only.

Scale	Weighting	Model	G	P	k	CI	I^2	P (I^2)
All Scales	unweighted	Random	0.15188	0.11431	16	-0.041191 - 0.34494	0.60412	0.00093669
		Bootstrap	0.19216	0.036169	22	0.016443 - 0.34334	-	-
	weighted	Random	0.15633	0.11431	16	-0.041191 - 0.34494	0.60437	0.00092892
		Bootstrap	0.19945	0.01949	22	0.038369 - 0.35839	-	-
Total scales	unweighted	Random	0.084636	0.3979	9	-0.13391 - 0.30318	0.428	0.082129
		Bootstrap	0.16348	0.094226	12	-0.039766 - 0.5405	-	-
	weighted	Random	0.077423	0.3979	8	-0.13391 - 0.30318	0.42834	0.081913
		Bootstrap	0.15784	0.13211	11	-0.058486 - 0.52883	-	-
All positive	unweighted	Random	0.23335	0.0362	12	0.017976 - 0.44873	0.59131	0.0047324
		Bootstrap	0.24571	0.026458	16	0.038665 - 0.40059	-	-
	weighted	Random	0.2532	0.0362	12	0.017976 - 0.44873	0.59295	0.0045572
		Bootstrap	0.26285	0.0056775	16	0.10102 - 0.41688	-	-
Hallucinations	unweighted	Random	0.22301	0.16056	8	-0.11301 - 0.55904	0.71488	0.00091117

		Bootstrap	0.24902	0.086112	13	-0.058286 - 0.46668	-	-
	weighted	Random	0.24864	0.16056	8	-0.11301 - 0.55904	0.71636	0.00086481
		Bootstrap	0.2687	0.030082	13	0.03483 - 0.49211	-	-
Delusions	unweighted	Random	-	-	-	-	-	-
		Bootstrap	0.38198	0.00017744	6	0 - 1.0037	-	-
	weighted	Random	-	-	-	-	-	-
		Bootstrap	0.43357	0.00010922	6	0 - 1.0037	-	-

Supplemental Table S11 results of naltrexone only.

Scale	Weighting	Model	G	P	k	CI	I^2	P (I^2)
All Scales	unweighted	Random	-	-	-	-	-	-
		Bootstrap	0.58473	0.21746	6	-0.11738 - 1.8727	-	-
	weighted	Random	-	-	-	-	-	-
		Bootstrap	0.61086	0.19074	6	-0.10483 - 1.8727	-	-

Supplemental Table S12 results of subgroup analysis one hour only.

Scale	Weighting	Model	G	P	k	CI	I^2	P (I^2)
All Scales	unweighted	Random	0.15854	0.30031	7	-0.18374	0.6608	0.0070594

						- 0.50082		
		Bootstrap	0.15854	0.29925	9	- 0.15656 - 0.45586	-	-
	weighted	Random	0.214	0.30031	7	- 0.18374 - 0.50082	0.66704	0.0061816
		Bootstrap	0.214	0.023311	9	0.03159 - 0.65767	-	-
All positive	unweighted	Random	0.061315	0.76395	6	- 0.43567 - 0.5583	0.78177	0.00035092
		Bootstrap	0.061315	0.75149	8	- 0.33085 - 0.54371	-	-
	weighted	Random	0.12316	0.76395	6	- 0.43567 - 0.5583	0.78513	0.00029969
		Bootstrap	0.12316	0.48423	8	- 0.26038 - 0.64928	-	-

Supplemental Table S13 results of subgroup analysis three to seven hours only.

Scale	Weighting	Model	G	P	k	CI	I^2	P (I^2)
All Scales	unweighted	Random	0.18639	0.10168	7	- 0.049777 - 0.42255	0.36584	0.14925
		Bootstrap	0.23067	0.018115	11	0.046839 - 0.43862	-	-
	weighted	Random	0.18764	0.10168	7	- 0.049777 - 0.42255	0.36585	0.14924
		Bootstrap	0.23123	0.01496	11	0.05489 - 0.4482	-	-
Total scales	unweighted	Random	0.18639	0.10168	7	- 0.049777 - 0.42255	0.36584	0.14925

		Bootstrap	0.23867	0.017016	10	0.048673 - 0.50315	-	-
	weighted	Random	0.18764	0.10168	7	-0.049777 - 0.42255	0.36585	0.14924
		Bootstrap	0.23937	0.013547	10	0.059173 - 0.52328		
All positive	unweighted	Random	-	-	-	-	-	-
		Bootstrap	0.23898	0.17818	7	-0.10916 - 0.54298		
	weighted	Random	-	-	-	-	-	-
		Bootstrap	0.23732	0.16743	10	-0.11807 - 0.55014		

Supplemental Table S14 results of moderator analysis of chlorpromazine equivalents.

Scale	Weighting	Model	Beta	P	k	CI
All Scales	unweighted	Random	-0.00045125	0.00010414	13	-0.0013271 - -0.00014289
		Bootstrap	-	-	-	-
	weighted	Random	-0.00051844	5.2056e-05	13	-0.0014261 - -0.00015385
		Bootstrap	-	-	-	-

Supplemental References

1. Borenstein, M., H. Cooper, L. Hedges, and J. Valentine, *Effect sizes for continuous data. The handbook of research synthesis and meta-analysis*, 2009. **2**: p. 221-235.
2. Association, A.P. and Statistics, *DSM-II diagnostic and statistical manual of mental disorders*. 1968: American Psychiatric Association.

3. Feighner, J.P., E. Robins, S.B. Guze, R.A. Woodruff, G. Winokur, and R. Munoz, *Diagnostic criteria for use in psychiatric research.* Archives of general psychiatry, 1972. **26**(1): p. 57-63.
4. Spitzer, R.L., J. Endicott, and E. Robins, *Research diagnostic criteria: rationale and reliability.* Archives of general psychiatry, 1978. **35**(6): p. 773-782.
5. Association, A.P., *DSM-III: Diagnostic and Statistical Manual of Mental Disorders, 3rd Edition*. 1980, The American Psychiatric Association.
6. Association, A.P., *Diagnostic and Statistical Manual of Mental Health Disorders (DSM-III-R)*. 1987: American Psychiatric Association.
7. Association, A.P., *DSM-IV: Diagnostic and Statistical Manual of Mental Disorders, 4th Edition*. 1994: American Psychiatric Association.
8. Association, T.A.P., *DSM-IV: Diagnostic and Statistical Manual of Mental Disorders, 4th Edition Text Revision*. 2000: The American Psychiatric Association.
9. Norman, R.M., A.K. Malla, L. Cortese, and F. Diaz, *A study of the interrelationship between and comparative interrater reliability of the SAPS, SANS and PANSS.* Schizophrenia Research, 1996. **19**(1): p. 73-85.
10. Lyne, J.P., A. Kinsella, and B. O'Donoghue, *Can we combine symptom scales for collaborative research projects?* Journal of Psychiatric Research, 2012. **46**(2): p. 233-238.
11. Bell, M., R. Milstein, J. Beam-Goulet, P. Lysaker, and D. Cicchetti, *The Positive and Negative Syndrome Scale and the Brief Psychiatric Rating Scale: reliability, comparability, and predictive validity.* Journal of Nervous and Mental Disease, 1992.
12. Daniel, D.G., *Issues in selection of instruments to measure negative symptoms.* Schizophrenia research, 2013. **150**(2-3): p. 343-345.
13. Lorr, M., *Inpatient multidimensional psychiatric scale (IMPS)*. 1962: Consulting Psychologists Press.
14. Cairns, V., T. Faltermaier, H.-U. Wittchen, H. Dilling, W. Mombour, and D. von Zerssen, *Some problems concerning the reliability and structure of the scales in the inpatient multidimensional psychiatric scale (IMPS).* Archiv für Psychiatrie und Nervenkrankheiten, 1982. **232**(5): p. 395-406.
15. Åsberg, M.E., C.E. Perris, D.E. Schalling, and G.E. Sedvall, *CPRS: Development and applications of a psychiatric rating scale.* Acta Psychiatrica Scandinavica, 1978.
16. Emrich, H., C. Cording, S. Piree, A. Kölling, D. Zerssen, and A. Herz, *Indication of an antipsychotic action of the opiate antagonist naloxone.* Pharmacopsychiatry, 1977. **10**(05): p. 265-270.
17. Emrich, H., V. Höllt, H. Laspe, M. Fischler, H. Heinemann, W. Kissling, et al., *Studies on a possible pathological significance of endorphins in psychiatric disorders.* Neuropsychopharmacology, 1979: p. 527-534.
18. Emrich, H., M. Bergmann, W. Kissling, W. Schmid, D. Zerssen, A.H.E. Costa, et al., *Neural Peptides and Neuronal Communication*. 1980, Raven Press, New York.
19. Schmauss, C., A. Yassouridis, and H.M. Emrich, *Antipsychotic effect of buprenorphine in schizophrenia.* The American Journal of Psychiatry, 1987. **144**(10): p. 1340-1342.
20. Higgins, J.P., J.A. Sterne, J. Savovic, M.J. Page, A. Hróbjartsson, I. Boutron, et al., *A revised tool for assessing risk of bias in randomized trials.* Cochrane database of systematic reviews, 2016. **10**(Suppl 1): p. 29-31.
21. Higgins, J. and S. Green, *16.4. 3. Assessing risk of bias in cross-over trials.* Cochrane Handbook for Systematic Reviews of Interventions Version, 2011. **5**(0).
22. Pfeiffer, A., V. Brantl, A. Herz, and H.M. Emrich, *Psychotomimesis mediated by k opiate receptors.* Science, 1986. **233**(4765): p. 774-776.
23. Giuffra, M., M. Mouradian, T. Davis, J. Ownby, and T. Chase, *Dynorphin agonist therapy of Parkinson's disease.* Clinical neuropharmacology, 1993. **16**(5): p. 444-447.

24. Reece, P.A., A.J. Sedman, S. Rose, D.S. Wright, R. Dawkins, and R. Rajagopalan, *Diuretic effects, pharmacokinetics, and safety of a new centrally acting kappa-opioid agonist (CI-977) in humans.* The Journal of Clinical Pharmacology, 1994. **34**(11): p. 1126-1132.
25. Gadano, A., R. Moreau, F. Pessione, C. Trombino, N. Giuily, P. Sinnassamy, et al., *Aquaretic effects of niravoline, a κ-opioid agonist, in patients with cirrhosis.* Journal of hepatology, 2000. **32**(1): p. 38-42.
26. Dortch-Carnes, J. and D.E. Potter, *Bremazocine: A κ-Opioid Agonist with Potent Analgesic and Other Pharmacologic Properties.* CNS Drug Reviews, 2005. **11**(2): p. 195-212.
27. Roth, B.L., K. Baner, R. Westkaemper, D. Siebert, K.C. Rice, S. Steinberg, et al., *Salvinorin A: a potent naturally occurring nonnitrogenous κ opioid selective agonist.* Proceedings of the National Academy of Sciences, 2002. **99**(18): p. 11934-11939.
28. MacLean, K.A., M.W. Johnson, C.J. Reissig, T.E. Prisinzano, and R.R. Griffiths, *Dose-related effects of salvinorin A in humans: dissociative, hallucinogenic, and memory effects.* Psychopharmacology, 2013. **226**(2): p. 381-392.
29. Maqueda, A.E., M. Valle, P.H. Addy, R.M. Antonijoan, M. Puntes, J. Coimbra, et al., *Naltrexone but not ketanserin antagonizes the subjective, cardiovascular, and neuroendocrine effects of salvinorin-A in humans.* International Journal of Neuropsychopharmacology, 2016. **19**(7): p. pyw016.
30. Resnick, R.B., M. Fink, and A.M. Freedman, *Cyclazocine treatment of opiate dependence: A progress report.* Comprehensive psychiatry, 1971. **12**(6): p. 491-502.
31. Hanlon, T.E., O.L. McCabe, C. Savage, and A.A. Kurland, *A controlled comparison of cyclazocine and naloxone treatment of the paroled narcotic addict.* International pharmacopsychiatry, 1975. **10**: p. 240-250.
32. Kumor, K.M., C.A. Haertzen, R.E. Johnson, T. Kocher, and D. Jasinski, *Human psychopharmacology of ketocyclazocine as compared with cyclazocine, morphine and placebo.* Journal of Pharmacology and Experimental Therapeutics, 1986. **238**(3): p. 960-968.
33. Jasinski, D.R., W.R. Martin, and J.D. Sapira, *Antagonism of the subjective, behavioral, pupillary, and respiratory depressant effects of cyclazocine by naloxone.* Clinical Pharmacology & Therapeutics, 1968. **9**(2): p. 215-222.
34. Ashok, A.H., J. Myers, T.R. Marques, E.A. Rabiner, and O.D. Howes, *Reduced mu opioid receptor availability in schizophrenia revealed with [11 C]-carfentanil positron emission tomographic Imaging.* Nature communications, 2019. **10**(1): p. 1-9.
35. Abdallah, K. and L. Gendron, *The delta opioid receptor in pain control*, in *Delta Opioid Receptor Pharmacology and Therapeutic Applications*. 2017, Springer. p. 147-177.
36. Pierre, F., M. Ugur, F. Faivre, S. Doridot, P. Veinante, and D. Massotte, *Morphine-dependent and abstinent mice are characterized by a broader distribution of the neurons co-expressing mu and delta opioid receptors.* Neuropharmacology, 2019.
37. Hirose, N., K. Murakawa, K. Takada, Y. Oi, T. Suzuki, H. Nagase, et al., *Interactions among mu-and delta-opioid receptors, especially putative delta1-and delta2-opioid receptors, promote dopamine release in the nucleus accumbens.* Neuroscience, 2005. **135**(1): p. 213-225.
38. Laughren, T. and R. Levin, *Food and Drug Administration commentary on methodological issues in negative symptom trials.* Schizophrenia bulletin, 2011. **37**(2): p. 255-256.
39. Marder, S.R., D.G. Daniel, L. Alphs, A.G. Awad, and R.S. Keefe, *Methodological issues in negative symptom trials.* Schizophrenia bulletin, 2011. **37**(2): p. 250-254.

40. Marder, S.R., L. Alphs, I.-G. Anghelescu, C. Arango, T.R. Barnes, I. Caers, et al., *Issues and perspectives in designing clinical trials for negative symptoms in schizophrenia.* Schizophrenia research, 2013. **150**(2-3): p. 328-333.
41. Wang, D.S., G. Sternbach, and J. Varon, *Nalmefene: a long-acting opioid antagonist. Clinical applications in emergency medicine.* The Journal of emergency medicine, 1998. **16**(3): p. 471-475.
42. Verebey, K., J. Volavka, S.J. Mule, and R. Resnick, *Naltrexone: disposition, metabolism, and effects after acute and chronic dosing.* Clinical Pharmacology & Therapeutics, 1976. **20**(3): p. 315-328.
43. Lavonas, E.J., S.G. Severtson, E.M. Martinez, B. Bucher-Bartelson, M.-C. Le Lait, J.L. Green, et al., *Abuse and diversion of buprenorphine sublingual tablets and film.* Journal of substance abuse treatment, 2014. **47**(1): p. 27-34.
44. Gray, R., A. Ferry, and P. Jauhar, *Emergence of buprenorphine dependence.* British Journal of Addiction, 1989. **84**(11): p. 1373-1374.
45. Kurland, A.A., O.L. McCabe, T.E. Hanlon, and D. Sullivan, *The treatment of perceptual disturbances in schizophrenia with naloxone hydrochloride.* Am J Psychiatry, 1977. **134**(12): p. 1408-10.
46. Davis, G.C., W.E. Bunney, E.G. DeFraites, J.E. Kleinman, D.P. van Kammen, R.M. Post, et al., *Intravenous naloxone administration in schizophrenia and affective illness.* Science, 1977. **197**(4298): p. 74-77.
47. Janowsky, D.S., D.S. Segal, A. Abrams, F. Bloom, and R. Guillemin, *Negative naloxone effects in schizophrenic patients.* Psychopharmacology, 1977. **53**(3): p. 295-297.
48. Watson, S.J., P.A. Berger, H. Akil, M.J. Mills, and J.D. Barchas, *Effects of naloxone on schizophrenia: Reduction in hallucinations in a subpopulation of subjects.* Science, 1978. **201**(4350): p. 73-76.
49. Gitlin, M.J., R.H. Gerner, and M. Rosenblatt, *Assessment of naltrexone in the treatment of schizophrenia.* Psychopharmacology, 1981. **74**(1): p. 51-53.
50. Berger, P., S. Watson, H. Akil, and J. Barchas, *The effects of naloxone in chronic schizophrenia.* Am J Psychiatry, 1981. **138**(7): p. 913-918.
51. Rapaport, M.H., O. Wolkowitz, J.R. Kelsoe, C. Pato, P.E. Konicki, and D. Pickar, *Beneficial effects of nalmefene augmentation in neuroleptic-stabilized schizophrenic patients.* Neuropsychopharmacology, 1993. **9**(2): p. 111-115.
52. Gunne, L.-M., L. Lindström, and L. Terenius, *Naloxone-induced reversal of schizophrenic hallucinations.* Journal of neural transmission, 1977. **40**(1): p. 13-19.
53. Volavka, J., A. Mallya, S. Baig, and J. Perez-Cruet, *Naloxone in chronic schizophrenia.* Science, 1977. **196**(4295): p. 1227-1228.
54. Gunne, L.-M., L. Landström, and E. Widerlöv, *Possible role of endorphins in schizophrenia and other psychiatric disorders*, in *Endorphins in Mental Health Research*, E. Usdin, W.E. Bunney, and N.S. Kline, Editors. 1979, Palgrave Macmillan UK: London. p. 545-552.
55. Lipinski, J., R. Meyer, C. Kornetsky, and B. Cohen, *Naloxone in schizophrenia: Negative result.* The Lancet, 1979. **313**(8129): p. 1292-1293.
56. Lehmann, H., V. Nair, and N. Kline, *beta-Endorphin and naloxone in psychiatric patients: clinical and biological effects.* The American journal of psychiatry, 1979. **136**(6): p. 762-766.
57. Freeman, C. and C. Fairburn, *Lack of effect of naloxone and schizophrenic auditory hallucinations.* Psychological medicine, 1981. **11**(2): p. 405-407.
58. Kleinman, J.E., D.R. Weinberger, A. Rogol, D.J. Shiling, W.B. Mendelson, G.C. Davis, et al., *Naloxone in chronic schizophrenic patients: neuroendocrine and behavioral effects.* Psychiatry research, 1982. **7**(1): p. 1-7.

59. Pickar, D., F. Vartanian, W.E. Bunney, H.P. Maier, M.T. Gastpar, R. Prakash, et al., *Short-term naloxone administration in schizophrenic and manic patients: A World Health Organization collaborative study.* Archives of General Psychiatry, 1982. **39**(3): p. 313-319.
60. Naber, D., U. Münch, J. Wissmann, R. Grosse, R. Ritt, and D. Welter, *Naloxone treatment for five days ineffective in schizophrenia.* Acta Psychiatrica Scandinavica, 1983. **67**(4): p. 265-271.
61. Lo, C., H. Wen, and W. Ho, *Cerebrospinal fluid [Met5] enkephalin level in schizophrenics during treatment with naloxone.* European journal of pharmacology, 1983. **92**(1-2): p. 77-81.
62. Cohen, M.R., D. Pickar, and R.M. Cohen, *High-dose naloxone administration in chronic schizophrenia.* Biological psychiatry, 1985. **20**(5): p. 573-575.
63. Pickar, D., W. Bunney, P. Douillet, B. Sethi, M. Sharma, M. Vartanian, et al., *Repeated naloxone administration in schizophrenia: a phase II World Health Organization study.* Biological psychiatry, 1989. **25**(4): p. 440-448.
64. Marchesi, G., G. Santone, P. Cotani, A. Giordano, and F. Chelli, *Naltrexone in chronic negative schizophrenia.* Clinical neuropharmacology, 1992. **15**(Part A): p. 56A-57A.
65. Marchesi, G.F., G. Santone, P. Cotani, A. Giordano, and F. Chelli, *The therapeutic role of naltrexone in negative symptom schizophrenia.* Progress in Neuro-Psychopharmacology and Biological Psychiatry, 1995. **19**(8): p. 1239-1249.
66. Petrakis, I.L., S. O'Malley, B. Rounsaville, J. Poling, C. McHugh-Strong, and J.H. Krystal, *Naltrexone augmentation of neuroleptic treatment in alcohol abusing patients with schizophrenia.* Psychopharmacology, 2004. **172**(3): p. 291-297.
67. Tatari F, S.J., Farnia V, Hashemian A, Rezaei M, et al., *Naltrexone Augmentation of Risperidone in Treatment of Schizophrenia Symptoms: a Randomized Placebo-Controlled Study. .* Ann Psychiatry Ment Health 2014. **2**(3).
68. Simpson, G., M. Branchey, and J. Lee, *TRIAL OF NALTREXONE IN CHRONIC-SCHIZOPHRENIA.* CURRENT THERAPEUTIC RESEARCH-CLINICAL AND EXPERIMENTAL, 1977. **22**(6): p. 909-913.
69. Mielke, D.H. and D.M. Gallant, *An oral opiate antagonist in chronic schizophrenia: a pilot study.* The American journal of psychiatry, 1977.
70. Kline, N.S., C.H. Li, H.E. Lehmann, A. Lajtha, E. Laski, and T. Cooper, *β-Endorphin-induced changes in schizophrenic and depressed patients.* Archives of General Psychiatry, 1977. **34**(9): p. 1111-1113.
71. Orr, M. and C. Oppenheimer, *Effects of naloxone on auditory hallucinations.* British medical journal, 1978. **1**(6111): p. 481.
72. Gitlin, M. and M. Rosenblatt, *Possible withdrawal from endogenous opiates in schizophrenics.* The American journal of psychiatry, 1978.
73. Akil, H., S.J. Watson, P.A. Berger, and J.D. Barachas, *Endorphins. β-LPH, and ACTH: Biochemical, pharmacological and anatomical studies*, in *Advances in Biochemical Psychopharmacology*, C.a. Trabucchi, Editor. 1978, Raven Press: New York. p. 125-139.
74. Dysken, M. and J. Davis, *Naxolone in amylobarbitone-responsive catatonia.* British Journal of Psychiatry, 1978. **133**(11).
75. Abrams, A., D. Braff, D. Janowsky, S. Hall, and D. Segal, *Unresponsiveness of catatonic symptoms to naloxone.* Pharmacopsychiatry, 1978. **11**(04): p. 177-179.
76. Schenk, G., P. Enders, M. Engelmeier, T. Ewert, S. Herdemerten, K. Köhler, et al., *Application of the morphine antagonist naloxone in psychic disorders.* Arzneimittel-Forschung, 1978. **28**(8): p. 1274.

77. Davis, G.C., W.E. Bunney Jr, M.S. Buchsbaum, E. Defraites, W. Duncan, J. Gillin, et al., *Use of narcotic antagonists to study the role of endorphins in normal and psychiatric patients*, in *Endorphins in mental health research*. 1979, Springer. p. 393-406.
78. Watson, S.J., H. Akil, P.A. Berger, and J.D. Barchas, *Some observations on the opiate peptides and schizophrenia.* Archives of General Psychiatry, 1979. **36**(1): p. 35-41.
79. Ragheb, M., S. Berney, and T. Ban, *Naltrexone in chronic schizophrenia.* International pharmacopsychiatry, 1980. **15**: p. 1-5.
80. Sethi, B.B. and R. Prakash, *A study of naloxone with schizophrenic and manic patients.* The British Journal of Psychiatry, 1981. **138**(6): p. 501-503.
81. Jørgensen, H.A. and C. Cappelen, *Naloxone-induced reduction of schizophrenic symptoms A CASE REPORT.* Acta Psychiatrica Scandinavica, 1982. **65**(5): p. 370-374.
82. Naber, D. and K. Leibl, *Repeated high dosage naloxone treatment without therapeutic efficacy in schizophrenic patients.* Pharmacopsychiatry, 1983. **16**(02): p. 43-45.
83. Mongan, L. and E. Callaway, *Buprenorphine responders.* Biological psychiatry, 1990. **28**(12): p. 1078-1080.
84. Groves, S. and D.J. Nutt, *Buprenorphine and schizophrenia.* Human Psychopharmacology: Clinical and Experimental, 1991.
85. Nishikawa, T., A. Tsuda, M. Tanaka, M. Nishikawa, I. Koga, and Y. Uchida, *Naloxone attenuates drinking behavior in a schizophrenic patient displaying self-induced water intoxication.* Clinical neuropharmacology, 1992.
86. Becker, J.A., M.B. Goldman, M.Y. Alam, and D.J. Luchins, *Effects of naltrexone on mannerisms and water imbalance in polydipsic schizophrenics: a pilot study.* Schizophrenia research, 1995. **17**(3): p. 279-282.
87. Nishikawa, T., A. Tsuda, M. Tanaka, M. Nishikawa, I. Koga, and Y. Uchida, *Involvement of the endogenous opioid system in the drinking behavior of schizophrenic patients displaying self-induced water intoxication: a double-blind controlled study with naloxone.* Clinical neuropharmacology, 1996. **19**(3): p. 252-258.
88. Sernyak, M.J., W.M. Glazer, G.R. Heninger, D.S. Charney, S.W. Woods, I.L. Petrakis, et al., *Naltrexone augmentation of neuroleptics in schizophrenia.* Journal of clinical psychopharmacology, 1998. **18**(3): p. 248-251.
89. Wonodi, I., H. Adami, J. Sherr, M. Avila, L.E. Hong, and G.K. Thaker, *Naltrexone treatment of tardive dyskinesia in patients with schizophrenia.* Journal of clinical psychopharmacology, 2004. **24**(4): p. 441-445.
90. Petrakis, I.L., J. Poling, C. Levinson, C. Nich, K. Carroll, B. Rounsaville, et al., *Naltrexone and disulfiram in patients with alcohol dependence and comorbid psychiatric disorders.* Biological Psychiatry, 2005. **57**(10): p. 1128-1137.
91. SARDARPOUR, G.S., M. ARBABI, and A.S. SAMIMI, *Naltrexone as an Adjunct to Treatment of Schizophrenia: A Double Blind Placebo Controlled Trial.* 2006.
92. Batki, S.L., J.A. Dimmock, M. Wade, P.W. Gately, M. Cornell, S.A. Maisto, et al., *Monitored naltrexone without counseling for alcohol abuse/dependence in schizophrenia-spectrum disorders.* American Journal on Addictions, 2007. **16**(4): p. 253-259.
93. Leontieva, L., J. Dimmock, M. Cavallerano, S. DeRycke, Z. Meszaros, K. Carey, et al., *Patient and Provider Attitudes towards Monitored Naltrexone Treatment of Alcohol Dependence in Schizophrenia.* The American journal of drug and alcohol abuse, 2009. **35**(5): p. 273-278.
94. Vasile, D., O. Vasiliu, A.G. Mangalagiu, B.M. Petrescu, G.A. Sopterean, and R.E. Bratu, *P.3.b.001 Pharmacological management of alcohol dependence in chronic schizophrenia.* European Neuropsychopharmacology, 2013. **23**: p. S429.
95. Taveira, T.H., W.-C. Wu, E. Tschibelu, D. Borsook, D.C. Simonson, R. Yamamoto, et al., *The effect of naltrexone on body fat mass in olanzapine-treated schizophrenic or*

schizoaffective patients: a randomized double-blind placebo-controlled pilot study. Journal of Psychopharmacology, 2014. **28**(4): p. 395-400.
96. Bratu, E. and G. Sopterean, *P. 3.020 Comparison of the efficacies of naltrexone and acamprosate in the treatment of patients with chronic schizophrenia who are alcohol dependent.* European Neuropsychopharmacology, 2014(24): p. S71-S72.
97. Tek, C., J. Ratliff, E. Reutenauer, R. Ganguli, and S.S. O'Malley, *A randomized, double-blind, placebo-controlled pilot study of naltrexone to counteract antipsychotic-associated weight gain: proof of concept.* Journal of clinical psychopharmacology, 2014. **34**(5): p. 608.
98. Vasile, D., O. Vasiliu, G. Sopterean, R. Bratu, F. Androne, and F. Vasile, *P. 3. d. 028 Effectiveness and tolerability of nalmefene in alcohol use dependence comorbid with schizophrenia.* European Neuropsychopharmacology, 2014. **24**: p. S538.
99. Janowsky, D.S., D.S. Segal, F. Bloom, A. Abrams, and R. Guillemin, *Lack of effect on naloxone on schizophrenic symptoms.* Am J Psychiatry, 1977. **134**(8): p. 926-7.
100. Davis, G.C., W.C. Duncan, J.C. Gillin, and W.E. Bunney, Commun. Psychopharmac, 1977. **1**: p. 489-492.
101. Emrich, H.M., Laspe, H., Cording, C., *A possible antipsychotic action of the specific opiate antagonist naloxone.* Neuroscience Letters, 1978. **10**(suppl. 1).
102. Janowsky, D.S., L.L. Judd, and L. Huey, *Effects of naloxone in normal, manic, and schizophrenic patients: evidence for alleviation of manic symptoms*, in *Endorphins in mental health research*. 1979, Springer. p. 435-447.
103. Perez-Cruet, J., J. Volavka, A. Mallya, S. Baig, and A. Toga, *Behavioral effects of naloxone and LSD*, in *Endorphins in Mental Health Research*. 1979, Springer. p. 407-415.
104. Volavka, J., L.G. Davis, and Y.H. Ehrlich, *Endorphins, dopamine, and schizophrenia.* Schizophrenia Bulletin, 1979. **5**(2): p. 227-239.
105. Davis, G.C., Buchsbaum, M.S., Bunney Jr., W.E., *Research in endorphins and schizophrenia.* Schizophrenia Bulletin, 1979. **5**(2): p. 244.
106. Berger, P.A., S.J. Watson, H. Akil, and J.D. Barchas, *Naloxone administration in chronic hallucinating schizophrenic patients*, in *Endorphins in mental health research*. 1979, Springer. p. 423-434.
107. Emrich, H., C. Cording, S. Piree, A. Kölling, H.-J. Möller, D. Von Zerssen, et al., *Actions of naloxone in different types of psychoses*, in *Endorphins in mental health research*. 1979, Springer. p. 452-460.
108. Lehmann, H.E., T.H. Cooper, N.P.V. Nair, and N.S. Kline, *Beta-Endorphins and Naloxone in Psychiatric Patients: Clinical and Biological Effects*. 1979. 535-539.
109. Verebey, K. and S. Mulé, *Naltrexone, 6 beta-naltrexol and 2-hydroxy-3-methoxy-6 beta-naltrexol plasma levels in schizophrenic patients after large oral doses of naltrexone.* NIDA research monograph, 1979. **27**: p. 296-301.
110. Verebey, K. and S. Mule, *NALTREXONE AND BETA-NALTREXOL PLASMA-LEVELS IN SCHIZOPHRENIC-PATIENTS AFTER LARGE ORAL DOSES OF NALTREXONE.* RESEARCH COMMUNICATIONS IN PSYCHOLOGY PSYCHIATRY AND BEHAVIOR, 1979. **4**(3): p. 311-317.
111. Barchas, J., P. Berger, S. Watson, H. Akil, and C. Li, *Opioid agonists and antagonists in schizophrenia.* Advances in biochemical psychopharmacology, 1980. **22**: p. 447.
112. Malek-Ahmadi, P. and K.E. Callen, *Endorphins and schizophrenia—Narcotic antagonists in the treatment of chronic schizophrenia.* General Pharmacology: The Vascular System, 1980. **11**(2): p. 149-151.
113. Lideman, R.R., G.P. Panteleeva, M.Y. Cuculkovskaya, F.E. Vartanian, and B.S. Belyaev, Zh. Nevropat. Psikhiat., 1980. **80**: p. 231-237.

114. Verhoeven, W., H. Van Praag, and J. De Jong, *Use of naloxone in schizophrenic psychoses and manic syndromes.* Neuropsychobiology, 1981. **7**(3): p. 159-168.
115. Ananth, J., V. Nair, and R. Rastogi, *Opiate Receptors and Opiate Antagonists in Psychiatric and Related Research A Review*, in *Endorphins and Opiate Antagonists in Psychiatric Research*. 1982, Springer. p. 179-212.
116. Mielke, D.H., *The use of an oral opiate antagonist in schizophrenia*, in *Endorphins and Opiate Antagonists in Psychiatric Research*. 1982, Springer. p. 305-309.
117. Verhoeven, W. and H.M.v. Praag, *Short-term naloxone administration in schizophrenic and manic patients.* Archives of general psychiatry, 1982. **39**.
118. Volavka, J., B. Anderson, and G. Koz, *Naloxone and naltrexone in mental illness and tardive dyskinesia.* Annals of the New York Academy of Sciences, 1982. **398**(1): p. 97-102.
119. Handal, K.A., J.L. Schauben, and F.R. Salamone, *Naloxone.* Annals of Emergency Medicine, 1983. **12**(7): p. 438-445.
120. Mueser, K.T. and M.W. Dysken, *Narcotic antagonists in schizophrenia: A methodological review.* Schizophrenia Bulletin, 1983. **9**(2): p. 213-225.
121. Lideman, R.R., G.P. Panteleeva, M.Y. Tsutsul'kovskaya, F.E. Vartanyan, and B.S. Belyaev, *Effect of naloxone on the state of patients with endogenous psychoses.* Neuroscience and Behavioral Physiology, 1984. **14**(6): p. 471-475.
122. Verhoeven, W.M., H.M. Van Praag, and J.M. Van Ree, *Repeated naloxone administration in schizophrenia.* Psychiatry research, 1984. **12**(4): p. 297-312.
123. Goldbloom, D.S., *Endogenous opiates and schizophrenia: directions in clinical research.* The Canadian Journal of Psychiatry, 1984. **29**(4): p. 355-360.
124. McNicholas, L. and W. Martin, *New and experimental therapeutic roles for naloxone and related opioid antagonists.* Drugs, 1984. **27**(1): p. 81-93.
125. Nemeroff, C.B. and G. Bissette, *Neuropeptides, dopamine, and schizophrenia.* Annals of the New York Academy of Sciences, 1988. **537**(1): p. 273-291.
126. Budd, K., *Clinical use of opioid antagonists.* Baillière's Clinical Anaesthesiology, 1987. **1**(4): p. 993-1011.
127. Marchesi, G., G. Santone, P. Cotani, and G. Troiani, *Naltrexone integrated neuroleptic treatment in schizophrenia.* Biol. Psychiatry, 1991. **29**(s11): p. 536.
128. Marchesi, G., G. Santone, P. Cotani, A. Giordano, and F. Chelli, *Opioid peptide receptor blockade in the treatment of schizophrenia.* European Neuropsychopharmacology, 1992. **2**(3): p. 365-366.
129. Welch, E. and D. Thompson, *Opiate antagonists for the treatment of schizophrenia.* Journal of clinical pharmacy and therapeutics, 1994. **19**(5): p. 279-283.
130. Modesto-Lowe, V. and J. Van Kirk, *Clinical uses of naltrexone: A review of the evidence.* Experimental and Clinical Psychopharmacology, 2002. **10**(3): p. 213.
131. Petrakis, I.L., C. Nich, and E. Ralevski, *Psychotic Spectrum Disorders and Alcohol Abuse: A Review of Pharmacotherapeutic Strategies and a Report on the Effectiveness of Naltrexone and Disulfiram.* Schizophrenia Bulletin, 2006. **32**(4): p. 644-654.
132. Ralevski, E., K. Balachandra, R. Gueorguieva, D. Limoncelli, and I. Petrakis, *Effects of naltrexone on cognition in a treatment study of patients with schizophrenia and comorbid alcohol dependence.* Journal of dual diagnosis, 2006. **2**(4): p. 53-69.
133. Batki, S.L., Z.S. Meszaros, K. Strutynski, J.A. Dimmock, L. Leontieva, R. Ploutz-Snyder, et al., *Medical comorbidity in patients with schizophrenia and alcohol dependence.* Schizophrenia research, 2009. **107**(2-3): p. 139-146.
134. Tatari, F., J. Shakeri, V. Farnai, A. Hashemain, M. Rezaei, N. Abdoli, et al., *Naltrexone Augmentation of Risperidone in Treatment of Schizophrenia Symptoms.* Technical Journal of Engineering and Applied Sciences, 2015.

135. Azorin, J.-M., N. Simon, M. Adida, and R. Belzeaux, *Pharmacological treatment of schizophrenia with comorbid substance use disorder.* Expert opinion on pharmacotherapy, 2016. **17**(2): p. 231-253.
136. Bennett, M.E., K.R. Bradshaw, and L.T. Catalano, *Treatment of substance use disorders in schizophrenia.* The American journal of drug and alcohol abuse, 2017. **43**(4): p. 377-390.
137. Sawicka, M. and D.K. Tracy, *Naltrexone efficacy in treating alcohol-use disorder in individuals with comorbid psychosis: a systematic review.* Therapeutic advances in psychopharmacology, 2017. **7**(8-9): p. 211-224.
